# Design and Synthesis of Ketoconazole Derivatives as Innovative Anti‐Infective Agents

**DOI:** 10.1002/ardp.70062

**Published:** 2025-07-29

**Authors:** Gioele Renzi, Andrea Angeli, Silvia Selleri, Costanza Spadini, Nicolo’ Mezzasalma, Marcus T. Hull, Steven L. Kelly, Clemente Capasso, Clotilde S. Cabassi, Fabrizio Carta, Claudiu T. Supuran

**Affiliations:** ^1^ NEUROFARBA Department, Section of Pharmaceutical and Nutraceutical Sciences, Sesto Fiorentino Università degli Studi di Firenze Florence Italy; ^2^ Department of Veterinary Science University of Parma Parma Italy; ^3^ Faculty of Medicine, Health and Life Science, Institute of Life Science Swansea University Swansea UK; ^4^ Department of Biology, Agriculture and Food Sciences Institute of Biosciences and Bioresources Napoli Italy

**Keywords:** 14α‐demethylase, antifungals, carbonic anhydrases, fungi, *Malassezia*

## Abstract

A novel series of compounds was designed and synthesized by combining the distal piperazine nitrogen of the antifungal ketoconazole (KTZ) with primary arylsulfonamides. The aim of this study is to present the basis for a new generation of *Malassezia* antifungal agents able to inhibit the enzyme lanosterol‐14α‐demethylase (CYP51; EC 1.14.13.70) as well as a newly emergent therapeutic target: carbonic anhydrases (CAs; EC 4.2.1.1). The final compounds showed effective interactions with the intended targets in vitro, as well as KTZ comparable minimum inhibitory concentrations on yeast strains of the *Malassezia* genus: *Malassezia furfur* ATCC 14521; *Malassezia globosa* ATCC MYA 4612; and *Malassezia pachydermatis* DSM 6172. Overall, the data obtained account for the reported compounds as promising antifungal candidates with high safety profiles for the management of fungal infections.

## Graphical abstract

A series of ketoconazole‐based carbonic anhydrase (CA) inhibitors were synthesized and characterized, and their inhibitory activities were assessed both against human and fungal CA. The novel compounds showed antifungal activity and minimal inhibitory concentrations comparable to the reference standard ketoconazole, with higher safety profiles when tested on human keratinocytes at maximal dosages.

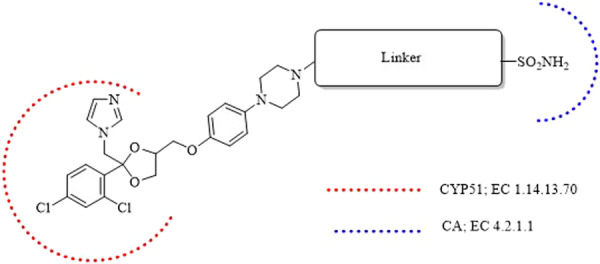

## 1 Introduction

**Figure 1:**
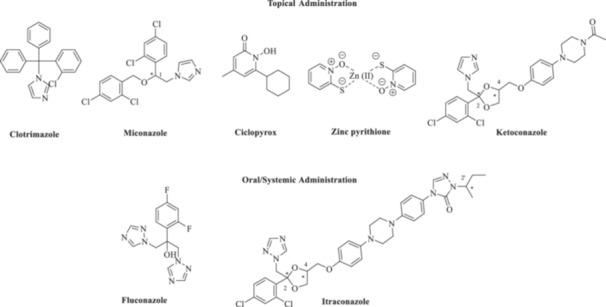
Chemical structures of drugs used for the management of *Malassezia spp*.‐promoted infections. * denotes chiral centers.

*Malassezia* species (*spp*.) are high lipophilic yeasts symbiotically present as part of the natural microbiota on the skin of mammals particularly rich in sebum (i.e., in humans, the scalp, face, and chest among others) [1]. Any physical or physiological cause that disrupts the microbioma equilibrium may lead to *Malassezia spp*. overgrowth, which in turn triggers acute infections such as pityriasis versicolor, seborrheic dermatitis, dandruff, and folliculitis, among others [2, 3]. Such diseases may spread over the host surface as well as deeper within tissues with significant systemic effects when adequate pharmacological treatments are absent [2, 3]. *Malassezia spp*., such as *Malassezia furfur*, *Malassezia globosa*, *Malassezia restricta*, and *Malassezia sympodialis,* are the most common yeasts on human skin [3]. In animals, *Malassezia pachydermatis* is the most represented yeast in the cutaneous mycobiome, both as a commensal and as a pathogen, and its overgrowth is related to the onset of severe dermatitis and otitis externa in companion animals, mainly dogs and cats, and therefore assumes substantial clinical significance [4, 5]. The standard of care treatment of *Malassezia*‐promoted infections largely depends on the specific clinical conditions, but more in general, any pharmacological approach accounts for the use of antifungal agents targeting the etiological yeast [1, 6, 7]. Representative drugs used for human and veterinary purposes are azole‐containing derivatives shown in Figure 1, which are quite effective in inhibiting the cytochrome P450 14α‐demethylase enzyme (CYP51; EC 1.14.13.70). As a result, such compounds affect the conversion of lanosterol into ergosterol and compromise the specific lipidomic composition in yeast biomembranes.

Compounds such as ketoconazole (KTZ) and itraconazole (ITZ) contain multiple stereogenic centers and are therefore associated with extra costs of production and quality assurance. For instance, in KTZ and ITZ, the triazolomethylene and aryloxymethylene moieties anchored to the dioxolane ring are always fixed in *cis* relation to each another, thus yielding the clinical formulation *cis*‐(2*S*,4 *R*)‐(‐) and *cis*‐(2 *R*,4*S*)‐(+) enantiomers in a 1/1 *ratio* for the former [8] and *cis*‐(2 *R*, 4*S*, 2′*R*), *cis*‐(2 *R*, 4*S*, 2′*S*), *cis*‐(2*S*, 4 *R*, 2′*R*), and *cis*‐(2*S*, 4 *R*, 2′*S*) in a1/1/1/1 *ratio* of four stereoisomers (two enantiomeric pairs) for the latter [9]. In addition, the clinical effectiveness of azole antifungals is constantly eroded by the long latency time necessary to establish reliable therapeutic responses, which, however, does not cope with the high variability of genomes and quick reproduction rates typical of fungi/yeasts [10, 11]. As a result, azole resistance leads to the constant growth of fungal‐promoted infections in humans, animals, and plants and governs the need for more effective therapeutic tools [12]. In this study, we sought to make use of our know‐how relative to the validation of the fungal‐expressed metalloenzyme carbonic anhydrases (CAs, E.C. 4.2.1.1) target [13, 14, 15, 16, 17] by merging the prototypic primary sulfonamide CA inhibitory (CAI) moiety with a clinically used azole drug. The primary aim of our work is to explore the chemical feasibility of our strategy and to prove antifungal effectiveness, also in comparison to the parent drugs, of our final compounds. Specifically, we focused our attention on the chemically modifiable KTZ drug by adopting standard medicinal chemistry derivatization strategies.

## 2 Results and Discussion

### 2.1 Design and Synthesis of Compounds

**Scheme 1:**
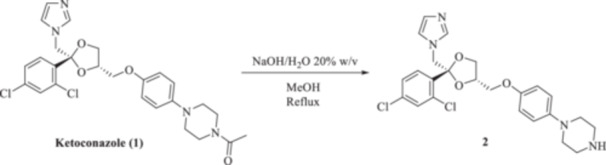
Synthesis of the reactive ketoconazole intermediate **2**.

We aimed to develop an efficient synthetic approach for obtaining KTZ‐based CAI compounds through elongation of the antifungal molecular structure. For instance, we sought to make use of the unprotected piperazine distal nitrogen to establish covalent bonds with a variety of scaffolds incorporating the prototypic CAI moiety of the primary sulfonamide type [18]. For such purposes, the commercially available *cis*‐(2 *R*,4*S*)‐( + ) KTZ **1** enantiomer was deacetylated under basic conditions to afford the valuable synthetic intermediate **2** in a 91% yield on a multigram gram‐scale reaction (Scheme 1).

**Scheme 2:**
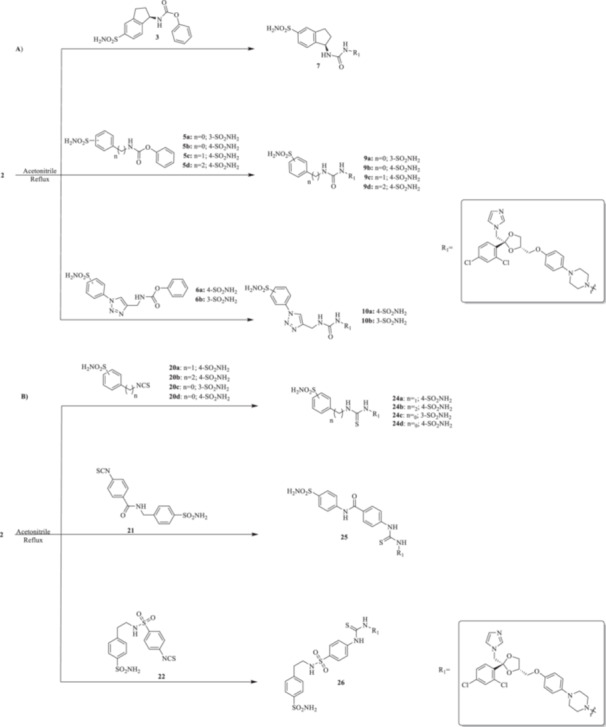
(A, B) Synthesis of the ketoconazole derivatives **7**, **9a–d**, **10a–b**, **24a–d**, **25**, and **26**.

**Scheme 3:**
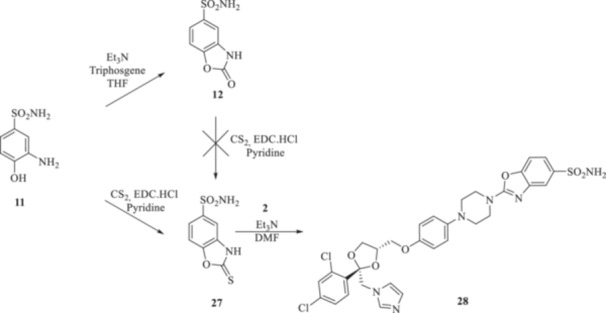
Synthesis of intermediates **12** and **27**, and of the final compound **28**.

**Scheme 4:**
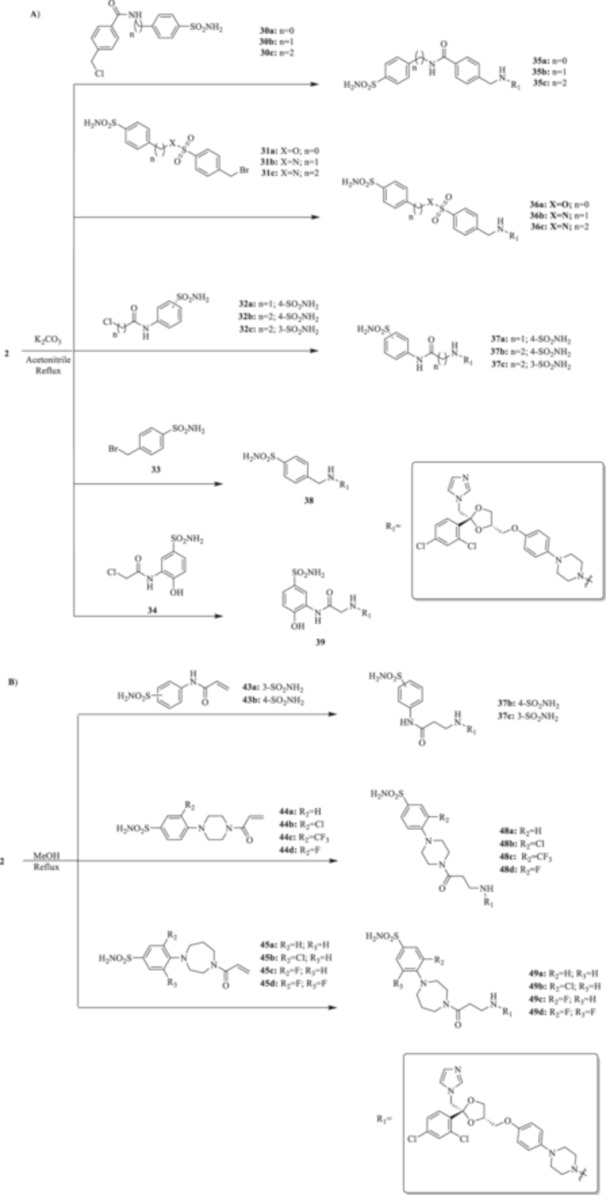
(A, B) Synthesis of the ketoconazole derivatives **35a–c**, **36a–c**, **37a–c**, **38**, **39**, **48a–d**, and **49a–d**.

All the final compounds reported in this study were obtained by direct coupling of **2** with ad hoc and freshly synthesized intermediates as reported in Schemes 2, 3, 4, 3. (N.B. The synthesis and characterization of intermediates are reported in the Supporting Information file). In particular, the ureidic connection to afford **7**, **9a–d**, and **10a–b** was obtained by nucleophilic additions of **2** with the appropriate phenyl carbamates **3**, **5a–d**, and **6a–b**, respectively. On the other hand, the thioureido moiety in **24a–d**, **25**, and **26** was positioned by reacting **2** with the corresponding isothiocyanates **20a–d**, **21**, and **22**, respectively (Schemes 2, 3).

Additionally, we explored the synthetic feasibility of benzoxazolyl‐containing compounds with the intent of elongating the CAI‐directed warhead by means of a valuable heteroaromatic scaffold. At first, intramolecular cyclization of the commercially available *ortho*‐amino phenol **11** to afford the key derivative 2‐oxo‐benzoxazolyl **12** was operated, which in turn was exposed to carbon disulfide (CS_2_) reaction conditions with the intent of positioning the reactive 2‐thioxo‐moiety as in **27** (Scheme 3).

Although thionation attempts on **12** were unsuccessful, to our surprise, the desired intermediate **27** was obtained in a 64% yield by direct treatment of **11** with CS_2_ and *N*‐(3‐dimetilaminopropyl)‐*N*′‐etilcarbodiimide hydrochloride (EDC. HCl). Then, the final compound **28** was obtained in a 26% yield by the addition of **2** on **27** under basic conditions for triethylamine (Et_3_N).

The nucleophilic features of **2** toward the halo‐substituted benzyl/alkyl derivatives **30a–c**, **31a–c**, **32a–c**, **33**, and **34** yielded the final **35a–c**, **36a–c**, **37a–c**, **38**, and **39**, respectively, as shown in Scheme 4A.

The piperazine moiety in **2** was successfully investigated to perform 1,4‐conjugate additions on acrylate intermediates **43a–b**, **44a–d**, and **45a–d** to yield **37b–c**, **48a–d**, and **49a–d**, respectively (Scheme 4B). It is noteworthy that derivatives **37b** and **37c** were obtained through both synthetic strategies in Scheme 4A,B with comparable recovery yields. Unfortunately, it was not feasible to obtain **50** under the various reaction conditions explored.

All the final KTZ‐CAIs compounds reported herein were purified by silica gel column chromatography using the appropriate eluting mixtures, followed by trituration or recrystallization as needed (see Section 4). Full characterization was conducted by means of solution ^1^H‐ and ^13^C‐NMR. Elemental analyses account for a purity grade ≥ 96%.

### 2.2 CA In Vitro Enzymatic Assay

All the final compounds were screened in vitro using the stopped‐flow CO_2_ hydration assay [19] to assess their inhibitory activity and selectivity profiles on the fungal β‐CAs expressed from the *Malassezia globosa* (MgCA), *Malassezia pachydermatis* (MpaCA), and *Malassezia restricta* (MreCA) genus. Human (h) CAs I, II, IX, and XII were assumed to be off‐target isoforms. All the obtained data are reported in Table 1 as *K*_I_ values and are compared to the primary sulfonamide drug acetazolamide (AAZ) as the reference compound.

**Table 1:** Inhibition data of compounds 7, 9a–d, 10a–b, 24a–d, 25, 26, 28, 35a–c, 36a–c, 37a–c, 38, 39, 48a–d, and 49a–d on fungal MgCA, MpaCA, MreCA, hCAs I, II, IX, and XII. AAZ was considered as the reference drug [19].

*K*_I_ (nM)^a^							
Cmp	MgCA	MpaCA	MreCA	hCA I	hCA II	hCA IX	hCA XII
**7**	69497	513.7	206.2	5912	5847	4.4	63.1
**9a**	43809	601.9	90.5	1622	1864	15.1	39.6
**9b**	5489	516.7	613.9	337.6	71.8	32.9	8.2
**9c**	5633	821.2	683.9	437.2	58.9	13.9	6.4
**9d**	3333	291.6	281.7	84.0	563.4	6.0	9.5
**10a**	7578	4202	892.6	870.2	390.0	38.0	44.0
**10b**	5045	702.1	360.6	896.5	533.9	41.2	7.4
**24a**	7000	4944	339.2	862.9	605.0	47.7	72.5
**24b**	6866	841.7	462.7	871.7	258.0	48.7	9.2
**24c**	8405	604.2	924.5	328.2	392.9	40.1	8.0
**24d**	4482	610.9	825.2	861.0	736.6	4.4	8.2
**25**	6058	930.7	620.6	871.5	882.6	41.0	9.0
**26**	6027	2390	338.2	58.3	17.3	151.8	62.3
**28**	4570	380.9	680.8	944.0	8.3	28.3	47.2
**35a**	4249	803.3	683.6	352.5	26.5	46.8	8.2
**35b**	4604	695.0	701.7	4767	620.0	368.6	64.4
**35c**	4473	646.6	318.3	5515	55.7	47.9	7.5
**36a**	5429	577.0	288.2	2662	180.0	311.5	5.9
**36b**	4062	559.7	451.1	3418	1196	41.9	6.2
**36c**	6124	677.9	815.8	2986	938.8	43.9	8.1
**37a**	5344	5931	398.2	497.4	21.4	346.3	61.0
**37b**	6484	891.7	691.9	9238	798.6	49.1	9.1
**37c**	7435	1927	280.2	4351	92.6	331.4	9.4
**38**	5810	553.8	473.1	382.0	122.0	224.8	9.3
**39**	5538	627.8	390.5	817.8	77.1	76.0	6.3
**48a**	5562	679.4	403.5	82.1	49.1	30.6	3.7
**48b**	6479	830.4	472.4	769.7	20.7	48.4	31.1
**48c**	7208	721.5	416.6	835.8	167.9	445.7	9.4
**48d**	8036	630.3	471.4	363.9	44.6	306.3	56.1
**49a**	56446	459.3	410.9	315.1	288.5	13.8	5.5
**49b**	6855	733.0	337.3	96.1	195.6	272.1	67.9
**49c**	8184	877.6	470.2	5.3	7.2	284.6	7.9
**49d**	4391	2583	767.9	777.0	247.4	2167	55.9
AAZ	40000	620.0	100.0	250.0	12.1	25.8	5.7

^a^ Mean from three different assays, using the stopped flow technique (errors were in the range of ± 5–10% of the reported values).

Overall, the data in Table 1 accounted for the hCA isoforms preferentially inhibited over the fungal ones from the panel of compounds reported in this study.

i. Deeper insight revealed that ureido **7**, **9a–d**, and **10a–b** were slightly active toward MgCA, with *K*_I_s spanning between 3333 and 69497 nM. Conversely, the affinity for the remaining fungal β‐CAs was increased up to medium nanomolar values. For instance, *K*_I_s for the MpaCA ranged between 291.6 and 4202 nM. A slight regioisomeric effect was observed for the **9a**/**9b** regioisomeric pair, with the 4‐substituted being a 1.2‐fold more effective inhibitor and almost equipotent to derivative **7** (i.e., *K*_I_s of 513.7 and 516.7 nM for **7** and **9b**, respectively). Elongation of the alkyl chain in **9b** up to two carbon atoms (i.e., **9d**) was beneficial for the inhibition potency, as the lowest inhibition value was obtained for the MpaCA among the ureido‐containing compounds (i.e., *K*_I_ of 291.6 nM for **9d**). Significant regioisomeric effects on the kinetics were reported for the ureido derivatives **10a** and **10b,** respectively, with the latter being a 6.0‐fold more effective MpaCA inhibitor when compared with its pair (*K*_I_s of 4202 and 702.1 nM for **10a** and **10b**, respectively). Similar values were obtained for the MreCA isoform (i.e., *K*_I_s between 206.2 and 892.6 nM). A strong regioisomeric effect was reported for **9a,** which inhibited the MreCA at a 90.5 nM concentration, and was thus 6.8‐fold stronger than its 4‐substituted counterpart **9b** (*K*_I_ of 613.9 nM). Replacement of the ureido moiety in **9a–d** with the thiourea instead, to afford **24a–d**, did not significantly affect the in vitro kinetic profiles for the fungal CAs. Again, MgCA was the least inhibited, with *K*_I_ values ranging between 4482 and 8405 nM (Table 1). As for the MpaCA, a very slight regioisometic effect was observed for **24c** and **24d** (*K*_I_s of 604.2 and 610.9 nM, respectively), whereas elongation of the tether was far more evident on the kinetics as reported for **24a, b,** and **d** (*K*_I_s of 4944, 841.7, and 610.9 nM for **24a**, **b**, and **d** respectively). A similar trend for **24a–d** was also reported on the MreCA, with *K*_I_ values spanning between 339.2 and 924.5 nM (Table 1). Further elongation of the scaffold as in compounds **25** and **26** yielded *K*_I_ values for the fungal‐expressed CAs in agreement with the compounds mentioned previously. Data in Table 1 clearly show that MgCA was poorly inhibited from both **25** and **26**, with *K*_I_ values of 6058 and 6027 nM, respectively. The same compounds showed good selectivity for the remaining fungal CAs. For instance, **25** was 2.6‐fold more potent than **26** on the MpaCA (*K*_I_s of 930.7 and 2390 nM, respectively), whereas the opposite trend, although with lower intensity, was observed for the MreCA (*K*_I_s of 620.6 and 338.2 nM, respectively). As for the benzoxazolyl‐containing **28**, preferential inhibition for the MpaCA isoform over Mg‐ and MreCAs was reported (*K*_I_s of 380.9, 4750, and 680.8 nM, respectively). Close inhibition potencies were obtained for derivatives **35a–c** on MgCA and were between 4604 and 4473 nM (Table 1). Elongation of the alkyl chain in **35a–c** yielded a progressive increase in the compound's effectiveness for the MpaCA isoform (i.e., *K*_I_s of 803.3, 695.0, and 646.6 nM for **35a–c,** respectively). Marked effects on kinetics were reported for the same compounds on the MreCA, as the longest derivative **35c** was 2.1‐fold more effective than its progenitor **35a**. A close matching trend was also obtained on comparison of **35c** with **35b** (*K*_I_s of 701.7 and 318.3 nM for **35b** and **35c,** respectively). The introduction in **35a–c** of a sulfonyl‐based connection as in **36a–c** induced high variability among the kinetic data in Table 1, although the preferential inhibition for the Mpa‐ and MreCAs was retained. Derivative **36b** was the most effective inhibitor for MgCA, with a *K*_I_ value of 4062 nM, thus being 1.3‐ and 1.5‐fold more potent than **36a** and **36c**, respectively (i.e., *K*_I_s of 5429 and 6124 nM for **36a** and **36c,** respectively). An identical kinetic trend was reported for the MpaCA isoform, although with limited differences (*K*_I_s of 577.0, 559.7, and 677.9 nM for **36a–c**, respectively). As for the MreCA, compound **36a** was the most effective, with a *K*_I_ value of 288.2 nM. Derivatives **36b** and **36c** showed *K*_I_s of 451.1 and 815.8 nM, respectively (Table 1). Among amido derivatives **37a–c**, high kinetic variability was reported. For instance, MgCA was inhibited in a concentration range spanning between 7435 and 5344 nM. Better results were obtained for the remaining fungal‐expressed MpaCA and MreCA isoforms. Elongation of derivative **37a** to afford **37b** enhanced the inhibition potency by up to 6.7‐fold (i.e., *K*_I_s of 5391 and 891.7 nM, respectively). A strong regioisomeric effect was observed among **37b** and **37c** (i.e., *K*_I_s of 891.7 and 1927 nM for **37b** and **37c,** respectively). As for the MreCA, elongation of the chain significantly reduced the inhibition potency by 1.7‐fold (i.e., *K*_I_s of 398.2 and 691.9 nM for **37a** and **37b**, respectively). Also, for this isoform, a strong regioisomeric effect was reported, as compound **37c** was 2.5‐fold more effective inhibitor than its isomer **37b** (i.e., *K*_I_s of 691.9 and 280.2 nM for **37b** and **37c**, respectively). Both derivatives **38** and **39** showed similar kinetic profiles for the fungal‐expressed CAs and were all in agreement with the previously discussed compounds. Methoxy **39** was the most effective inhibitor for MreCA, with a *K*_I_ value of 390.5 nM (Table 1). Derivatives **48a–d** showed very few differences within each isoform. For instance, MgCA was inhibited in a concentration range of 5562–8036 nM, MpaCA was inhibited in a concentration range of 630.3–830.4 nM, and MreCA was inhibited in a concentration range of 403.5–472.4 nM, thus indicating that the substituents incorporated did not significantly affect the binding of the ligand within the catalytic cleft (Table 1). Quite interestingly, introduction of the 1,4‐disubstituted diazepane moiety as in **49a–d** yielded better discrimination of the *K*_I_ values among the series (Table 1). Desymmetrization of the CAI‐directed phenyl ring in **49a** to yield the 3‐chlorophenyl derivative **49b** led to an 8.2‐fold increase in the *K*_I_ associated value for the MgCA isoform (i.e., *K*_I_s of 56446 and 6855 nM for **49a** and **49b,** respectively). The inhibition potency for MgCA decreased slightly by up to 1.2‐fold by substitution of the chloro in **49b** with a fluorine instead to afford derivative **49c** (i.e., *K*_I_s of 6855 and 8184 nM for **49b** and **49c**, respectively). Further improvement of the inhibition potency against MgCA was reported for the 3,5‐difluorosubstituted derivative **49d,** which showed a *K*_I_ value of 4391 nM (Table 1). A different kinetic trend was reported for MpaCA, with **49a** being the most effective inhibitor, with a *K*_I_ value of 459.3 nM. All further manipulations on **49a** to afford **49b–d** induced a sequential increase of the MpaCA associated *K*_I_ values of up to 2583 nM (Table 1). As for MreCA, introduction in **49a** of chloro (**49b**) and fluoro (**49c**) halogens yielded opposite and equivalent effects on the ligand's inhibition potencies. As reported in Table 1, the former was more effective inhibitor by 1.2‐fold and the latter was a less effective inhibitor by 1.4‐fold when compared with unsubstituted **49a** (i.e., *K*_I_s of 410.9, 337.3, and 470.2 nM for **49a**, **49b**, and **49c,** respectively).
ii. Compounds reported herein were profiled in vitro on hCAs I, II, IX, and XII, and the data in Table 1 clearly accounted for the transmembrane CAs being preferentially inhibited over the constitutive and largely expressed I and II isoforms. Among ureido derivatives **9a‐d**, the *K*_I_ associated values for the IX and XII isoforms were within the low nanomolar range (Table 1). The most effective hCA IX inhibitor was **9d,** with a *K*_I_ of 6.0 nM, whereas its shorter derivative **9c** was the most potent ligand for the XII isoform with a close matching *K*_I_ value (i.e., 6.4 nM). It is worth noting that all the 4‐substituted arylsulfonamides **9b–d** showed similar potencies for hCA XII, whereas such a kinetic trend was not retained for the IX isoform (Table 1). As for the remaining isoforms, compounds **9a–d** were medium‐high‐nanomolar‐range inhibitors. Also, in this case, compounds **9c** and **9d** were highly selective for hCA II and I, respectively (i.e., *K*_I_s of 84.0 and 58.9 nM for hCA I and II, respectively). The introduction in **9a–d** of the thioureido ureido group, as in **24a–d**, significantly affected the kinetic trend among the hCAs tested, although preferential inhibition for the membrane IX/XII over the cytosolic I/II was retained. For instance, **24d**, which is the thioureido derivative of **9b**, was the most potent hCA IX inhibitor, with a *K*_I_ value of 4.4 nM, and all the remaining compounds were up to 10.8‐fold less effective (i.e., *K*_I_s spanning between 40.1 and 47.7 nM; Table 1). Closely matching *K*_I_s were reported for **24b–d**, with values spanning between 8.0 and 9.2 nM, whereas derivative **24a** was a 72.5 nM hCA XII inhibitor. As for the hCAs I/II, compounds **24a–d** were high nanomolar inhibitors (Table 1). Insertion of the 1,4‐triazolyl moiety as in **10a** and **10b** retained significant discrimination for hCAs I/II over IX/XII, with narrow differences in the *K*_I_ values due to the regioisomeric effect. It is worth noting that derivative **10b** was the most potent inhibitor of hCA XII, with *K*_I_ of 7.4 nM, and therefore close to the reference AAZ of 5.7 nM. Interestingly, SAR was reported for **25** and **26**. For instance, derivative **25** was selective for the hCA XII isoform at a 9.0 nM concentration. A radical change in isoform selectivity was reported for **26** (i.e., *K*_I_ of 17.3 nM for hCA II). As for the heterocyclic derivative **28**, predominant inhibition of the housekeeping hCA II was observed (*K*_I_ of 8.3 nM), followed by the tumor‐associated hCAs IX and XII, which showed *K*_I_ values of 28.3 and 47.2 nM, respectively (Table 1).

Among the amide‐containing compounds **35a–c**, preferential selectivity for the hCA XII isoform was reported for compounds **35a** and **35c,** with *K*_I_ values of 8.2 and 7.5 nM, respectively, whereas the benzyl intermediate **35b** was a medium nanomolar inhibitor (i.e., *K*_I_ 64.4 nM). A similar kinetic trend was reported for the hCA IX isoform, although the associated *K*_I_ values were in the medium to high nanomolar range (Table 1). Derivative **35b** was poorly effective on hCAs I and II too, with *K*_I_s of 4767 and 620.0 nM, respectively. As for the shortest **35a,** a 26.5 nM inhibition value was reported for hCA II, whereas it was poorly effective on the hCA I isoform (i.e., *K*_I_ of 352.5 nM). Finally, the longest **35c** was a high nanomolar hCA I inhibitor with a *K*_I_ value of 5515 nM; it was effective on the hCA II isoform at a 55.7 nM concentration, and thus almost equipotent to the IX isozyme. A diverse kinetic trend on the hCAs considered in this study was obtained for sulfonyl derivatives **36a–c**. As reported in Table 1, the entire series was highly selective and potent toward the hCA XII isoform, with *K*_I_ values progressively increased from 5.9 nM for **36a** up to 8.1 nM for **36c** (Table 1). As for hCA IX, a 4.1‐fold enhancement in the inhibition potency was obtained when the sulfonyl ester connecting moiety in **36a** was replaced with sulfonamide instead to afford **36b** (i.e., *K*_I_s of 311.5 and 41.9 nM for **36a** and **36b,** respectively), whereas slight elongation of the alkyl tether (i.e., **36c**) did not induce significant effects on kinetics (Table 1). The remaining hCAs I/II were scarcely inhibited from **36a–c**, with *K*_I_ values in the high nanomolar range (Table 1). A clearer kinetic trend was obtained for the amido containing **37a–c**. Elongation of the alkyl tether in **37a** of a single carbon unit to afford **37b** led to loss of effectiveness for hCA II (i.e., *K*_I_s of 21.4 and 798.6 nM for **37a** and **37b,** respectively) and significant gain of inhibition potency for the hCAs IX and XII isoforms (Table 1). For instance, **37b** was a 7.05‐fold more effective hCA IX inhibitor when compared with its shorter counterpart **37a** (i.e., *K*_I_s of 346.3 and 49.1 nM for **37a** and **37b,** respectively); as for the hCA XII, it showed a potency gain of 6.7‐fold (i.e., *K*_I_s of 61.0 and 9.1 nM for **37a** and **37b,** respectively). Regioisomeric effects on kinetics were clearly observed for **37b** and **37c** on all hCAs considered, except for the isoform XII (i.e., *K*_I_s of 9.1 and 9.4 nM for **37b** and **37c,** respectively). Both compounds **38** and **39** retained high selectivity and nanomolar potency for the hCA XII isoform, with *K*_I_ values of 9.3 and 6.3 nM, respectively. As for the remaining hCAs isoforms, it is worth noting that **39** was an almost equipotent inhibitor of the hCA II and IX isoforms, with *K*_I_ values of 77.1 and 76.0 nM, respectively. Heterogenic SAR was reported for the piperazine‐containing derivatives **48a‐d** and depending upon the introduction of various moieties at the CAI arylsulfonamide warhead. For instance, the unsubstituted derivative **48a** was a selective and potent inhibitor for the hCA XII isoform, with a *K*_I_ value of 3.7 nM. A progressive decrease in the inhibition values was obtained for **48a** on hCAs I, II, and IX, respectively (i.e., *K*_I_s of 82.1, 49.1, and 30.6 nM for hCAs I, II, and IX, respectively). Introduction of the chloro atom at the 3‐position of the phenyl moiety, as in **48b**, led to a significant reduction of the ligand affinity for the hCA I, IX, and XII isoforms between 1.6‐ and 9.1‐fold (i.e., *K*_I_s of 769.7, 48.4, and 31.1 nM for hCAs I, IX, and XII, respectively). Compound **48b** was beneficial only for isozyme II, as a 2.4‐fold inhibition enhancement was obtained (i.e., *K*_I_s of 49.1 and 20.7 nM for **48a** and **48b,** respectively). Substitution of the chloro atom in **48b** with the bulky –CF_3_ moiety afforded the high potent and selective hCA XII inhibitor **48c** (i.e., *K*_I_ of 9.4 nM). Conversely, the fluoro derivative **48d** showed a remarkable increase in the *K*_I_ values and loss of selectivity toward all of the isoforms tested (Table 1). As for the 1,4‐disubstituted diazepane derivatives **49a–d**, progressive enhancement of the inhibition potency was reported for the unsubstituted **49a** on hCAs I, II, IX, and XII, with *K*_I_ values of 315.1, 288.5, 13.8, and 5.5 nM, respectively. Introduction of the chloro atom at the 3‐position (i.e., **49b**) led to a reduction of the *K*_I_ values for hCAs I and II isoforms, whereas the opposite trend was observed for the IX and XII isozymes, although all the values were in the medium‐high nanomolar range (Table 1). Change of the chloro in **49b** with a fluoro instead to afford **49c** further enhanced the inhibition potency for hCAs I, II, and XII up to low nanomolar *K*_I_ values (i.e., *K*_I_s of 5.3, 7.2, and 7.9 nM for hCAs I, II, and XII, respectively), whereas a slight increase in the *K*_I_ value was reported for the hCA IX isoform (i.e., *K*_I_ of 284.6 nM). Fluoro disubstitution, as in **49d**, led to a decrease in the affinity of the ligand toward all hCAs considered in this study (Table 1).

Overall, the compounds synthesized in this study showed interesting in vitro activity toward the *Malassezia spp*. CAs, with *K*_I_ values in the low‐/sub‐micromolar ranges. Specifically, Mpa‐ and MreCA were preferentially inhibited in terms of their hydrase activity over the MgCA isoform.

### 2.3 Antifungal Assays on *Malassezia spp*

All synthesized compounds were tested in vitro using the microdilution assay to evaluate the minimum inhibitory concentration (MIC) on *M. pachydermatis* DSM 6172, *M. furfur* ATCC 14521, and *M. globosa* ATCC MYA 4612 reference strains. The data obtained are reported in Table 2 and were compared with the reference drug KTZ.

**Table 2:** In vitro evaluation of the compounds using the minimum inhibitory concentration (MIC) assay on *Malassezia* spp. (i.e., *M. pachydermatis* DSMZ 6172, *M. furfur* ATCC 14521, and *M. globosa* ATCC MYA 4612). The drug ketoconazole (KTZ) was used as the reference. MIC values were expressed as average MIC (µg/mL) of three experiments, with three replicates each ± standard deviation (SD).

	MIC (µg/mL ± SD) *M. pachydermatis* DSMZ 6172	MIC (µg/mL ± SD) *M. furfur* ATCC 14521	MIC (µg/mL ± SD) *M. globosa* ATCC MYA 4612
**7**	0.083 ± 0.04	4.0 ± 0	> 256 ± 0
**9a**	3.6 ± 3.25	6.0 ± 2.31	≤ 0.5 ± 0.00
**9b**	≤ 0.5 ± 0	5.0 ± 2.0	2.0 ± 0
**9c**	0.6 ± 0.25	2.5 ± 1.0	1.0 ± 0
**9d**	0.6 ± 0.25	7.0 ± 2.0	2.0 ± 0
**10a**	0.33 ± 0.14	256 ± 0	26.7 ± 9.24
**24a**	≤ 0.5 ± 0	4.0 ± 0	3.0 ± 1.15
**24b**	≤ 0.5 ± 0	4.0 ± 0	2.0 ± 0
**24c**	≤ 0.5 ± 0	10.0 ± 4.0	1.0 ± 0
**24d**	≤ 0.5 ± 0	4.0 ± 0	2.0 ± 0
**25**	1.0 ± 0	16.0 ± 0	> 256 ± 0
**26**	0.0625 ± 0	8.0 ± 0	> 256 ± 0
**28**	0.0078 ± 0	0.067 ± 0	> 256 ± 0
**35a**	0.005 ± 0.002	2.0 ± 0	128 ± 0
**35b**	0.5 ± 0.43	4.0 ± 0	> 256 ± 0
**35c**	1.0 ± 0	2.0 ± 0	128 ± 0
**36a**	0.0625 ± 0	0.25 ± 0	> 256 ± 0
**36b**	0.0625 ± 0	2.0 ± 0	64 ± 0
**36c**	0.0625 ± 0	2.0 ± 0	16 ± 0
**37a**	0.0625 ± 0	1.0 ± 0	4 ± 0
**37b**	0.0625 ± 0	4.0 ± 0	3.3 ± 1.15
**37c**	0.031 ± 0	4.0 ± 0	4.3 ± 3.5
**38**	0.013 ± 0.004	0.25 ± 0	13.3 ± 4.62
**39**	0.125 ± 0	2.0 ± 0	128 ± 0
**48a**	0.25 ± 0	8.0 ± 0	> 256 ± 0
**48b**	0.031 ± 0	4.0 ± 0	8 ± 0
**48c**	0.0625 ± 0	4.0 ± 0	5.3 ± 2.3
**48d**	0.0625 ± 0	8.0 ± 0	106.7 ± 36.9
**49a**	0.0625 ± 0	10.7 ± 4.62	13.3 ± 4.62
**49b**	0.031 ± 0	2.0 ± 0	4 ± 0
**49c**	0.031 ± 0	1.0 ± 0	> 256 ± 0
**49d**	0.05 ± 0.02	4.0 ± 0	> 256 ± 0
KTZ	0.0312 ± 0	0.25 ± 0	0.25 ± 0

As reported above, relevant data were obtained for the *M. pachydermatis* DSMZ 6172 strain, as the majority of compounds screened were highly effective growth inhibitors on this yeast, with associated MIC values below the 1.0 µg/mL threshold value (Table 2). Compound **9a** was the least potent among the entire series (i.e., MIC of 3.6 ± 3.25 µg/mL), and an almost identical trend was observed for **9b–d**, **24a–d**, and **35b,** as their MICs were between 0.5/0.6 µg/mL (Table 2). Better results were obtained for compounds **10a** (i.e., MIC 0.33 ± 0.14 µg/mL), **48a** (i.e., MIC 0.25 ± 0 µg/mL), and **39** (i.e., MIC 0.125 ± 0 µg/mL). It is noteworthy that the benzoxazolyl derivative **28** and the shortest amido **35a** were the most effective compounds in inhibiting *M. pachydermatis*, being far more effective than the reference KTZ (i.e., MICs of 0.0078 ± 0, 0.005 ± 0.002, and 0.0312 ± 0 µg/mL, respectively). Derivative **38** was also of interest, with a MIC value of 0.013 ± 0.004 µg/mL. All the remaining derivatives showed comparable potencies, with associated MIC values being between 0.0625 and 0.031 µg/mL and therefore in the same range of the reference KTZ (Table 2).

Far higher MIC concentrations were obtained when the compounds were tested on the *M. furfur* ATCC 14521 strain, as most of the values were between 1 and 4 µg/mL, and **10a** was the only ineffective derivative (i.e., MIC of 256 µg/mL). Slightly lower effectiveness was reported for derivatives **9a**, **9d**, **24c**, **25**, **26**, **48a**, **48d**, and **49a,** with MICs spanning between 5 and 16 µg/mL (Table 2). Among the compounds tested, **36a** and **38** were equipotent to the reference KTZ (Table 2), whereas **28** was 2.6‐fold less effective (i.e., MICs of 0.67 µg/mL). Derivative **23** was the most effective in the series, being 2.0‐fold more potent than KTZ (i.e., MICs of 0.125 ± 0 and 0.25 ± 0 µg/mL, respectively).

A clearer MIC trend was obtained for the *M. globosa* ATCC MYA 4612 strain. For instance, derivatives **7**, **10a**, **25**, **26**, **28**, **35b**, **36a**, **48a**, **49c**, and **49d** were ineffective, with MIC values > 256 ± 0 µg/mL (Table 2). Compounds **35a**, **35c**, and **39** were the first to show MIC effectiveness at a 128 ± 0 µg/mL concentration, followed by **48d**, **36b,** and **10a**, which showed a marked decrease to values of 106.7 ± 36.9, 64.0 ± 0, and 26.7 ± 9.24 µg/mL, respectively. Compounds **36c**, **38**, and **49a** were almost all equipotent growth inhibitors (i.e., MICs of 16.0 ± 0 and 13.3 ± 4.62 µg/mL), followed by derivative **23** (i.e., MIC 10.7 ± 4.6 µg/mL). An interesting profile was observed for the remaining compounds. For instance, **48b** was effective at 8.0 ± 0 µg/mL, whereas for **48c,** the effective concentration reduced to 5.3 ± 2.3 µg/mL. Compounds **37a–c** and **49b** were all equipotent, with MICs ranging between 3.3 ± 1.15 and 4.3 ± 3.5 µg/mL. Particularly relevant were the data obtained from **9a–d** and **24a–d,** which were all between 0.5 and 3.0 µg/mL. Compound **9a** was the most effective growth inhibitor on *M. globosa* among the entire series, with a value of 0.5 ± 0 µg/mL (Table 2). Overall, the compounds obtained in this study were less effective as *M. globosa* ATCC MYA 4612 growth inhibitors when compared with the reference drug KTZ, which showed a MIC value of 0.25 ± 0 µg/mL, thus being 13.2‐fold more potent than the best‐performing compound **37b** (i.e., MIC 3.3 ± 1.15 µg/mL).

**Figure 2:**
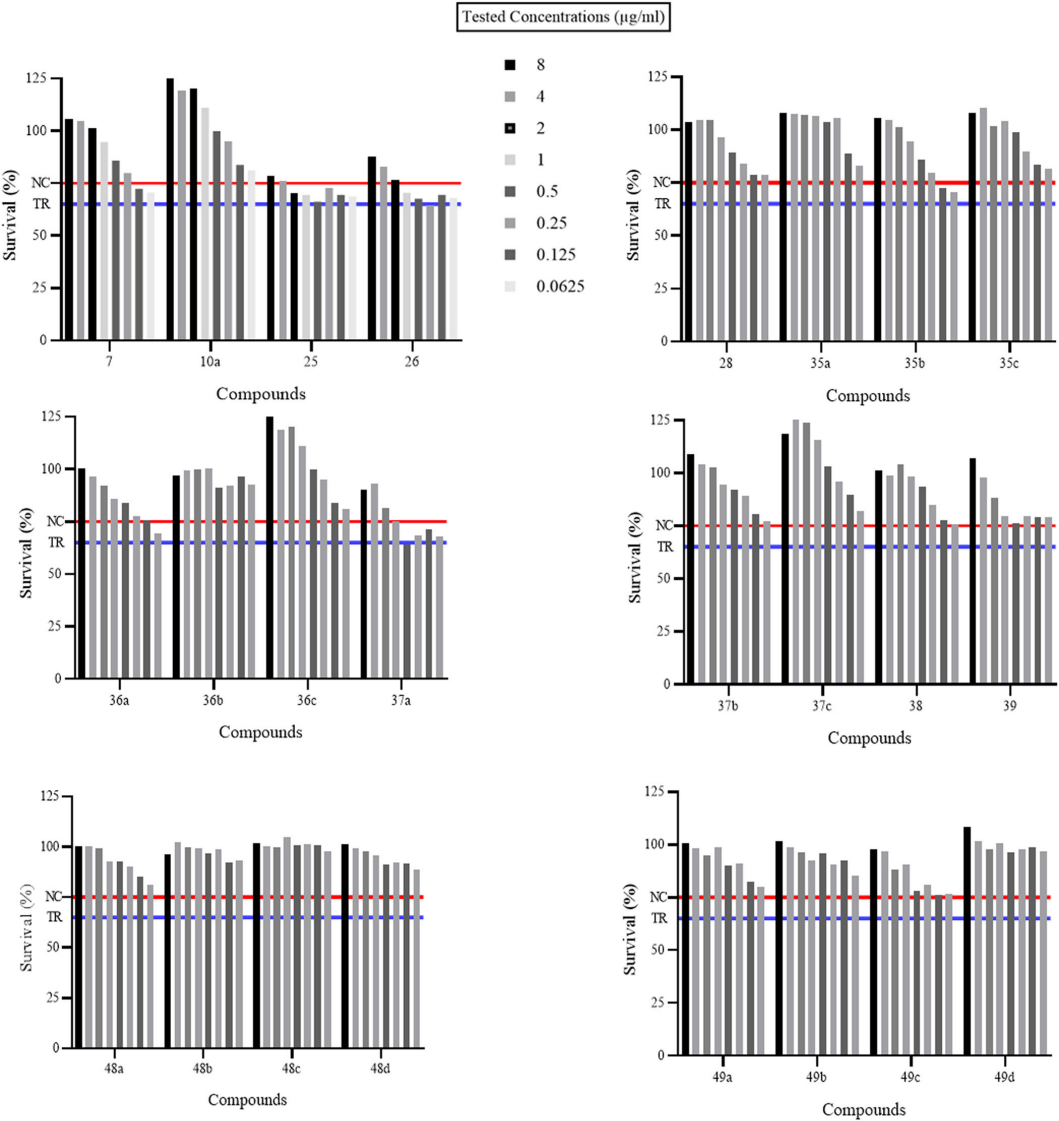
Viability assays on human keratinocytes HaCaT cells expressed as % of cell survival. NC, negative control (Y = 77%) and TR, threshold of survival (Y = 65%).

The most effective compounds were assessed for their effects on human (h) keratinocytes HaCaT cells, considering the values reported by the reference drug KTZ (Figure 2).

Overall, the cell viability of human cells was high in the presence of KTZ derivatives, and among them, the most tolerated compound was **48c**. Derivative **25** was associated with cell survival values that were consistently above the threshold limit. Higher percentages of cell survival were reported for the compounds screened at the highest concentration; in some cases, an increase in the cell population was observed when compared with the negative control with 1% DMSO. This aspect needs to be investigated in depth to assess whether the data obtained by the MTT test can be traced back to an increased cell proliferation that could be quantified through a cell counting kit (CCK‐8) [20].

Data in Figure 2, 3 show cell survival percentages closely matching those of KTZ when the compounds were tested at 0.5 µg/mL, whereas an increase in the concentration up to 16.0 µg/mL (i.e., 32‐fold higher than the effective dose against *M. pachydermatis*) led to an increase in the viability, with no marked differences for the reference KTZ. Relevant differences were observed at a 256.0 µg/mL concentration (i.e., 512‐fold higher than the MIC value), as the KTZ induced significantly low keratinocyte survival (i.e., 10.50% ± 0.36), whereas all tested compounds showed just a slight decrease, with percentages ranging from 60% to 90%. On the basis of the data analysis reported above, it is possible to state that although compounds are found to be less effective than KTZ in some cases, they are clearly highly compatible with human cells at high dosages and this endows them with high therapeutic indexes.

**Figure 3:**
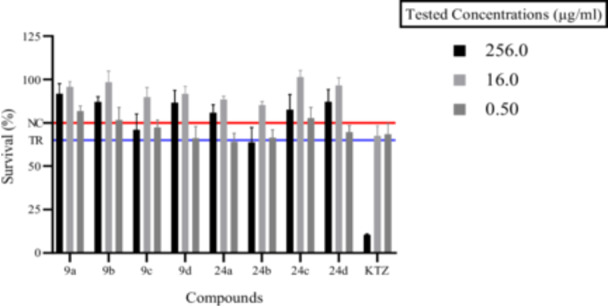
Viability assays on human keratinocytes HaCaT cells expressed as % survival ± SD % at three different concentrations (256.0, 16.0, and 0.5 µg/mL) and ketoconazole (KTZ) as the reference compound. NC, negative control (Y = 77%) and TR, threshold of survival (Y = 65%).

### 2.4 Sterol Analysis

Based on previous experiments, **9a–d**, **35a**, **38**, **49b**, and **49c** were selected to assess the ability to inhibit the *Malassezia spp*. CYP51 expressed enzyme by evaluating any disruption in the biosynthesis of ergosterol. Despite our efforts, it proved difficult to grow *Malassezia spp*. (i.e., *M. globosa* and *M. pachydermatis*), and therefore, we performed our experiments on *Candida albicans* instead by administering **9a–d**, **35a**, **38**, **49b**, **49c**, and the reference drug KTZ at the final concentrations listed in Table 3, in agreement with experimentally determined KTZ MIC values [21] and MICs for *M. globosa*.

**Table 3:** Final concentrations of compounds 9a‐d, 35a, 38, 49b, 49c, and KTZ used to treat wild‐type *Candida albicans*.

Compound	Concentration (µg/mL)
**9a**	0.06
**9b**	0.24
**9c**	0.12
**9d**	0.24
**35a**	0.48
**38**	0.48
**49b**	0.48
**49c**	0.48
KTZ	0.03

**Figure 4:**
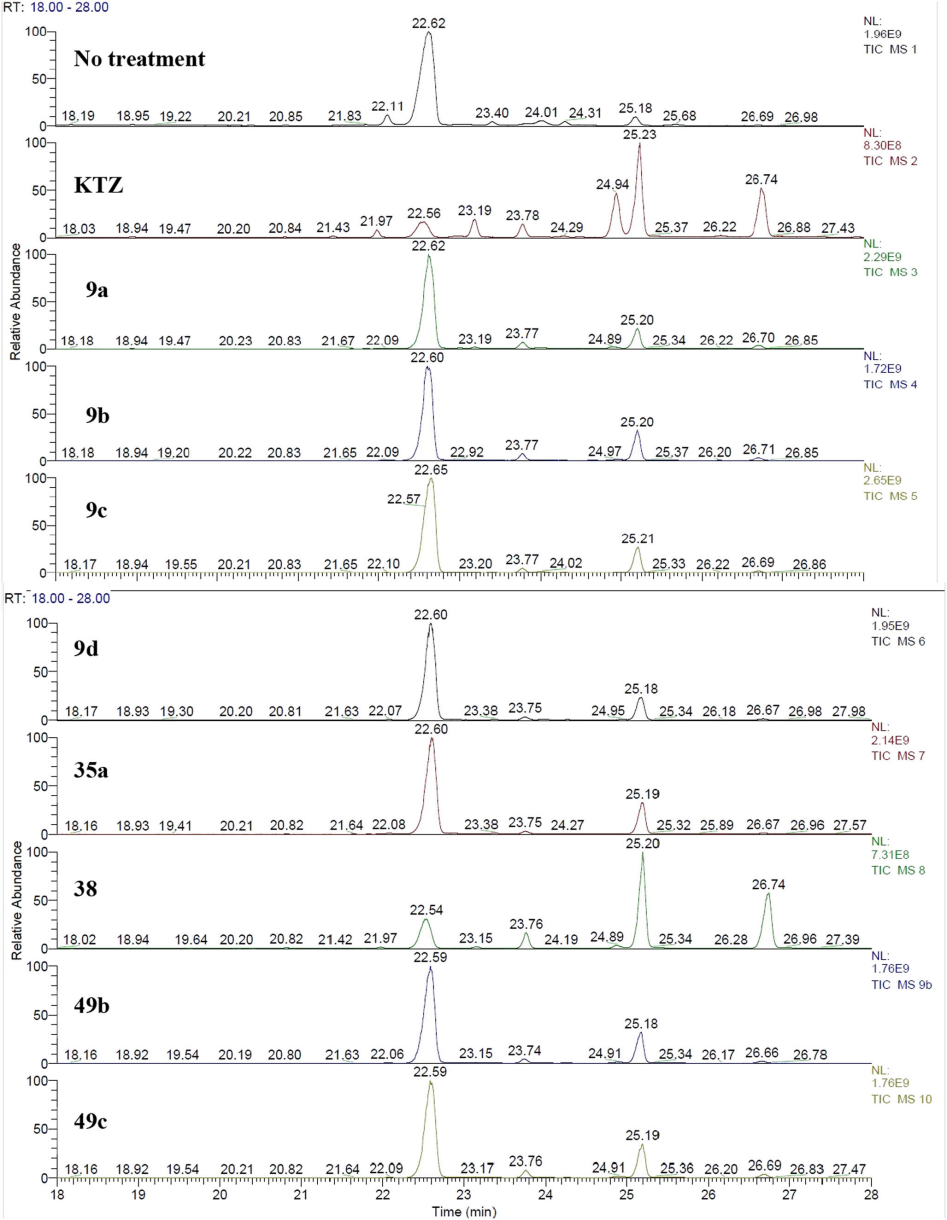
GC‐MS traces of the sterol content for each *Candida albicans* culture treated with **9a–d**, **35a**, **38**, **49b**, **49c**, and KTZ as the reference drug.

Each culture was shaken at 30°C for 48 h, followed by sterol extraction and GC‐MS identification by comparison of the main peaks (i.e., > 1% of the dominant peak) with the NIST database spectra available online (Figure 4, Supporting Information S2: Table S1) [22]. Percentage sterolic profiles (% of the total sterol extracted) were determined by calculating the peak areas determined from each identified peak in relation to the total areas of all identified peaks (Supporting Information S2: Table S2).

Overall, the GC‐MS traces showed profiles with significant accumulation of 14α‐methylated sterol derivatives in *C. albicans* wild‐type strains treated with selected compounds and thus confirmed that the KTZ moiety merged within the synthesized molecules retained its mechanism of action. Interestingly, GC‐MS traces and sterolic percentages for *C. albicans* treated with **9a–d** and **35a** were almost equal, with derivative **9b** being slightly more effective than the others (i.e., up to 1.6‐fold) in determining the accumulation of lanosterol (i.e., 16.6%). Better performances were obtained for **35a**, which, in addition to lanosterol (16.0%), induced significant accumulation of the 14α‐methylated derivative 4,4‐dimethylcholesta‐8, 24‐dien‐3‐ol (i.e., 10.8%), and was associated with the lowest ergosterol content among all (73.2%). Compounds **49b** and **49c** were identical in determining sterolic profiles and contents and were not too dissimilar to the GC‐MS trace for **9b** (Figure 2, 3). Derivative **38** was particularly effective in inhibiting the CYP51 enzyme in *C. albicans,* as ergosterol accounted for just 21.5% of the total sterol content, and it induced significant accumulation of 4,4‐dimethylcholesta‐8, 24‐dien‐3‐ol (i.e., 5.9%), lanosterol (41.6%), and 4,14‐dimethyl‐9,19‐cycloergost‐24(28)‐en‐3‐ol acetate (28.8%). It is necessary to point out that the results reported here and based on the *C. albicans* model should be considered as a general reference method that takes into account additional discrepancies among the strains, such as the metabolism of sterol.

## 3 Conclusions

Herein, we report the design and synthesis of compounds with the antifungal KTZ pharmacophoric moiety connected by means of alkyl/aryl spacers to a warhead‐directed CAI of the primary sulfonamide type. The main aim of this study is to present experimental evidence for next‐generation antifungal agents able to inhibit both yeast‐expressed 14 α‐demethylase (CYP51) and CA enzymes.

The library of compounds reported herein was developed using a rational approach based on elongation of the KTZ piperazine distal nitrogen conveniently modified with the moieties of interest. No major difficulties were encountered during the synthetic and/or the purification processes up to gram scales, thus proving the sustainability of the approach.

All final compounds showed effective inhibition of the relevant *Malassezia* spp. expressed CAs (i.e., MgCA, MpaCA, and MreCA), with experimental *K*_I_ values in the low‐sub‐micromolar range. Although hCAs were far more susceptible to inhibition (i.e., *K*_I_s in the low nanomolar range), all compounds had low cytotoxicity, with an overall threshold survival on HaCaT cells of 65% at an 8 µg/mL concentration. MIC values clearly showed that the tested compounds did not have exceptional antifungal efficacy against *Malassezia* spp. strains when compared with KTZ. Nevertheless, a remarkable reduction in cellular toxicity compared with KTZ is quite evident. The low cytotoxic profile of the compounds reported here makes them exploitable at higher concentrations and possibly also for systemic administration for the management of difficult‐to‐treat fungal infections as a replacement for KTZ. Such an application would be quite beneficial to eliminate the typical time‐ and dose‐dependent side effects of this molecule (i.e., suppression of testosterone synthesis) [23]. Data reported in this study allow us to reasonably speculate that the compounds tested are inhibitors of the 14α‐demethylase enzyme, and suppress ergosterol synthesis along with accumulation of 14α‐methylated sterol derivatives within the yeast membranes. The net effect of this process is an additional contribution to reduce the growth of *Malassezia* spp., as observed in our experiments. For clarity, our data are indicative of retention of the KTZ sterol inhibition in reference to *C. albicans*. This model, although scientifically relevant for our purposes, does not consider any differences in the metabolism of cholesterol between fungal strains.

The preferential isoform selectivity of our compounds for the fungal strain‐expressed carbonic anhydrases (CAs) over human CAs does not represent a limitation for further development, since *Malassezia spp*. are typically located on superficial cutaneous areas in humans and pets. Overall, we are confident that the compounds reported in this study are effective antifungal agents endowed with high safety profiles, which make them more appropriate for the management of infections promoted by fungal/yeast by means of topical administration.

## 4 Experimental

### 4.1 Chemistry

#### 4.1.1 General

Anhydrous solvents and all reagents were purchased from Sigma‐Aldrich, Alfa Aesar, and Fluorochem (Milan‐Italy). All reactions involving air‐ or moisture‐sensitive compounds were performed under a nitrogen atmosphere using dried glassware and syringe techniques to transfer solutions. Nuclear magnetic resonance (^1^H‐NMR, ^13^C‐NMR) spectra were recorded using a Bruker Advance III 400 MHz spectrometer in DMSO‐*d*_*6*_. Chemical shifts are reported in parts per million (ppm) and the coupling constants (*J*) are expressed in Hertz (Hz). Splitting patterns are designated as follows: s, singlet; d, doublet; t, triplet; q, quartet; m, multiplet; dd, doublet of doublets; bs, broad singlet; ap s, apparent singlet; ap d, apparent doublet; ap t, apparent triplet; and ap q, apparent quartet. The assignment of exchangeable protons (O*H* and N*H*) was confirmed by the addition of D_2_O. Analytical thin‐layer chromatography (TLC) was carried out on Merck silica gel F‐254 plates. Flash chromatography purifications were performed on Merck silica gel 60 (230–400 mesh ASTM) as the stationary phase, and methanol/dichloromethane (MeOH/DCM) or ethyl acetate/hexane (EtOAc/Hex) were used as eluents. The solvents used in Mass Spectra (MS) measurements were acetone, acetonitrile (Chromasolv grade), and 56 mQ H_2_O 18 MΩ, obtained from Millipore's Simplicity system (Milan‐Italy). The mass spectra were obtained using a Varian 1200 L triple quadrupole system (Palo Alto, USA) equipped by the Electrospray Source (ESI) operating in both positive and negative modes. Stock solutions of analytes were prepared in acetone at 1.0 mg/mL and stored at 4°C. Working solutions of each analyte were freshly prepared by diluting stock solutions in a mixture of mQ H_2_O/ACN 1/1 (v/v) up to a concentration of 1.0 µg/mL. The mass spectra of each analyte were acquired by introducing, via a syringe pump at 10 L/min, the working solution. RawQdata were collected and processed by Varian Workstation Vers. 6.8.

The InChI codes of the investigated compounds, together with some biological activity data, are provided as the Supporting Information.

#### 4.1.2 Synthesis of 1‐(4‐{[(2 *R*,4*S*)‐2‐[(1*H*‐Imidazol‐1‐yl)Methyl]‐2‐(2,4‐Dichlorophenyl)‐1,3‐Dioxolan‐4‐yl]Methoxy}Phenyl)Piperazine (2)

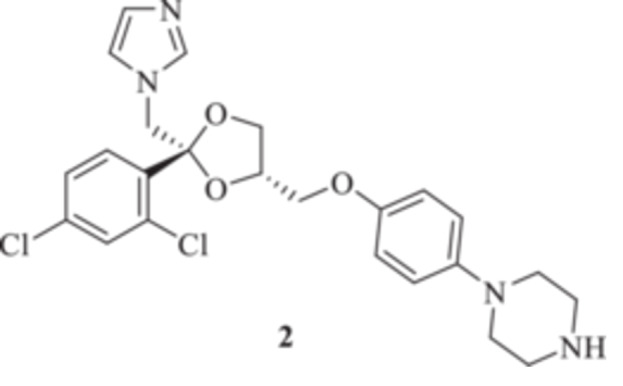

In a round‐bottom flask, KTZ **1** (2.0 g; 3.78 mmol; 1.0 eq.) was suspended in MeOH (25 mL). The reaction mixture was stirred at reflux temperature until complete dissolution. Then, aqueous NaOH 20% *w/v* (5–6 mL) was added. The reaction mixture was stirred at reflux temperature for 12 h. A control via TLC was performed to ensure complete consumption of the starting materials. The reaction was cooled to room temperature, diluted with H_2_O, and a white precipitate was formed, filtered off under vacuum, washed with H_2_O, and dried in air. No further purification was needed. White solid 91% yield; ^1^H NMR (DMSO‐*d*_*6*_, 400 MHz): 7.73 (1H, d, *J* = 2.08 Hz), 7.60 (1H, d, *J* = 8.44 Hz), 7.51 (1H, s), 7.49 (1H, dd, *J* = 8.54 Hz; *J* = 2.15 Hz), 7.05 (1H, s), 6.89 (2H, d, *J* = 9.12 Hz), 6.85 (1H, s), 6.81 (2H, d, *J* = 9.12 Hz), 4.56 (2H, q, *J* = 11.25 Hz), 4.37 (1H, m, N*H* exchange with D_2_O), 3.90 (1H, t, *J* = 5.01 Hz), 3.67 (2H, m), 3.54 (1H, dd, *J* = 10.20 Hz; *J* = 5.09 Hz), 2.95 (4H, t, *J* = 4.80 Hz), 2.85 (4H, t, *J* = 4.78 Hz), 2.21 (1H, s); ^13^C NMR (CDCl_3_, 100 MHz): 152.3, 146.8, 138.8, 135.9, 134.7, 133.0, 131.3, 129.5, 128.6, 127.2, 121.1, 118.1, 115.3, 108.0, 74.9, 67.8, 67.6, 51.8, 51.3, 46.3; ESI‐HRMS (*m*/*z*) calculated for [M+H]^+^ ion species C_24_H_26_Cl_2_N_4_O_3_ 488.1382, found 488.1386; Elemental analysis, calculated: C, 58.90; H, 5.36; N, 11.45; found: C, 58.92; H, 5.39; N, 11.43.

#### 4.1.3 General Procedure for the Synthesis of Ureido Derivatives **7**, **9a–d**, and **10a,b**

1‐(4‐{[(2 *R*,4*S*)‐2‐[(1*H*‐Imidazol‐1‐yl)methyl]‐2‐(2,4‐dichlorophenyl)‐1,3‐dioxolan‐4‐yl]methoxy}phenyl)piperazine (**2**) (1.0 eq.) and the appropriate carbamate (**3–6**) (1.0 eq.) were dissolved in acetonitrile (4 mL). The reaction mixture was stirred at reflux temperature for 12 h. A control via TLC was performed to ensure complete consumption of the starting materials. The reaction was quenched with H_2_O and the precipitate was filtered off under vacuum, washed with Et_2_O and H_2_O, and then dried in air. Purification via silica gel flash chromatography was performed to afford the pure products.

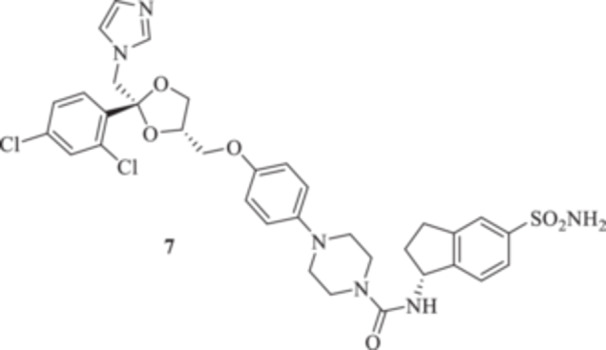

*N*‐[(*R*)‐5‐Sulfamoyl‐2,3‐dihydro‐1*H*‐inden‐1‐yl]‐4‐{4‐[((2 *R*,4*S*)‐2‐[(1*H*‐imidazol‐1‐yl)methyl]‐2‐(2,4‐dichlorophenyl)‐1,3‐dioxolan‐4‐yl]methoxy}phenyl}piperazine‐1‐carboxamide (**7**): White solid 64% yield; ^1^H NMR (DMSO‐*d*_*6*_, 400 MHz): 7.73 (1H, s, N*H*, exchange with D_2_O), 7.69 (2H, s), 7.60 (1H, d, *J* = 8.44 Hz), 7.52 (1H, s), 7.50 (1H, d, *J* = 9.00 Hz), 7.43 (1H, d, *J* = 8.36 Hz), 7.35 (2H, bs, SO_2_N*H*_2_, exchange with D_2_O), 7.06 (2H, d, *J* = 5.52 Hz), 6.96 (2H, d, *J* = 8.80 Hz), 6.85 (2H, d, *J* = 7.48 Hz), 6.82 (1H, s), 5.33 (1H, q, *J* = 8.26 Hz), 4.57 (2H, q, *J* = 11.32 Hz), 4.38 (1H, m), 3.90 (1H, t, *J* = 7.50 Hz), 3.68 (2H, m), 3.54 (5H, m), 3.03 (4H, m), 2.99 (1H, m), 2.86 (1H, m), 2.45 (1H, m), 1.96 (1H, m); ^13^C NMR (DMSO‐*d*_*6*_, 100 MHz): 158.3, 152.9, 147.8, 147.2, 146.6, 143.5, 139.4, 136.1, 135.4, 133.3, 131.5, 131.0, 128.5, 128.2, 125.7, 125.6, 122.2, 118.7, 116.0, 108.6, 75.5, 68.7, 67.6, 65.8, 56.0, 51.5, 50.6, 44.5, 34.2, 30.5; ESI‐HRMS (*m*/*z*) calculated for [M+H]^+^ ion species C_34_H_36_Cl_2_N_6_O_6_S 727.6580, found 727.6584; Elemental analysis, calculated: C, 56.12; H, 4.99; N, 11.55; found: C, 56.16; H, 5.03; N, 11.51.

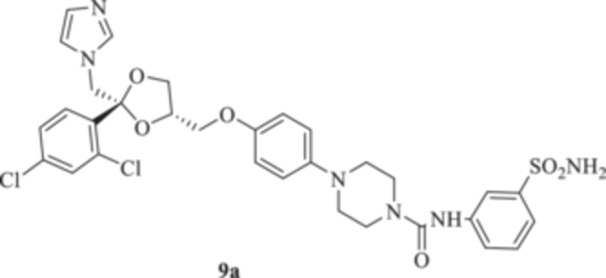

*N*‐(3‐Sulfamoylphenyl)‐4‐{4‐[((2 *R*,4*S*)‐2‐[(1*H*‐imidazol‐1‐yl)methyl]‐2‐(2,4‐dichlorophenyl)‐1,3‐dioxolan‐4‐yl)methoxy]phenyl}piperazine‐1‐carboxamide (**9a**): White solid 48% yield; ^1^H NMR (400 MHz, DMSO‐*d*_*6*_) *δ*(ppm): 8.99 (1H, s, N*H*, exchange with D_2_O), 8.08 (1H, s), 7.73 (2H, s), 7.61 (1H, d, *J* = 8.28 Hz), 7.52 (1H, s), 7.46 (3H, m), 7.35 (2H, bs, SO_2_N*H*_2_, exchange with D_2_O), 7.06 (1H, s), 6.99 (2H, d, *J* = 8.36 Hz), 6.84 (3H, m), 4.57 (2H, q, *J* = 11.23 Hz), 4.38 (1H, m), 3.90 (1H, t, *J* = 7.14 Hz), 3.65 (6H, m), 3.55 (1H, m), 3.08 (4H, m); ^13^C NMR (100 MHz, DMSO‐*d*_*6*_) *δ*(ppm): 155.6, 153.0, 146.5, 145.2, 141.9, 136.1, 135.4, 133.3, 131.6, 131.0, 129.8, 128.5, 128.2 123.1, 122.0, 119.6, 118.7, 117.3, 116.0, 108.6, 75.5, 68.7, 67.6, 51.5, 50.6, 44.7; ESI‐HRMS (*m*/*z*) calculated for [M+H]^+^ ion species C_31_H_32_Cl_2_N_6_O_6_S 687.5930, found 687.5934; Elemental analysis, calculated: C, 54.15; H, 4.69; N, 12.22; found: C, 54.17; H, 4.72; N, 12.25

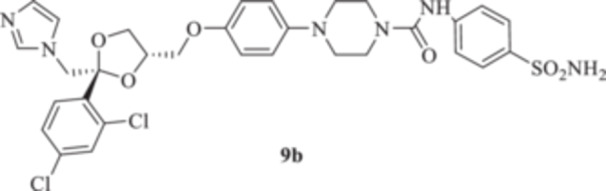

*N*‐(4‐Sulfamoylphenyl)‐4‐{4‐[((2 *R*,4*S*)‐2‐[(1*H*‐imidazol‐1‐yl)methyl]‐2‐(2,4‐dichlorophenyl)‐1,3‐dioxolan‐4‐yl)methoxy]phenyl}piperazine‐1‐carboxamide(**9b**): White solid 53% yield; ^1^H NMR (400 MHz, DMSO‐*d*_*6*_) *δ*(ppm): 9.02 (1H, s, N*H*, exchange with D_2_O), 7.72 (2H, m), 7.68 (2H, d, *J* = 8.88 Hz), 7.61 (1H, d, *J* = 8.48 Hz), 7.52 (1H, s), 7.50 (1H, dd, *J* = 8.55 Hz; *J* = 1.99 Hz), 7.21 (2H, bs, SO_2_N*H*_2_, exchange with D_2_O), 7.05 (1H, s), 6.99 (2H, d, *J* = 8.96 Hz), 6.86 (2H, s), 6.83 (1H, s), 6.78 (1H, d, *J* = 8.32 Hz), 4.57 (2H, q, *J* = 11.20 Hz), 4.38 (1H, m), 3.90 (1H, t, *J* = 7.50 Hz), 3.68 (6H, m), 3.56 (1H, dd, *J* = 10.22 Hz; *J* = 5.27 Hz), 3.09 (4H, m); ^13^C NMR (100 MHz, DMSO‐*d*_*6*_) *δ*(ppm): 155.5, 153.0, 146.5, 136.2, 135.4, 133.3, 131.6, 131.0, 130.3, 128.2, 127.3, 119.7 119.5, 118.8, 116.0, 108.7, 75.5, 68.7, 67.7, 51.5, 50.6, 44.7; ESI‐HRMS (*m*/*z*) calculated for [M+H]^+^ ion species C_31_H_32_Cl_2_N_6_O_6_S 687.5930, found 687.5936; Elemental analysis, calculated: C, 54.15; H, 4.69; N, 12.22; found: C, 54.11; H, 4.67; N, 12.20.

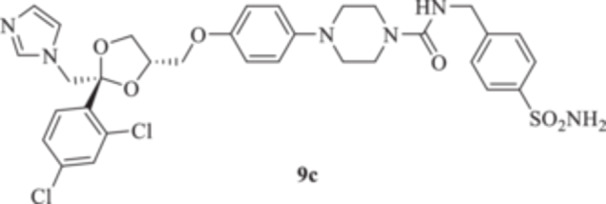

*N*‐[(4‐Sulfamoylphenyl)methyl]‐4‐{4‐[((2 *R*,4*S*)‐2‐[(1*H*‐imidazol‐1‐yl)methyl]‐2‐(2,4‐dichlorophenyl)‐1,3‐dioxolan‐4‐yl)methoxy]phenyl}piperazine‐1‐carboxamide (**9c**): White solid 44% yield; ^1^H NMR (DMSO‐*d*_*6*_, 400 MHz): 7.79 (2H, d, *J* = 8.24 Hz), 7.73 (1H, d, *J* = 2.00 Hz), 7.61 (1H, d, *J* = 8.44 Hz), 7.52 (1H, s, N*H*, exchange with D_2_O), 7.50 (1H, d, *J* = 8.58 Hz; *J* = 2.07 Hz), 7.47 (2H, d, *J* = 8.24 Hz), 7.34 (2H, bs, SO_2_N*H*_2_, exchange with D_2_O), 7.32 (1H, m), 7.06 (1H, s), 6.96 (2H, d, *J* = 9.00 Hz), 6.86 (1H, s), 6.83 (2H, d, *J* = 8.96 Hz), 4.57 (2H, q, *J* = 11.22 Hz), 4.38 (1H, m), 4.34 (2H, d, *J* = 5.40 Hz), 3.90 (1H, t, *J* = 7.50 Hz), 3.68 (2H, m), 3.55 (1H, t, *J* = 5.10 Hz), 3.51 (4H, m), 3.02 (4H, m); ^13^C NMR (DMSO‐*d*_*6*_, 100 MHz): 158.2, 152.9, 146.6, 146.1, 143.2, 131.5, 131.0, 130.2, 128.2, 126.4, 119.7, 118.7, 116.1, 116.0, 108.6, 75.5, 68.7, 67.6, 65.8, 51.5, 50.5, 44.4; ESI‐HRMS (*m*/*z*) calculated for [M+H]^+^ ion species C_32_H_34_Cl_2_N_6_O_6_S 701.6200, found 701.6215; Elemental analysis, calculated: C, 54.78; H, 4.88; N, 11.98; found: C, 54.80; H, 4.93; N, 12.01.

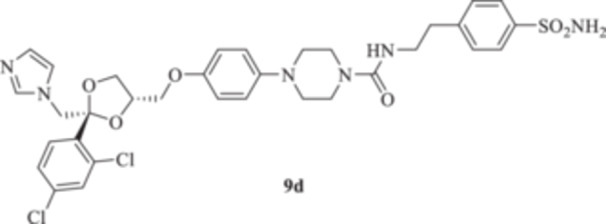

*N*‐[2‐(4‐Sulfamoylphenyl)ethyl]‐4‐{4‐[((2 *R*,4*S*)‐2‐[(1*H*‐imidazol‐1‐yl)methyl]‐2‐(2,4‐dichlorophenyl)‐1,3‐dioxolan‐4‐yl)methoxy]phenyl}piperazine‐1‐carboxamide (**9 d**): White solid 58% yield; ^1^H NMR (DMSO‐*d*_*6*_, 400 MHz): 7.78 (2H, t, *J* = 8.09 Hz), 7.72 (1H, d, *J* = 1.82 Hz), 7.61 (1H, d, *J* = 8.47 Hz), 7.51 (1H, s, N*H*, exchange with D_2_O), 7.49 (1H, dd, *J* = 8.53 Hz; *J* = 1.85 Hz), 7.42 (2H, d, *J* = 8.12 Hz), 7.32 (2H, bs, SO_2_N*H*_2_, exchange with D_2_O), 7.05 (1H, s), 6.95 (2H, d, *J* = 8.97 Hz), 6.84 (3H, t, *J* = 7.80 Hz), 6.77 (1H, d, *J* = 5.76 Hz), 4.57 (2H, q, *J* = 11.14 Hz), 4.38 (1H, m), 3.90 (1H, t, *J* = 7.50 Hz), 3.68 (2H, m), 3.56 (1H, dd, *J* = 9.82 Hz; *J* = 4.92 Hz), 3.45 (4H, m), 3.31 (2H, q, *J* = 6.56 Hz), 2.99 (4H, m), 2.85 (2H, t, *J* = 7.26 Hz); ^13^C NMR (DMSO‐*d*_*6*_, 100 MHz): 158.2, 152.9, 146.6, 145.0, 142.8, 136.1, 135.4, 131.5, 130.3, 130.0, 128.2, 126.6, 119.7, 118.7, 116.1, 116.0, 108.6, 75.5, 68.7, 67.6, 51.5, 50.6, 44.4, 42.4, 36.6; ESI‐HRMS (*m*/*z*) calculated for [M+H]^+^ ion species C_33_H_36_Cl_2_N_6_O_6_S 715.6470, found 715.6473; Elemental analysis, calculated: C, 55.39; H, 5.07; N, 11.74; found: C, 55.41; H, 5.03; N, 11.72

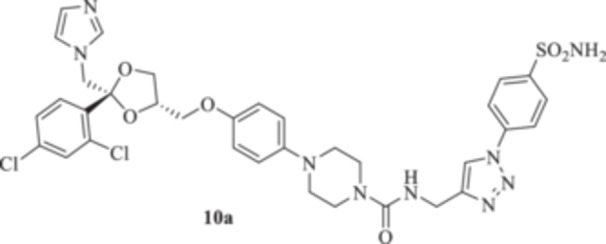

*N*‐{[(1‐(4‐Sulfamoylphenyl)‐1*H*‐1,2,3‐triazol‐4‐yl)methyl]}‐4‐{4‐((2 *R*,4*S*) − 2 − [(1*H*−imidazol−1−yl)methyl]−2 − (2,4−dichlorophenyl)−1,3−dioxolan−4−yl)methoxyphenyl}piperazine‐1‐carboxamide (**10a**): Light brown solid 49% yield; ^1^H NMR (DMSO‐*d*_*6*_, 400 MHz): 8.73 (1H, s, N*H*, exchange with D_2_O), 8.18 (2H, d, *J* = 8.44 Hz), 8.04 (2H, d, *J* = 8.44 Hz), 7.73 (1H, s), 7.61 (1H, d, *J* = 8.40 Hz), 7.57 (2H, bs, SO_2_N*H*_2_, exchange with D_2_O), 7.51 (1H, s), 7.48 (1H, m), 7.29 (1H, t, *J* = 4.95 Hz), 7.05 (1H, s), 6.96 (2H, d, *J* = 8.72 Hz), 6.85 (1H, s), 6.82 (2H, d, *J* = 8.48 Hz), 4.57 (2H, q, *J* = 11.27 Hz), 4.42 (2H, d, *J* = 4.88 Hz), 4.37 (1H, t, *J* = 5.52 Hz), 3.90 (1H, t, *J* = 7.40 Hz), 3.67 (2H, m), 3.54 (1H, t, *J* = 5.26 Hz), 3.50 (4H, m), 3.02 (4H, m); ^13^C NMR (DMSO‐*d*_*6*_, 100 MHz): 161.1, 152.6, 146.7, 140.5, 139.5, 136.1, 135.4, 133.3, 132.8, 131.5, 131.0, 130.4, 128.5, 122.0, 121.0, 119.2, 118.0, 117.0, 116.0, 108.6, 75.5, 68.7, 67.6, 51.5, 50.6, 44.7, 43.2, 35.7; ESI‐HRMS (*m*/*z*) calculated for [M+H]^+^ ion species C_34_H_35_Cl_2_N_9_O_6_S 768.6710, found 768.6718; Elemental analysis, calculated: C, 53.13; H, 4.59; N, 16.40; found: C, 53.15; H, 4.62; N, 16.37.

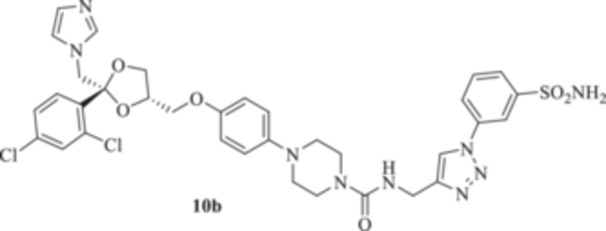

*N*‐[(1‐(3‐Sulfamoylphenyl)‐1*H*‐1,2,3‐triazol‐4‐yl)methyl]‐4‐{4‐[((2 *R*,4*S*)‐2‐[(1*H*‐imidazol‐1‐yl)methyl]‐2‐(2,4‐dichlorophenyl)‐1,3‐dioxolan‐4‐yl)methoxy]phenyl}piperazine‐1‐carboxamide (**10b**): Light brown solid 14% yield; ^1^H NMR (DMSO‐*d*_*6*_, 400 MHz): 8.71 (1H, s, N*H*, exchange with D_2_O), 8.41 (1H, s), 8.19 (1H, d, *J* = 7.96 Hz), 7.93 (1H, d, *J* = 7.84 Hz), 7.83 (1H, t, *J* = 7.96 Hz), 7.73 (1H, d, *J* = 1.88 Hz), 7.62 (2H, bs, SO_2_N*H*_2_, exchange with D_2_O), 7.59 (1H, s), 7.51 (1H, s), 7.49 (1H, dd, *J* = 8.72 Hz; *J* = 1.92 Hz), 7.29 (1H, t, *J* = 5.24 Hz), 7.05 (1H, s), 6.95 (2H, d, *J* = 9.00 Hz), 6.83 (3H, t, *J* = 8.52 Hz), 4.57 (2H, q, *J* = 11.40 Hz), 4.43 (2H, d, *J* = 5.08 Hz), 4.37 (1H, t, *J* = 5.72 Hz), 3.90 (1H, t, *J* = 7.48 Hz), 3.67 (2H, m), 3.55 (1H, t, *J* = 5.14 Hz), 3.50 (4H, m), 3.02 (4H, m); ^13^C NMR (DMSO‐*d*_*6*_, 100 MHz): 161.1, 152.6, 146.7, 140.5, 139.5, 136.1, 135.4, 133.3, 132.8, 131.5, 131.0, 130.4, 128.5, 128.2, 122.7, 122.0, 121.0, 119.2, 118.0, 117.0, 116.0, 108.6, 75.5, 68.7, 67.6, 51.5, 50.6, 44.7, 43.2, 35.7; ESI‐HRMS (*m*/*z*) calculated for [M+H]^+^ ion species C_34_H_35_Cl_2_N_9_O_6_S 768.6710, found 768.6706; Elemental analysis, calculated: C, 53.13; H, 4.59; N, 16.40; found: C, 53.10; H, 4.57; N, 16.42.

#### 4.1.4 General Procedure for the Synthesis of Thioureido Derivatives **24a–d**, **25**, and **26**

1‐(4‐{[(2 *R*,4*S*)‐2‐[(1*H*‐Imidazol‐1‐yl)methyl]‐2‐(2,4‐dichlorophenyl)‐1,3‐dioxolan‐4‐yl]methoxy}phenyl)piperazine (**2**) (1.0 eq.) and the appropriate isothiocyanate (**19–22**) (1.0 eq.) were dissolved in acetonitrile (4 mL). The reaction mixture was stirred at room temperature for 12 h. A control via TLC was performed to ensure complete consumption of the starting materials. The reaction was quenched with H_2_O, and the precipitate was filtered off under vacuum, washed with Et_2_O and H_2_O, and then dried in air. Purification via silica gel flash chromatography was performed to afford the pure products.

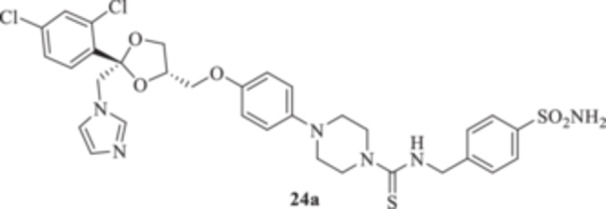

*N*‐[(4‐Sulfamoylphenyl)methyl]‐4‐{4‐[((2 *R*,4*S*)‐2‐[(1*H*‐imidazol‐1‐yl)methyl]‐2‐(2,4‐dichlorophenyl)‐1,3‐dioxolan‐4‐yl)methoxy]phenyl}piperazine‐1‐carbothioamide (**24a**): White solid 77% yield; ^1^H NMR (400 MHz, DMSO‐*d*_*6*_) *δ*(ppm): 8.45 (1H, t, N*H*, exchange with D_2_O, *J* = 5.52 Hz), 7.79 (2H, d, *J* = 8.28 Hz), 7.72 (1H, d, *J* = 2.08 Hz), 7.60 (1H, d, *J* = 8.48 Hz), 7.52 (1H, s), 7.50 (3H, m), 7.33 (2H, bs, SO_2_N*H*_2_, exchange with D_2_O), 7.05 (1H, s), 6.96 (2H, d, *J* = 9.12 Hz), 6.85 (2H, d, *J* = 1.92 Hz), 6.82 (1H, s), 4.89 (2H, d, *J* = 5.28 Hz), 4.57 (2H, q, *J* = 11.16 Hz), 4.37 (1H, m), 4.00 (4H, m), 3.90 (1H, t, *J* = 5.01 Hz), 3.68 (2H, m), 3.55 (1H, dd, *J* = 10.23 Hz; *J* = 5.24 Hz), 3.11 (4H, m); ^13^C NMR (100 MHz, DMSO‐*d*_*6*_) *δ*(ppm): 182.2, 153.0, 146.1, 144.8, 139.4, 131.5, 131.0, 129.3, 129.1, 128.5, 128.2, 122.6, 122.0, 118.5, 116.1, 108.6, 68.7, 67.6, 65.8, 51.5, 50.2, 48.9; ESI‐HRMS (*m*/*z*) calculated for [M+H]^+^ ion species C_32_H_34_Cl_2_N_6_O_5_S_2_ 717.6810, found 717.6816; Elemental analysis, calculated: C, 53.55; H, 4.78; N, 11.71; found: C, 53.52; H, 4.80; N, 11.74.

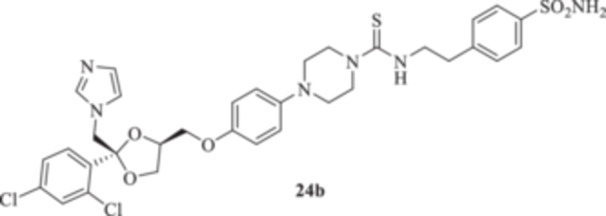

*N*‐(2‐(4‐Sulfamoylphenyl)ethyl)‐4‐{4‐[((2 *R*,4*S*)‐2‐((1*H*‐imidazol‐1‐yl)methyl)‐2‐(2,4‐dichlorophenyl)‐1,3‐dioxolan‐4‐yl)methoxy]phenyl}piperazine‐1‐carbothioamide (**24b**): White solid 80% yield; ^1^H NMR (400 MHz, DMSO‐*d*_*6*_) *δ*(ppm): 7.96 (1H, t, N*H*, exchange with D_2_O, *J* = 5.24 Hz), 7.79 (2H, d, *J* = 8.32 Hz), 7.73 (1H, d, *J* = 2.12 Hz), 7.61 (1H, d, *J* = 8.44 Hz), 7.52 (1H, s), 7.50 (1H, dd, *J* = 8.47 Hz; *J* = 2.10 Hz), 7.45 (2H, d, *J* = 8.32 Hz), 7.34 (2H, bs, SO_2_N*H*_2_, exchange with D_2_O), 7.05 (1H, s), 6.96 (2H, d, *J* = 9.12 Hz), 6.85 (2H, d, *J* = 4.40 Hz), 6.82 (1H, s), 4.57 (2H, q, *J* = 11.24 Hz), 4.38 (1H, m), 3.93 (4H, m), 3.89 (1H, m), 3.76 (2H, m), 3.68 (2H, m), 3.55 (1H, dd, *J* = 10.23 Hz; *J* = 5.25 Hz), 3.07 (4H, m), 2.99 (2H, t, *J* = 7.46 Hz); ^13^C NMR (100 MHz, DMSO‐*d*_*6*_) *δ*(ppm): 182.1, 153.0, 139.5, 136.2, 135.4, 135.4, 133.3, 131.6, 131.0, 128.6, 128.2, 126.7, 124.9, 122.1, 118.5, 108.6, 75.5, 68.7, 67.6, 51.5, 50.2, 49.1, 2.1; ESI‐HRMS (*m*/*z*) calculated for [M+H]^+^ ion species C_33_H_36_Cl_2_N_6_O_5_S_2_ 731.7080, found 731.7067; Elemental analysis, calculated: C, 54.17; H, 4.96; N, 11.49; found: C, 54.15; H, 4.99; N, 11.46.

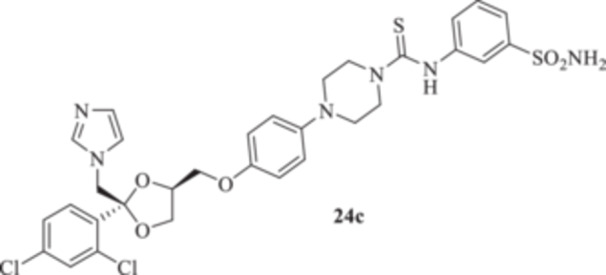

*N*‐(3‐Sulfamoylphenyl)‐4‐{4‐[((2 *R*,4*S*)‐2‐[(1*H*‐imidazol‐1‐yl)methyl]‐2‐(2,4‐dichlorophenyl)‐1,3‐dioxolan‐4‐yl)methoxy]phenyl}piperazine‐1‐carbothioamide (**24c**): White solid 91% yield; ^1^H NMR (400 MHz, DMSO‐*d*_*6*_) *δ*(ppm): 9.68 (1H, s, N*H*, exchange with D_2_O), 7.81 (1H, t, *J* = 1.80 Hz), 7.73 (1H, d, *J* = 2.11 Hz), 7.62 (1H, s), 7.60 (1H, m), 7.57 (1H, t, *J* = 1.38 Hz), 7.52 (2H, m), 7.50 (1H, dd, *J* = 8.62 Hz; *J* = 2.28 Hz), 7.41 (2H, bs, SO_2_N*H*_2_, exchange with D_2_O), 7.06 (1H, s), 6.99 (2H, d, *J* = 9.12 Hz), 6.86 (2H, s), 6.84 (1H, s), 4.57 (2H, q, *J* = 11.23 Hz), 4.38 (1H, m), 4.10 (4H, m), 3.90 (1H, t, *J* = 5.00 Hz), 3.69 (2H, m), 3.56 (1H, dd, *J* = 10.16 Hz; *J* = 5.18 Hz), 3.17 (4H, m); ^13^C NMR (100 MHz, DMSO‐*d*_*6*_) *δ*(ppm): 182.8, 152.9, 146.2, 144.8, 143.3, 133.3, 131.5, 131.0, 128.3, 128.2, 126.4, 118.4, 116.0, 108.6, 75.5, 68.7, 67.6, 51.5, 50.2, 48.9, 48.2; ESI‐HRMS (*m*/*z*) calculated for [M+H]^+^ ion species C_31_H_32_Cl_2_N_6_O_5_S_2_ 703.6540, found 703.6547; Elemental analysis, calculated: C, 52.92; H, 4.58; N, 11.94; found: C, 52.95; H, 4.61; N, 11.91.

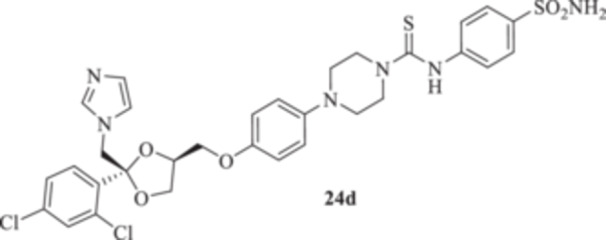

*N*‐(4‐Sulfamoylphenyl)‐4‐{4‐[((2 *R*,4*S*)‐2‐[(1*H*‐imidazol‐1‐yl)methyl]‐2‐(2,4‐dichlorophenyl)‐1,3‐dioxolan‐4‐yl)methoxy]phenyl}piperazine‐1‐carbothioamide (**24d**): White solid 59% yield; ^1^H NMR (400 MHz, DMSO‐*d*_*6*_) *δ*(ppm): 9.69 (1H, s, N*H*, exchange with D_2_O), 7.76 (2H, d, *J* = 8.64 Hz), 7.73 (1H, s), 7.61 (1H, d, *J* = 8.40 Hz), 7.54 (1H, m), 7.52 (2H, m), 7.49 (1H, m), 7.32 (2H, bs, SO_2_N*H*_2_, exchange with D_2_O), 7.06 (1H, s), 6.99 (2H, d, *J* = 8.84 Hz), 6.86 (2H, m), 6.84 (1H, m), 4.57 (2H, q, *J* = 11.22 Hz), 4.38 (1H, m), 4.09 (4H, m), 3.90 (1H, t, *J* = 7.48 Hz), 3.68 (2H, m), 3.56 (1H, m), 3.17 (4H, m); ^13^C NMR (100 MHz, DMSO‐*d*_*6*_) *δ*(ppm): 182.2, 152.9, 146.2, 144.7, 143.0, 136.1, 135.4, 133.3, 131.5, 131.0, 130.0, 128.2, 126.7, 118.5, 116.0, 108.6, 75.5, 68.7, 67.6, 65.8, 50.1, 48.0, 47.3, 35.4; ESI‐HRMS (*m*/*z*) calculated for [M+H]^+^ ion species C_31_H_32_Cl_2_N_6_O_5_S_2_ 703.6540, found 703.6536; Elemental analysis, calculated: C, 52.92; H, 4.58; N, 11.94; found: C, 52.90; H, 4.57; N, 11.96.

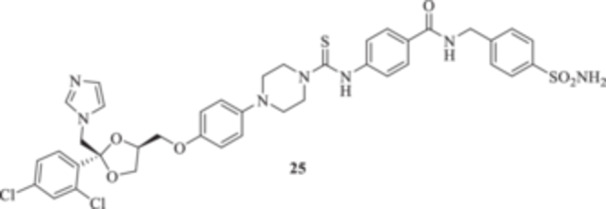

4‐[4‐(4‐(((2 *R*,4*S*)‐2‐((1*H*‐Imidazol‐1‐yl)methyl)‐2‐(2,4‐dichlorophenyl)‐1,3‐dioxolan‐4‐yl)methoxy)phenyl)piperazine‐1‐carbothioamido]‐*N*‐[(4‐sulfamoylphenyl)methyl]benzamide (**25**): Light yellow solid 66% yield; ^1^H NMR (DMSO‐*d*_*6*_, 400 MHz): 9.62 (1H, s, N*H*, exchange with D_2_O), 9.08 (1H, t, *J* = 5.84 Hz), 7.87 (2H, d, *J* = 8.48 Hz), 7.82 (2H, d, *J* = 8.16 Hz), 7.72 (1H, d, *J* = 1.68 Hz), 7.61 (1H, d, *J* = 8.44 Hz), 7.54 (1H, s), 7.51 (2H, m), 7.47 (3H, ap d, *J* = 8.24 Hz), 7.34 (2H, bs, SO_2_N*H*_2_, exchange with D_2_O), 7.05 (1H, s), 6.98 (2H, d, *J* = 8.92 Hz), 6.86 (2H, s), 6.84 (1H, s), 4.57 (4H, q, *J* = 11.02 Hz), 4.38 (1H, m), 4.09 (4H, m), 3.91 (1H, t, *J* = 7.40 Hz), 3.69 (2H, ap q, *J* = 4.77 Hz), 3.58 (1H, dd, *J* = 10.12 Hz; *J* = 5.11 Hz), 3.17 (4H, m); ^13^C NMR (DMSO‐*d*_*6*_, 100 MHz): 182.1, 166.9, 153.0, 146.1, 144.8, 143.5, 139.5, 139.4, 136.1, 135.4, 133.3, 131.6, 131.0, 130.2, 128.5, 128.4, 128.2, 126.6, 124.6, 122.0, 118.5, 116.0, 108.7, 75.5, 68.7, 67.6, 51.5, 50.2, 49.0, 43.2, 31.6; ESI‐HRMS (*m*/*z*) calculated for [M+H]^+^ ion species C_39_H_39_Cl_2_N_7_O_6_S_2_ 836.8040, found 836.8045; Elemental analysis, calculated: C, 55.98; H, 4.70; N, 11.72; found: C, 56.02; H, 4.73; N, 11.68.

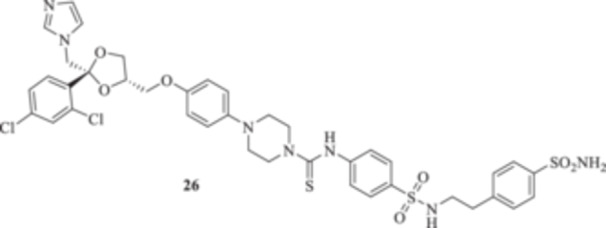

*N*‐[4‐[(*N*‐(4‐Sulfamoylphenethyl)sulfamoyl]phenyl]‐4‐{4‐[((2 *R*,4*S*)‐2‐[(1*H*‐imidazol‐1‐yl)methyl]‐2‐(2,4‐dichlorophenyl)‐1,3‐dioxolan‐4‐yl)methoxy]phenyl}piperazine‐1‐carbothioamide (**26**): Light yellow solid 64% yield; ^1^H NMR (DMSO‐*d*_*6*_, 400 MHz): 9.72 (1H, s, N*H*, exchange with D_2_O), 7.77 (1H, s), 7.75 (2H, d, *J* = 5.12 Hz), 7.73 (2H, t, *J* = 2.44 Hz), 7.67 (1H, t, *J* = 5.74 Hz), 7.60 (3H, m), 7.52 (1H, s), 7.50 (1H, dd, *J* = 8.47 Hz; *J* = 2.09 Hz), 7.41 (1H, s), 7.39 (1H, s), 7.33 (2H, bs, SO_2_N*H*_2_, exchange with D_2_O), 7.05 (1H, s), 6.98 (2H, d, *J* = 9.08 Hz), 6.86 (2H, s), 6.84 (1H, s), 4.57 (2H, q, *J* = 11.12 Hz), 4.38 (1H, m), 4.10 (4H, m), 3.91 (1H, t, *J* = 5.03 Hz), 3.69 (2H, m), 3.57 (1H, dd, *J* = 10.24 Hz; *J* = 5.14 Hz), 3.17 (4H, m), 3.04 (2H, q, *J* = 6.71 Hz), 2.80 (2H, t, *J* = 7.10 Hz); ^13^C NMR (DMSO‐*d*_*6*_, 100 MHz): 182.0, 153.0, 146.0, 145.8, 143.9, 143.1, 139.5, 136.1, 135.4, 133.3, 131.6, 131.0, 130.2, 128.5, 128.2, 127.6, 126.6, 124.8, 122.1, 118.5, 116.0, 108.7, 75.5, 68.7, 67.6, 65.9, 51.5, 50.2, 49.1, 44.6, 35.9; ESI‐HRMS (*m*/*z*) calculated for [M+H]^+^ ion species C_39_H_41_Cl_2_N_7_O_7_S_3_ 886.8790, found 886.9796; Elemental analysis, calculated: C, 55.82; H, 4.66; N, 11.06; found: C, 55.85; H, 4.64; N, 11.09.

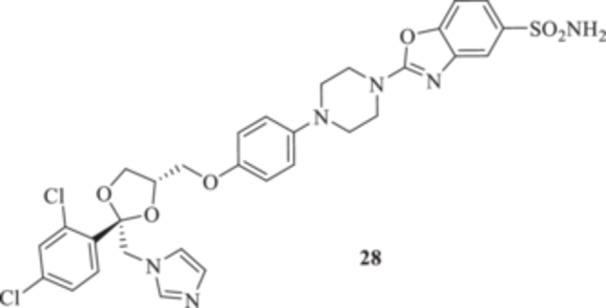

Synthesis of 2‐[4‐(4‐(((2 *R*,4*S*)‐2‐[(1*H*‐Imidazol‐1‐yl)methyl]‐2‐(2,4‐dichlorophenyl)‐1,3‐dioxolan‐4‐yl)methoxy)phenyl)piperazin‐1‐yl]benzo[*d*]oxazole‐5‐sulfonamide (**28**): 1‐(4‐{[(2 *R*,4*S*)‐2‐[(1*H*‐Imidazol‐1‐yl)methyl]‐2‐(2,4‐dichlorophenyl)‐1,3‐dioxolan‐4‐yl]methoxy}phenyl)piperazine (**2**) (94 mg; 0.19 mmol; 1.0 eq.)k and 2‐thioxo‐2,3‐dihydrobenzo[*d*]oxazole‐5‐sulfonamide **27** (44.3 mg; 0.19 mmol; 1.0 eq.) were dissolved in DMF (4 mL). Then, triethylamine (33 µL; 0.23 mmol; 1.2 eq.) was added and the reaction mixture was stirred at reflux temperature for 12 h. A control via TLC was performed to ensure complete consumption of the starting materials. The reaction was quenched with H_2_O, and the product was extracted with EtOAc. The combined organic layers were dried over Na_2_SO_4_ and evaporated under reduced pressure. Purification via silica gel flash chromatography was performed, using 3/97 MeOH/DCM. Red‐brown solid 26% yield; ^1^H NMR (DMSO‐*d*_*6*_, 400 MHz): 7.74 (1H, d, *J* = 1.60 Hz), 7.72 (1H, d, *J* = 2.12 Hz), 7.62 (2H, t, *J* = 8.02 Hz), 7.57 (1H, dd, *J* = 8.36 Hz; *J* = 1.80 Hz), 7.52 (1H, s), 7.49 (1H, dd, *J* = 8.31 Hz; *J* = 1.77 Hz), 7.35 (2H, bs, SO_2_N*H*_2_, exchange with D_2_O), 7.05 (1H, s), 7.01 (2H, d, *J* = 9.08 Hz), 6.87 (1H, s), 6.85 (2H, d, *J* = 3.20 Hz), 4.57 (2H, q, *J* = 11.03 Hz), 4.38 (1H, m), 3.91 (1H, t, *J* = 5.00 Hz), 3.82 (4H, m), 3.69 (2H, m), 3.58 (1H, dd, *J* = 10.14 Hz; *J* = 5.16 Hz), 3.21 (4H, m). ^13^C NMR (DMSO‐*d*_*6*_, 100 MHz): 161.7, 153.1, 151.6, 146.4, 139.5, 136.1, 135.4, 133.3, 132.8, 131.5, 131.0, 128.5, 128.2, 122.0, 121.3, 119.1, 117.5, 116.0, 110.9, 108.6, 75.5, 68.6, 67.6, 51.6, 51.5, 50.5, 45.7; ESI‐HRMS (*m*/*z*) calculated for [M+H]^+^ ion species C_31_H_30_Cl_2_N_6_O_6_S 685.5770, found 685.5776; Elemental analysis, calculated: C, 54.31; H, 4.41; N, 12.26; found: C, 54.28; H, 4.37; N, 12.27.

#### 4.1.5 General Procedure for the Synthesis of Compounds **35a–c**, **36a–c**, **37a–c**, **38**, and **39**

1‐(4‐{[(2 *R*,4*S*)‐2‐[(1*H*‐Imidazol‐1‐yl)methyl]‐2‐(2,4‐dichlorophenyl)‐1,3‐dioxolan‐4‐yl]methoxy}phenyl)piperazine (**2**) (1.0 eq.), K_2_CO_3_ (1.2 eq.), and the appropriate sulfonamide (**30–34)** (1.0 eq.) were dissolved in acetonitrile (4 mL). The reaction mixture was stirred at reflux temperature for 12 h. A control via TLC was performed to ensure complete consumption of the starting materials. The reaction was quenched with H_2_O, and the product was extracted with EtOAc. The combined organic layers were dried over Na_2_SO_4_ and evaporated under reduced pressure. Purification via silica gel flash chromatography was performed to afford the pure products.

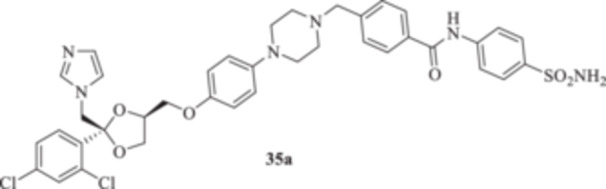

*N*‐(4‐Sulfamoylphenyl)‐4‐[(4‐{4‐[(2 *R*,4*S*)‐2‐[(1*H*‐imidazol‐1‐yl)methyl]‐2‐(2,4‐dichlorophenyl)‐1,3‐dioxolan‐4‐yl]methoxy}phenyl)piperazin‐1‐yl]methyl]benzamide (**35a**): White solid 67% yield; ^1^H NMR (DMSO‐*d*_*6*_, 400 MHz): 10.53 (1H, s, N*H*, exchange with D_2_O), 7.96 (4H, m), 7.82 (2H, d, *J* = 8.64 Hz), 7.68 (1H, d, *J* = 1.68 Hz), 7.57 (1H, d, *J* = 8.48 Hz), 7.50 (3H, m), 7.45 (1H, dd, *J* = 8.46 Hz; *J* = 1.68 Hz), 7.28 (2H, bs, SO_2_N*H*_2_, exchange with D_2_O), 7.01 (1H, s), 6.88 (2H, d, *J* = 8.92 Hz), 6.82 (1H, s), 6.78 (2H, d, *J* = 8.84 Hz), 4.53 (2H, q, *J* = 11.15 Hz), 4.33 (1H, m), 3.86 (1H, t, *J* = 7.44 Hz), 3.64 (4H, m), 3.52 (1H, dd, *J* = 10.03 Hz; *J* = 5.10 Hz), 3.04 (4H, m), 2.57 (4H, m); ^13^C NMR (DMSO‐*d*_*6*_, 100 MHz): 166.9, 152.7, 146.8, 143.5, 143.3, 139.8, 136.3, 135.5, 134.3, 133.4, 131.7, 131.1, 129.9, 128.9, 128.7, 128.3, 127.6, 122.2, 120.8, 118.3, 116.2, 108.8, 75.6, 68.9, 67.8, 62.6, 53.8, 51.6, 50.5, 44.7; ESI‐HRMS (*m*/*z*) calculated for [M+H]^+^ ion species C_38_H_38_Cl_2_N_6_O_6_S 777.7180, found 777.7184; Elemental analysis, calculated: C, 58.69; H, 4.93; N, 10.81; found: C, 58.71; H, 4.97; N, 10.78.

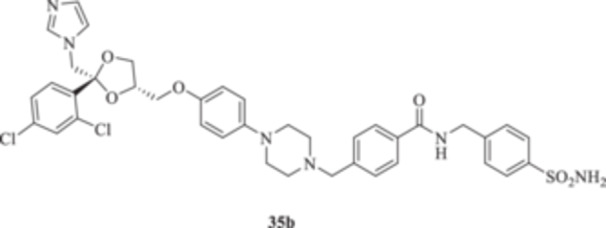

4‐[(4‐{4‐[[(2 *R*,4*S*)‐2‐[(1*H*‐Imidazol‐1‐yl)methyl]‐2‐(2,4‐dichlorophenyl)‐1,3‐dioxolan‐4‐yl]methoxy]phenyl}piperazin‐1‐yl)methyl]‐*N*‐[(4‐sulfamoylphenyl)methyl]benzamide (**35b**): White solid 42% yield; ^1^H NMR (DMSO‐*d*_*6*_, 400 MHz): 9.11 (t, 1H, *J* = 5.78 Hz, N*H*, exchange with D_2_O), 7.90 (2H, d, *J* = 8.04 Hz), 7.81 (2H, d, *J* = 8.20 Hz), 7.69 (1H, d, *J* = 1.88 Hz), 7.58 (1H, d, *J* = 8.44 Hz), 7.52 (1H, s), 7.50 (2H, d, *J* = 2.84 Hz), 7.46 (3H, ap d, *J* = 8.20 Hz), 7.32 (2H, bs, SO_2_N*H*_2_, exchange with D_2_O), 7.03 (1H, s), 6.89 (2H, d, *J* = 9.00 Hz), 6.84 (1H, s), 6.80 (2H, d, *J* = 8.96 Hz), 4.56 (4H, m), 4.35 (1H, m), 3.88 (1H, t, *J* = 7.46 Hz), 3.67 (2H, m), 3.60 (2H, s), 3.55 (1H, dd, *J* = 10.13 Hz; *J* = 5.14 Hz), 3.37 (2H, s), 3.05 (4H, m), 2.11 (2H, ap s); ^13^C NMR (DMSO‐*d*_*6*_, 100 MHz): 167.1, 152.6, 146.7, 144.7, 143.5, 142.6, 139.4, 136.1, 135.4, 133.8, 133.3, 131.5, 131.0, 129.6, 128.5, 128.4, 128.1, 126.6, 122.0, 118.0, 116.0, 108.6, 75.5, 68.7, 67.6, 62.5, 53.6, 51.5, 50.4, 43.2, 31.5; ESI‐HRMS (*m*/*z*) calculated for [M+H]^+^ ion species C_39_H_40_Cl_2_N_6_O_6_S 791.7450, found 791.7458; Elemental analysis, calculated: C, 59.16; H, 5.09; N, 10.61; found: C, 59.12; H, 5.13; N, 10.57.

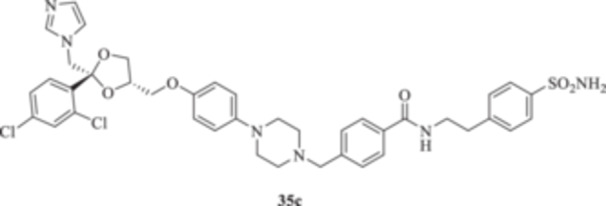

4‐[(4‐{4‐[[(2 *R*,4*S*)‐2‐[(1*H*‐Imidazol‐1‐yl)methyl]‐2‐(2,4‐dichlorophenyl)‐1,3‐dioxolan‐4‐yl]methoxy]phenyl}piperazin‐1‐yl)methyl]‐*N*‐(4‐sulfamoylphenethyl)benzamide (**35c**): White solid 31% yield; ^1^H NMR (DMSO‐*d*_*6*_, 400 MHz): 8.58 (t, 1H, *J* = 5.44 Hz, N*H*, exchange with D_2_O), 7.82 (2H, d, *J* = 8.12 Hz), 7.79 (2H, d, *J* = 8.20 Hz), 7.71 (1H, d, *J* = 1.92 Hz), 7.60 (1H, d, *J* = 8.48 Hz), 7.51 (1H, s), 7.48 (2H, m), 7.44 (3H, m), 7.33 (2H, bs, SO_2_N*H*_2_, exchange with D_2_O), 7.04 (1H, s), 6.90 (2H, d, *J* = 9.04 Hz), 6.85 (1H, s), 6.81 (2H, d, *J* = 8.96 Hz), 4.56 (2H, q, *J* = 11.21 Hz), 4.36 (1H, m), 3.89 (1H, t, *J* = 7.48 Hz), 3.67 (3H, m), 3.56 (6H, m), 3.21 (1H, d, *J* = 5.12 Hz), 3.06 (4H, m), 2.96 (2H, t, *J* = 7.06 Hz), 2.74 (1H, m); ^13^C NMR (DMSO‐*d*_*6*_, 100 MHz): 167.0, 152.6, 146.7, 144.7, 143.0, 142.4, 139.5, 136.1, 135.4, 134.2, 133.3, 131.5, 131.0, 130.0, 129.6, 128.5, 128.2, 128.0, 126.6, 122.0, 118.1, 108.6, 75.5, 68.7, 67.6, 62.5, 54.8, 53.6, 51.5, 50.4, 49.5, 35.7; ESI‐HRMS (*m*/*z*) calculated for [M+H]^+^ ion species C_40_H_42_Cl_2_N_6_O_6_S 805.7720, found 805.7728; Elemental analysis, calculated: C, 59.62; H, 5.25; N, 10.43; found: C, 59.65; H, 5.28; N, 10.41.

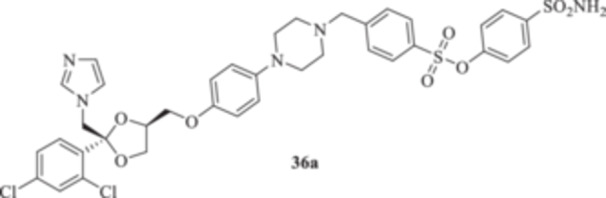

4‐(Sulfamoyl)phenyl 4‐{[4‐(4‐{[(2 R,4S)‐2‐[(1*H*‐imidazol‐1‐yl)methyl]‐2‐(2,4‐dichlorophenyl)‐1,3‐dioxolan‐4‐yl]methoxy}phenyl)piperazin‐1‐yl]methyl}benzenesulfonate (**36a**): White solid 41% yield; ^1^H NMR (DMSO‐*d*_*6*_, 400 MHz): 7.89 (2H, d, *J* = 8.24 Hz), 7.85 (2H, d, *J* = 8.68 Hz), 7.68 (2H, bs, SO_2_N*H*_2_, exchange with D_2_O), 7.65 (1H, s), 7.58 (1H, d, *J* = 8.48 Hz), 7.49 (1H, s), 7.46 (3H, m), 7.28 (2H, d, *J* = 8.68 Hz), 7.02 (1H, s), 6.89 (2H, d, *J* = 9.00 Hz), 6.83 (1H, s), 6.79 (2H, d, *J* = 9.00 Hz), 4.54 (2H, q, *J* = 11.14 Hz), 4.34 (1H, m), 3.87 (1H, t, *J* = 7.48 Hz), 3.66 (4H, m), 3.53 (1H, dd, *J* = 10.18 Hz; *J* = 5.18 Hz), 3.40 (1H, ap t, *J* = 7.02 Hz), 3.05 (4H, m), 2.69 (1H, m), 2.40 (2H, m); ^13^C NMR (DMSO‐*d*_*6*_, 100 MHz): 152.6, 151.8, 147.5, 146.6, 144.0, 139.4, 136.1, 135.4, 133.4, 133.3, 131.5, 131.0, 130.8, 129.2, 128.8, 128.5, 128.2, 126.6, 122.0, 118.1, 108.6, 75.5, 68.7, 67.6, 62.0, 53.6, 51.5, 50.4, 45.4; ESI‐HRMS (*m*/*z*) calculated for [M+H]^+^ ion species C_37_H_37_Cl_2_N_5_O_8_S_2_ 814.7500, found 814.7522; Elemental analysis, calculated: C, 54.55; H, 4.58; N, 8.60; found: C, 54.59; H, 4.63; N, 8.59.

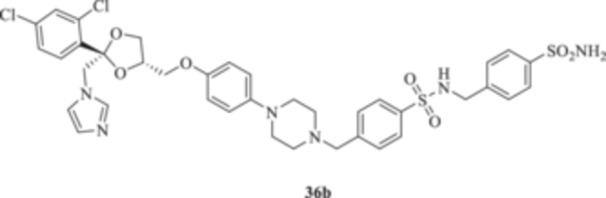

*N*‐[(4‐Sulfamoylphenyl)methyl]‐4‐{[4‐(4‐{[(2 *R*,4*S*)‐2‐[(1*H*‐imidazol‐1‐yl)methyl]‐2‐(2,4‐dichlorophenyl)‐1,3‐dioxolan‐4‐yl]methoxy}phenyl)piperazin‐1‐yl]methyl}benzenesulfonamide (**36b**): White solid 50% yield; ^1^H NMR (DMSO‐*d*_*6*_, 400 MHz): 8.26 (1H, s, N*H*, exchange with D_2_O), 7.78 (2H, d, *J* = 8.08 Hz), 7.73 (2H, d, *J* = 8.16 Hz), 7.69 (1H, d, *J* = 1.68 Hz), 7.58 (1H, d, *J* = 8.48 Hz), 7.54 (2H, d, *J* = 8.12 Hz), 7.49 (1H, s), 7.46 (1H, dd, *J* = 8.52 Hz; *J* = 1.67 Hz), 7.42 (2H, d, *J* = 8.12 Hz), 7.34 (2H, bs, SO_2_N*H*_2_, exchange with D_2_O), 7.02 (1H, s), 6.89 (2H, d, *J* = 8.96 Hz), 6.83 (1H, s), 6.79 (2H, d, *J* = 8.92 Hz), 4.54 (2H, q, *J* = 11.16 Hz), 4.34 (1H, m), 4.09 (2H, s), 3.87 (1H, t, *J* = 7.46 Hz), 3.66 (2H, m), 3.62 (2H, ap s), 3.53 (1H, dd, *J* = 10.12 Hz; *J* = 5.12 Hz), 3.39 (1H, ap t, *J* = 7.10 Hz), 3.06 (4H, m), 2.50 (2H, s), 2.10 (1H, s); ^13^C NMR (DMSO‐*d*_*6*_, 100 MHz): 152.6, 146.6, 144.1, 143.8, 142.8, 140.1, 139.4, 136.1, 135.4, 133.3, 131.5, 131.0, 130.3, 128.8, 128.5, 128.2, 127.4, 126.5, 118.1, 116.0, 108.6, 75.5, 68.7, 67.6, 65.8, 62.2, 53.6, 51.5, 46.5, 16.1; ESI‐HRMS (*m*/*z*) calculated for [M+H]^+^ ion species C_38_H_40_Cl_2_N_6_O_7_S_2_ 827.7930, found 827.7936; Elemental analysis, calculated: C, 55.14; H, 4.87; N, 10.15; found: C, 55.12; H, 4.90; N, 10.17.

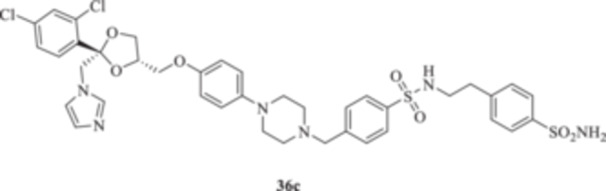

4‐[(4‐{4‐[[(2 *R*,4*S*)‐2‐[(1*H*‐Imidazol‐1‐yl)methyl]‐2‐(2,4‐dichlorophenyl)‐1,3‐dioxolan‐4‐yl]methoxy]phenyl}piperazin‐1‐yl)methyl]‐*N*‐(4‐sulfamoylphenethyl)benzenesulfonamide (**36c**): White solid 28% yield; ^1^H NMR (DMSO‐*d*_*6*_, 400 MHz): 7.76 (4H, t, *J* = 8.44 Hz), 7.72 (2H, m), 7.59 (2H, m), 7.56 (1H, s), 7.51 (1H, bs), 7.48 (1H, dd, *J* = 8.48 Hz; *J* = 2.00 Hz), 7.37 (2H, d, *J* = 8.20 Hz), 7.33 (2H, bs, SO_2_N*H*_2_, exchange with D_2_O), 7.04 (1H, s, N*H*, exchange with D_2_O), 6.90 (2H, d, *J* = 9.04 Hz), 6.85 (1H, s), 6.81 (2H, s, *J* = 9.00 Hz), 4.56 (2H, q, *J* = 11.16 Hz), 4.36 (1H, m), 3.89 (1H, t, *J* = 7.48 Hz), 3.67 (2H, m), 3.64 (2H, ap s), 3.55 (1H, dd, *J* = 10.19 Hz; *J* = 5.18 Hz), 3.41 (1H, ap q, *J* = 7.04 Hz), 3.04 (6H, m), 2.79 (2H, t, *J* = 7.06 Hz), 2.51 (1H, s), 2.12 (1H, s), 1.18 (1H, s); ^13^C NMR (DMSO‐*d*_*6*_, 100 MHz): 152.6, 146.6, 144.0, 143.8, 143.1, 139.8, 136.1, 135.4, 133.3, 131.5, 131.0, 131.3, 130.1, 128.5, 128.2, 127.4, 126.6, 122.0, 118.1, 116.0, 108.6, 75.5, 68.7, 67.6, 65.8, 53.6, 51.5, 50.4, 44.5, 30.5, 16.1; ESI‐HRMS (*m*/*z*) calculated for [M+H]^+^ ion species C_39_H_42_Cl_2_N_6_O_7_S_2_ 841.8200, found 841.8212; Elemental analysis, calculated: C, 55.64; H, 5.03; N, 9.98; found: C, 55.65; H, 5.01; N, 9.97.

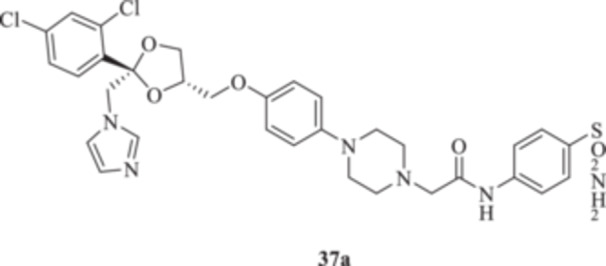

*N*‐(4‐Sulfamoylphenyl)‐2‐[4‐(4‐{[(2 *R*,4*S*)‐2‐[(1*H*‐imidazol‐1‐yl)methyl]‐2‐(2,4‐dichlorophenyl)‐1,3‐dioxolan‐4‐yl]methoxy}phenyl)piperazin‐1‐yl]acetamide (**37a**): White solid 27% yield; ^1^H NMR (DMSO‐*d*_*6*_, 400 MHz): 10.15 (1H, s, N*H*, exchange with D_2_O), 7.86 (2H, d, *J* = 8.44 Hz), 7.80 (2H, d, *J* = 8.64 Hz), 7.73 (1H, d, *J* = 1.84 Hz), 7.61 (1H, d, *J* = 8.36 Hz), 7.52 (1H, s), 7.49 (1H, m), 7.30 (2H, bs, SO_2_N*H*_2_, exchange with D_2_O), 7.05 (1H, s), 6.93 (2H, d, *J* = 8.40 Hz), 6.84 (2H, d, *J* = 9.40 Hz), 6.81 (1H, s), 4.56 (2H, q, *J* = 10.71 Hz), 4.37 (1H, m), 3.90 (1H, t, *J* = 7.11 Hz), 3.68 (2H, m), 3.55 (1H, m), 3.27 (2H, s), 3.13 (4H, m), 2.71 (4H, m); ^13^C NMR (DMSO‐*d*_*6*_, 100 MHz): 169.0, 152.7, 146.5, 136.2, 135.4, 133.3, 131.6, 131.0, 128.5, 128.2, 127.0, 122.8, 122.1, 118.2, 117.6, 116.0, 114.9, 108.7, 75.5, 68.7, 67.6, 62.1, 53.7, 51.5, 50.8, 48.8; ESI‐HRMS (*m*/*z*) calculated for [M+H]^+^ ion species C_32_H_34_Cl_2_N_6_O_6_S 701.6200, found 701.6190; Elemental analysis, calculated: C, 54.78; H, 4.88; N, 11.98; found: C, 54.80; H, 5.01; N, 12.03.

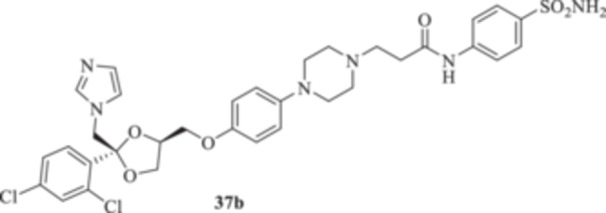

*N*‐(4‐Sulfamoylphenyl)‐3‐[4‐(4‐{[(2 *R*,4*S*)‐2‐[(1*H*‐imidazol‐1‐yl)methyl]‐2‐(2,4‐dichlorophenyl)‐1,3‐dioxolan‐4‐yl]methoxy}phenyl)piperazin‐1‐yl]propanamide (**37b**): White solid 44% yield; ^1^H NMR (DMSO‐*d*_*6*_, 400 MHz): 10.44 (1H, s, N*H*, exchange with D_2_O), 7.78 (4H, ap s), 7.73 (1H, d, *J* = 2.00 Hz), 7.60 (1H, d, *J* = 8.44 Hz), 7.51 (1H, s), 7.49 (1H, dd, *J* = 8.65 Hz; *J* = 2.14 Hz), 7.28 (2H, bs, SO_2_N*H*_2_, exchange with D_2_O), 7.05 (1H, s), 6.92 (2H, d, *J* = 9.04 Hz), 6.85 (1H, s), 6.81 (2H, d, *J* = 9.04 Hz), 4.56 (2H, q, *J* = 11.19 Hz), 4.37 (1H, m), 3.90 (1H, t, *J* = 7.50 Hz), 3.67 (2H, m), 3.54 (1H, dd, *J* = 10.20 Hz; *J* = 5.27 Hz), 3.06 (4H, m), 2.73 (2H, t, *J* = 6.86 Hz), 2.60 (6H, m); ^13^C NMR (DMSO‐*d*_*6*_, 100 MHz): 171.7, 152.6, 146.6, 143.0, 139.4, 139.1, 136.9, 136.1, 135.4, 133.3, 131.5, 128.5, 128.2, 127.6, 122.0, 118.0, 116.0, 108.6, 75.5, 68.7, 67.6, 54.5, 53.4, 51.5, 50.4, 35.1, 30.5; ESI‐HRMS (*m*/*z*) calculated for [M+H]^+^ ion species C_33_H_36_Cl_2_N_6_O_6_S 715.6470, found 715.6478; Elemental analysis, calculated: C, 55.39; H, 5.07; N, 11.74; found: C, 55.42; H, 5.03; N, 11.71.

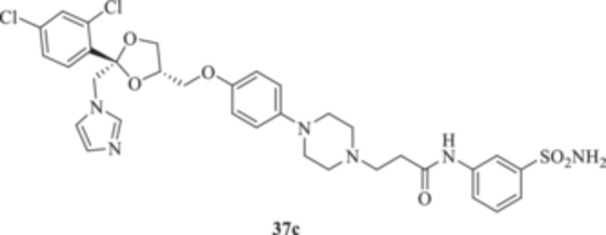

*N*‐(3‐Sulfamoylphenyl)‐3‐[4‐(4‐{[(2 *R*,4*S*)‐2‐[(1*H*‐imidazol‐1‐yl)methyl]‐2‐(2,4‐dichlorophenyl)‐1,3‐dioxolan‐4‐yl]methoxy}phenyl)piperazin‐1‐yl]propanamide (**37c**): White solid 59% yield; ^1^H NMR (DMSO‐*d*_*6*_, 400 MHz): 10.39 (1H, s, N*H*, exchange with D_2_O), 8.21 (1H, s), 7.76 (1H, m), 7.72 (1H, d, *J* = 2.04 Hz), 7.61 (1H, d, *J* = 8.48 Hz), 7.53 (1H, s), 7.52 (2H, ap d, *J* = 1.96 Hz), 7.49 (1H, dd, *J* = 8.51 Hz; *J* = 2.04 Hz), 7.40 (2H, bs, SO_2_N*H*_2_, exchange with D_2_O), 7.05 (1H, s), 6.92 (2H, d, *J* = 9.04 Hz), 6.85 (1H, s), 6.81 (2H, d, *J* = 9.00 Hz), 4.56 (2H, q, *J* = 11.25 Hz), 4.37 (1H, m), 3.90 (1H, t, *J* = 7.52 Hz), 3.67 (2H, m), 3.54 (1H, dd, *J* = 10.11 Hz; *J* = 5.19 Hz), 3.06 (4H, m), 2.74 (2H, t, *J* = 6.86 Hz), 2.59 (6H, m, *J* = 5.54 Hz); ^13^C NMR (DMSO‐*d*_*6*_, 100 MHz): 171.5, 152.6, 146.7, 140.5, 139.5, 136.1, 135.4, 133.3, 131.5, 131.0, 130.4, 128.5, 128.2, 122.7, 122.0, 121.0, 118.0, 117.0, 116.0, 108.6, 75.5, 68.7, 67.6, 54.6, 53.5, 51.5, 50.4, 35.1, 31.6; ESI‐HRMS (*m*/*z*) calculated for [M+H]^+^ ion species C_33_H_36_Cl_2_N_6_O_6_S 715.6470, found 715.6468; Elemental analysis, calculated: C, 55.39; H, 5.07; N, 11.74; found: C, 55.41; H, 5.05; N, 11.77.

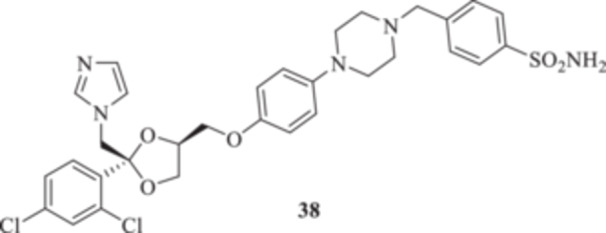

4‐[(4‐(4‐{[(2 *R*,4*S*)‐2‐[(1*H*‐Imidazol‐1‐yl)methyl]‐2‐(2,4‐dichlorophenyl)‐1,3‐dioxolan‐4‐yl]methoxy}phenyl)piperazin‐1‐yl)methyl]benzenesulfonamide (**38**): White solid 42% yield; ^1^H NMR (DMSO‐*d*_*6*_, 400 MHz): 7.83 (2H, d, *J* = 8.12 Hz), 7.70 (1H, d, *J* = 1.88 Hz), 7.59 (1H, d, *J* = 8.48 Hz), 7.55 (2H, d, *J* = 8.16 Hz), 7.50 (1H, s), 7.47 (1H, dd, *J* = 8.55 Hz; *J* = 2.09 Hz), 7.35 (2H, bs, SO_2_N*H*_2_, exchange with D_2_O), 7.04 (1H, s), 6.89 (2H, d, *J* = 9.00 Hz), 6.84 (1H, s), 6.80 (2H, d, *J* = 9.00 Hz), 4.55 (2H, q, *J* = 11.20 Hz), 4.36 (1H, m), 3.89 (1H, t, *J* = 7.49 Hz), 3.66 (4H, m), 3.53 (1H, dd, *J* = 10.23 Hz; *J* = 5.11 Hz), 3.41 (1H, q, *J* = 7.00 Hz), 3.06 (4H, m), 2.98 (1H, ap t, *J* = 4.78 Hz), 2.89 (1H, ap t, *J* = 4.68 Hz), 2.51 (1H, m); ^13^C NMR (DMSO‐*d*_*6*_, 100 MHz): 152.6, 146.6, 143.3, 139.4, 136.1, 135.4, 133.3, 131.5, 131.0, 130.0, 128.5, 128.2, 126.6, 122.0, 118.1, 116.0, 108.6, 75.5, 68.7, 67.6, 65.8, 62.2, 53.6, 51.5, 50.4; ESI‐HRMS (*m*/*z*) calculated for [M+H]^+^ ion species C_31_H_33_Cl_2_N_5_O_5_S 658.5950, found 658.5948; Elemental analysis, calculated: C, 56.54; H, 5.05; N, 10.63; found: C, 56.57; H, 5.02; N, 10.60.

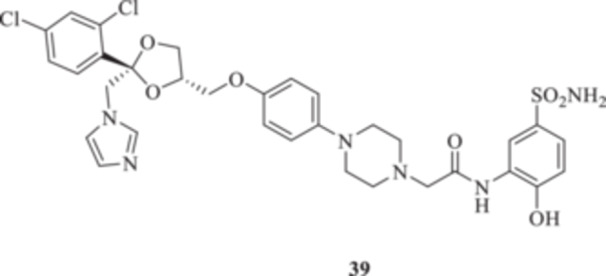

*N*‐[2‐Hydroxy‐5‐(sulfamoyl)phenyl]‐2‐[4‐(4‐{[(2 *R*,4*S*)‐2‐[(1*H*‐imidazol‐1‐yl)methyl]‐2‐(2,4‐dichlorophenyl)‐1,3‐dioxolan‐4‐yl]methoxy}phenyl)piperazin‐1‐yl]acetamide (**39**): White solid 40% yield; ^1^H NMR (DMSO‐*d*_*6*_, 400 MHz): 11.04 (1H, bs, O*H*, exchange with D_2_O), 9.84 (1H, s, N*H*, exchange with D_2_O), 8.73 (1H, s), 7.72 (1H, s), 7.61 (1H, d, *J* = 8.44 Hz), 7.52 (1H, s), 7.49 (1H, m), 7.43 (1H, d, *J* = 7.60 Hz), 7.17 (2H, bs, SO_2_N*H*_2_, exchange with D_2_O), 7.05 (s, 1H), 6.98 (2H, ap t, *J* = 8.28 Hz), 6.94 (1H, s), 6.86 (1H, s), 6.84 (2H, d, *J* = 8.88 Hz), 4.57 (2H, q, *J* = 10.98 Hz), 4.38 (1H, m), 3.91 (1H, t, *J* = 7.46 Hz), 3.69 (2H, m), 3.57 (1H, dd, *J* = 10.04 Hz; *J* = 5.06 Hz), 3.27 (2H, s), 3.15 (4H, m), 2.75 (4H, m); ^13^C NMR (DMSO‐*d*_*6*_, 100 MHz): 169.0, 152.7, 146.5, 139.5, 136.2, 135.6, 135.4, 133.3, 131.6, 131.0, 128.5, 128.2, 127.0, 122.8, 122.1, 118.2, 116.0, 114.9, 114.2, 108.6, 82.1, 75.5, 68.7, 67.6, 62.1, 55.2, 53.7, 51.5, 50.8; ESI‐HRMS (*m*/*z*) calculated for [M+H]^+^ ion species C_32_H_34_Cl_2_N_6_O_7_S 717.6190, found 717.6188; Elemental analysis, calculated: C, 53.56; H, 4.78; N, 11.71; found: C, 53.58; H, 4.76; N, 11.67.

#### 4.1.6 General Procedure for the Synthesis of Compounds **37b,c**, **48a–d**, and **49a–d**

1‐(4‐(((2 *R*,4*S*)‐2‐((1*H*‐Imidazol‐1‐yl)methyl)‐2‐(2,4‐dichlorophenyl)‐1,3‐dioxolan‐4‐yl)methoxy)phenyl)piperazine (**2**) (1.0 eq.) and the appropriate acryloyl derivative (**43–45**) (1.0 eq.) were dissolved in methanol (4–5 mL). The reaction mixture was stirred at reflux temperature for 12 h. A control via TLC was performed to ensure complete consumption of the starting materials. The reaction was quenched with H_2_O, and the precipitate was filtered off under vacuum, washed with Et_2_O and H_2_O, and then dried. Purification via silica gel flash chromatography was performed to afford the pure products.

*N*‐(4‐Sulfamoylphenyl)‐3‐[4‐(4‐{[(2 *R*,4*S*)‐2‐[(1*H*‐imidazol‐1‐yl)methyl]‐2‐(2,4‐dichlorophenyl)‐1,3‐dioxolan‐4‐yl]methoxy}phenyl)piperazin‐1‐yl]propanamide (**37b**): Yield 42%. Experimental as reported above.

*N*‐(3‐Sulfamoylphenyl)‐3‐[4‐(4‐{[(2 *R*,4*S*)‐2‐[(1*H*‐imidazol‐1‐yl)methyl]‐2‐(2,4‐dichlorophenyl)‐1,3‐dioxolan‐4‐yl]methoxy}phenyl)piperazin‐1‐yl]propanamide (**37c**): Yield 48%. Experimental as reported above.

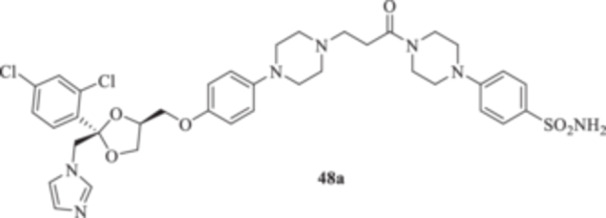

4‐[4‐(3‐[4‐(4‐{[(2 *R*,4*S*)‐2‐[(1*H*‐Imidazol‐1‐yl)methyl]‐2‐(2,4‐dichlorophenyl)‐1,3‐dioxolan‐4‐yl]methoxy}phenyl)piperazin‐1‐yl]propanoyl)piperazin‐1‐yl]benzenesulfonamide (**48a**): White solid 61% yield; ^1^H NMR (DMSO‐*d*_*6*_, 400 MHz): 7.73 (1H, s), 7.67 (2H, d, *J* = 8.64 Hz), 7.61 (1H, d, *J* = 8.48 Hz), 7.52 (1H, s), 7.50 (1H, d, *J* = 8.68 Hz), 7.12 (2H, bs, SO_2_N*H*_2_, exchange with D_2_O), 7.09 (1H, s), 7.06 (2H, d, *J* = 6.28 Hz), 6.91 (2H, d, *J* = 8.88 Hz), 6.86 (1H, s), 6.81 (2H, d, *J* = 8.76 Hz), 4.56 (2H, q, *J* = 11.39 Hz), 4.37 (1H, m), 3.90 (1H, t, *J* = 7.56 Hz), 3.67 (6H, m), 3.53 (1H, dd, *J* = 10.00 Hz; *J* = 4.90 Hz), 3.46 (1H, m), 3.30 (2H, m), 3.04 (4H, m), 2.63 (4H, m), 2.58 (4H, m), 1.34 (1H, m); ^13^C NMR (DMSO‐*d*_*6*_, 100 MHz): 170.8, 153.4, 152.5, 146.7, 136.1, 135.4, 134.0, 133.3, 131.5, 131.0, 128.5, 128.2, 128.0, 122.0, 118.0, 116.0, 114.8, 108.6, 75.5, 68.7, 67.6, 54.7, 53.7 51.5, 50.3, 48.2, 47.8, 45.3, 41.4; ESI‐HRMS (*m*/*z*) calculated for [M+H]^+^ ion species C_37_H_43_Cl_2_N_7_O_6_S 784.7540, found 784.7546; Elemental analysis, calculated: C, 56.63; H, 5.52; N, 12.49; found: C, 56.67; H, 5.49; N, 12.47.

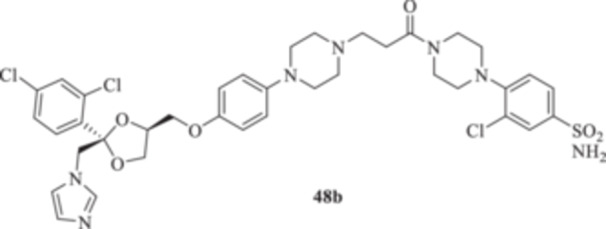

4‐[4‐(3‐[4‐(4‐{[(2 *R*,4*S*)‐2‐[(1*H*‐Imidazol‐1‐yl)methyl]‐2‐(2,4‐dichlorophenyl)‐1,3‐dioxolan‐4‐yl]methoxy}phenyl)piperazin‐1‐yl]propanoyl)piperazin‐1‐yl]‐3‐chlorobenzenesulfonamide (**48b**): White solid 49% yield; ^1^H NMR (DMSO‐*d*_*6*_, 400 MHz): 7.86 (1H, d, *J* = 2.12 Hz), 7.75 (1H, dd, *J* = 8.53 Hz; *J* = 2.08 Hz), 7.73 (1H, d, *J* = 2.08 Hz), 7.61 (1H, d, *J* = 8.48 Hz), 7.52 (1H, s), 7.49 (1H, dd, *J* = 8.48 Hz; *J* = 2.11 Hz), 7.42 (2H, bs, SO_2_N*H*_2_, exchange with D_2_O), 7.33 (1H, d, *J* = 8.56 Hz), 7.05 (1H, s), 6.92 (2H, d, *J* = 9.16 Hz), 6.86 (1H, s), 6.81 (2H, d, *J* = 9.08 Hz), 4.56 (2H, q, *J* = 11.28 Hz), 4.37 (1H, m), 3.90 (1H, t, *J* = 5.01 Hz), 3.68 (4H, m), 3.65 (2H, m), 3.54 (1H, dd, *J* = 10.22 Hz; *J* = 5.28 Hz), 3.11 (2H, ap s), 3.05 (6H, m), 2.64 (4H, m), 2.59 (4H, m); ^13^C NMR (DMSO‐*d*_*6*_, 100 MHz): 170.8, 152.5, 152.3, 146.7, 139.9, 139.4, 136.1, 135.4, 133.3, 131.5, 131.0, 128.7, 128.5, 128.2, 128.0, 126.6, 122.0, 118.0, 116.0, 108.6, 75.5, 68.7, 67.6, 54.7, 53.7, 51.8, 51.5, 51.3, 50.3, 46.0, 42.0; ESI‐HRMS (*m*/*z*) calculated for [M+H]^+^ ion species C_37_H_42_Cl_3_N_7_O_6_S 818.1916, found 818.2004; Elemental analysis, calculated: C, 54.25; H, 5.17; N, 11.97; found: C, 54.28; H, 5.16; N, 11.95.

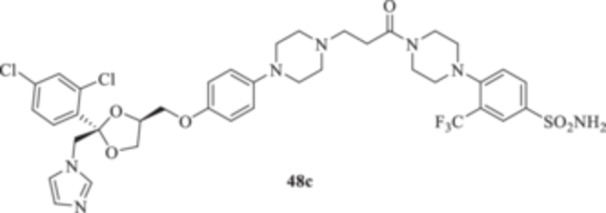

4‐[4‐(3‐[4‐(4‐{[(2 *R*,4*S*)‐2‐[(1*H*‐Imidazol‐1‐yl)methyl]‐2‐(2,4‐dichlorophenyl)‐1,3‐dioxolan‐4‐yl]methoxy}phenyl)piperazin‐1‐yl]propanoyl)piperazin‐1‐yl]‐3‐(trifluoromethyl)benzenesulfonamide (**48c**): White solid 57% yield; ^1^H NMR (400 MHz, DMSO‐*d*_*6*_) *δ*(ppm): 8.12 (1H, s), 8.09 (1H, d, *J* = 8.52 Hz), 7.73 (2H, m), 7.61 (1H, d, *J* = 8.47 Hz), 7.53 (2H, bs, SO_2_N*H*_2_, exchange with D_2_O), 7.51 (1H, s), 7.49 (1H, dd, *J* = 8.54 Hz; *J* = 2.06 Hz), 7.05 (1H, s), 6.92 (2H, d, *J* = 9.08 Hz), 6.85 (1H, s), 6.81 (2H, d, *J* = 9.07 Hz), 4.56 (2H, q, *J* = 11.27 Hz), 4.37 (1H, m), 3.90 (1H, t, *J* = 5.03 Hz), 3.67 (6H, m), 3.55 (1H, dd, *J* = 10.08 Hz; *J* = 5.08 Hz), 3.05 (4H, m), 2.99 (2H, m), 2.94 (2H, m), 2.63 (4H, m), 2.59 (4H, m); ^13^C NMR (100 MHz, DMSO‐*d*_*6*_) *δ*(ppm): 170.8, 155.6, 152.6, 146.8, 143.3, 139.6, 138.2, 136.3, 135.5, 133.4, 131.7, 131.1, 128.6, 128.3, 123.7, 122.1, 120.2, 118.1, 116.1, 114.9, 114.7, 108.7, 75.6, 68.8, 67.7, 54.8, 53.8, 51.6, 51.0, 50.4, 45.8, 41.8; ESI‐HRMS (*m*/*z*) calculated for [M+H]^+^ ion species C_38_H_42_Cl_2_F_3_N_7_O_6_S 852.7522, found 852.7526; Elemental analysis, calculated: C, 53.52; H, 4.96; N, 11.50; found: C, 53.49; H, 4.93; N, 11.47.

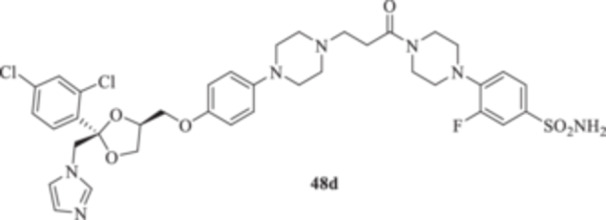

4‐[4‐(3‐[4‐(4‐{[(2 *R*,4*S*)‐2‐[(1*H*‐Imidazol‐1‐yl)methyl]‐2‐(2,4‐dichlorophenyl)‐1,3‐dioxolan‐4‐yl]methoxy}phenyl)piperazin‐1‐yl]propanoyl)piperazin‐1‐yl]‐3‐fluorobenzenesulfonamide (**48d**): White solid 70% yield; ^1^H NMR (DMSO‐*d*_*6*_, 400 MHz): 7.73 (1H, d, *J* = 2.04 Hz), 7.58 (3H, m), 7.52 (1H, s), 7.49 (1H, dd, *J* = 8.50 Hz; *J* = 2.09 Hz), 7.36 (2H, bs, SO_2_N*H*_2_, exchange with D_2_O), 7.21 (1H, t, *J* = 8.70 Hz), 7.05 (1H, s), 6.92 (2H, d, *J* = 9.12 Hz), 6.86 (1H, s), 6.81 (2H, d, *J* = 9.08 Hz), 4.56 (2H, q, *J* = 11.35 Hz), 4.37 (1H, m), 3.90 (1H, t, *J* = 7.52 Hz), 3.67 (6H, m), 3.53 (1H, dd, *J* = 10.11 Hz; *J* = 5.20 Hz), 3.43 (1H, t, *J* = 7.00 Hz), 3.20 (3H, d, *J* = 5.20 Hz), 3.11 (2H, m), 3.05 (4H, m), 2.61 (6H, m); ^13^C NMR (DMSO‐*d*_*6*_, 100 MHz): 170.7, 152.5 (d, *J* ^1^_C‐F_ = 248.2 Hz), 146.7, 143.2 (q, *J* ^3^_C‐F_ = 7.32 Hz), 139.5, 138.1 (q, *J* ^3^_C‐F_ = 5.97 Hz), 136.1, 135.4 (*J* ^1^_C‐F_ = 275.0 Hz), 133.3, 131.5, 131.0, 128.5 (t, *J* ^2^_C‐F_ = 35.45 Hz), 128.2, 123.6, 122.0, 120.0, 118.0, 116.0, 114.5, 108.6, 75.5, 68.7, 67.6, 54.7, 53.7, 51.5 (t, *J* ^2^_C‐F_ = 38.3 Hz), 50.9, 50.3, 45.7 (t, *J* ^2^_C‐F_ = 18.8 Hz), 41.7, 31.6; ESI‐HRMS (*m*/*z*) calculated for [M+H]^+^ ion species C_37_H_42_Cl_2_FN_7_O_6_S 802.7444, found 802.7452; Elemental analysis, calculated: C, 55.36; H, 5.27; N, 12.21; found: C, 55.38; H, 5.29; N, 12.18.

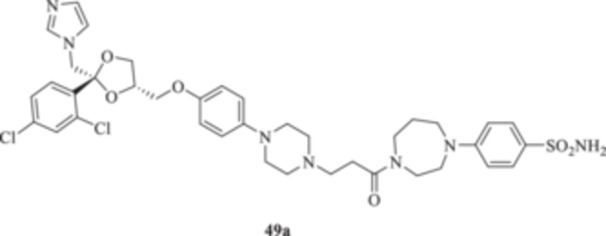

4‐[4‐(3‐[4‐(4‐{[(2 *R*,4*S*)‐2‐[(1*H*‐Imidazol‐1‐yl)methyl]‐2‐(2,4‐dichlorophenyl)‐1,3‐dioxolan‐4‐yl]methoxy}phenyl)piperazin‐1‐yl]propanoyl)‐1,4‐diazepan‐1‐yl]benzenesulfonamide (**49a**): White solid 52% yield; ^1^H NMR (DMSO‐*d*_*6*_, 400 MHz): 7.73 (1H, d, *J* = 1.96 Hz), 7.62 (2H, t, *J* = 4.48 Hz), 7.60 (1H, s), 7.52 (1H, s), 7.50 (1H, dd, *J* = 8.92 Hz; *J* = 2.32 Hz), 7.05 (1H, s), 7.03 (2H, bs, SO_2_N*H*_2_, exchange with D_2_O), 6.88 (5H, m), 6.81 (2H, dd, *J* = 8.93 Hz; *J* = 1.76 Hz), 4.57 (2H, q, *J* = 12.16 Hz), 4.37 (1H, m), 3.89 (1H, t, *J* = 7.46 Hz), 3.77 (1H, m), 3.65 (6H, m), 3.53 (1H, dd, *J* = 9.67 Hz; *J* = 4.73 Hz), 3.43 (1H, s), 3.01 (4H, m), 2.52 (6H, m), 2.12 (6H, s); ^13^C NMR (DMSO‐*d*_*6*_, 100 MHz): 171.3, 152.5, 150.3, 146.7, 139.4, 136.1, 135.4, 133.3, 132.5, 131.5, 131.2, 131.0, 128.5, 128.2, 122.0, 118.0, 116.0, 108.6, 75.5, 68.7, 67.6, 54.8, 53.7, 51.5, 50.6, 50.3, 48.1, 46.9, 45.0, 31.0, 30.7, 30.5; ESI‐HRMS (*m*/*z*) calculated for [M+H]^+^ ion species C_38_H_45_Cl_2_N_7_O_6_S 798.7810, found 798.7828; Elemental analysis, calculated: C, 57.14; H, 5.68; N, 12.27; found: C, 57.18; H, 5.65; N, 12.24.

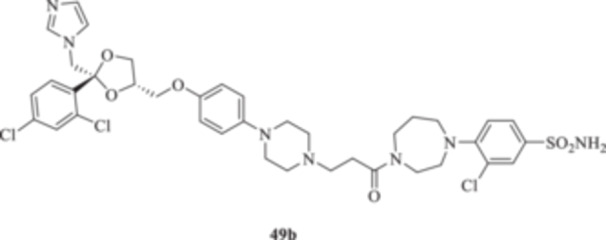

4‐[4‐(3‐[4‐(4‐{[(2 *R*,4*S*)‐2‐[(1*H*‐Imidazol‐1‐yl)methyl]‐2‐(2,4‐dichlorophenyl)‐1,3‐dioxolan‐4‐yl]methoxy}phenyl)piperazin‐1‐yl]propanoyl)‐1,4‐diazepan‐1‐yl]‐3‐chlorobenzenesulfonamide (**49b**): Light yellow solid 86% yield; ^1^H NMR (DMSO‐*d*_*6*_, 400 MHz): 7.81 (1H, m), 7.72 (1H, d, *J* = 2.04 Hz), 7.68 (1H, m), 7.61 (1H, d, *J* = 8.44 Hz), 7.51 (1H, s), 7.49 (1H, dd, *J* = 8.51 Hz; *J* = 2.09 Hz), 7.37 (2H, bs, SO_2_N*H*_2_, exchange with D_2_O), 7.33 (1H, d, *J* = 8.68 Hz), 7.05 (1H, s), 6.90 (2H, m), 6.86 (1H, s), 6.81 (2H, d, *J* = 9.08 Hz), 4.56 (2H, q, *J* = 11.30 Hz), 4.37 (1H, m), 3.90 (1H, t, *J* = 7.48 Hz), 3.67 (6H, m), 3.55 (1H, m), 3.46 (1H, m), 3.35 (6H, m), 2.61 (6H, m), 2.03 (4H, ap s), 1.95 (1H, m); ^13^C NMR (DMSO‐*d*_*6*_, 100 MHz): 153.5, 153.4, 152.5, 146.7, 139.5, 138.5, 136.1, 135.4, 133.3, 131.5, 131.0, 129.0, 128.5, 128.2, 126.5, 126.3, 122.0, 118.0, 116.0, 108.6, 75.5, 68.7, 67.6, 60.7, 54.8, 53.8, 53.6, 51.5, 48.8, 47.1, 46.4, 44.6, 29.7, 28.2; ESI‐HRMS (*m*/*z*) calculated for [M+H]^+^ ion species C_38_H_44_Cl_3_N_7_O_6_S 832.2230, found 832.2224; Elemental analysis, calculated: C, 54.78; H, 5.32; N, 11.77; found: C, 54.81; H, 5.35; N, 11.74.

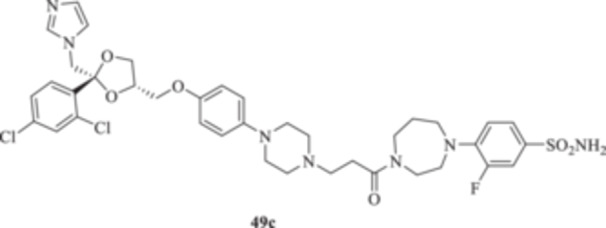

4‐[4‐(3‐[4‐(4‐{[(2 *R*,4*S*)‐2‐[(1*H*‐Imidazol‐1‐yl)methyl]‐2‐(2,4‐dichlorophenyl)‐1,3‐dioxolan‐4‐yl]methoxy}phenyl)piperazin‐1‐yl]propanoyl)‐1,4‐diazepan‐1‐yl]‐3‐fluorobenzenesulfonamide (**49c**): White solid 29% yield; ^1^H NMR (DMSO‐*d*_*6*_, 400 MHz): 7.73 (1H, d, *J* = 2.04 Hz), 7.61 (1H, d, *J* = 8.48 Hz), 7.52 (1H, s), 7.49 (2H, dd, *J* = 8.46 Hz; *J* = 2.05 Hz), 7.47 (1H, d, *J* = 5.27 Hz), 7.23 (2H, bs, SO_2_N*H*_2_, exchange with D_2_O), 7.10 (1H, q, *J* = 8.35 Hz), 7.05 (1H, s), 6.90 (2H, t, *J* = 8.16 Hz), 6.86 (1H, s), 6.81 (2H, m), 4.56 (2H, q, *J* = 11.38 Hz), 4.37 (1H, m), 3.90 (1H, t, *J* = 7.50 Hz), 3.67 (5H, m), 3.53 (5H, m), 3.47 (1H, t, *J* = 5.60 Hz), 3.00 (4H, m), 2.95 (1H, m), 2.52 (4H, m), 2.03 (3H, s), 1.92 (1H, m), 1.84 (1H, m); ^13^C NMR (DMSO‐*d*_*6*_, 100 MHz): 171.6, 152.5, 151.6 (*J* ^1^_C‐F_ = 280.53 Hz), 146.7, 139.4 (d, *J* ^3^_C‐F_ = 6.90 Hz), 136.1, 135.4, 134.2 (*J* ^2^_C‐F_ = 30.5 Hz), 133.3 (d, *J* ^3^_C‐F_ = 6.48 Hz), 131.5, 131.0, 128.5, 128.2, 123.8, 122.0, 118.5, 118.0, 117.5 (d, *J* ^3^_C‐F_ = 4.66 Hz), 113.8 (d, *J* ^2^_C‐F_ = 25.44 Hz), 108.6, 75.5, 68.7, 67.6, 60.6, 54.8 (d, *J* ^3^_C‐F_ = 5.01 Hz), 51.5 (d, *J* ^3^_C‐F_ = 5.34 Hz), 50.3, 49.4, 48.2, 45.0, 28.9, 26.8, 21.6, 15.0; ESI‐HRMS (*m*/*z*) calculated for [M+H]^+^ ion species C_38_H_44_Cl_2_FN_7_O_6_S 816.7714, found 816.7728; Elemental analysis, calculated: C, 55.88; H, 5.43; N, 12.00; found: C, 55.91; H, 5.46; N, 12.03.

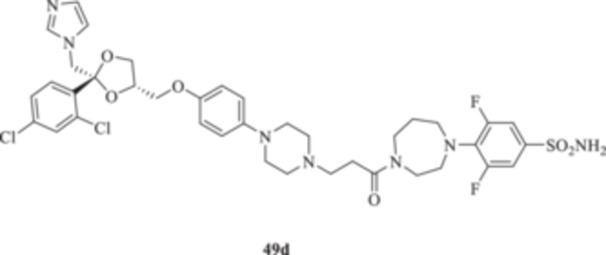

4‐[4‐(3‐[4‐(4‐{[(2 *R*,4*S*)‐2‐[(1*H*‐Imidazol‐1‐yl)methyl]‐2‐(2,4‐dichlorophenyl)‐1,3‐dioxolan‐4‐yl]methoxy}phenyl)piperazin‐1‐yl]propanoyl)‐1,4‐diazepan‐1‐yl]‐3,5‐difluorobenzenesulfonamide (**49d**): White solid 56% yield; ^1^H NMR (DMSO‐*d*_*6*_, 400 MHz): 7.71 (1H, d, *J* = 1.96 Hz), 7.60 (1H, d, *J* = 8.48 Hz), 7.51 (1H, s), 7.46 (5H, m), 7.04 (1H, s), 6.90 (2H, d, *J* = 7.04 Hz), 6.85 (1H, s), 6.80 (2H, d, *J* = 8.88 Hz), 4.56 (2H, q, *J* = 11.23 Hz), 4.36 (1H, m), 3.89 (1H, t, *J* = 7.44 Hz), 3.67 (5H, m), 3.60 (1H, ap t, *J* = 5.96 Hz), 3.54 (1H, dd, *J* = 10.12 Hz; *J* = 5.19 Hz), 3.49 (1H, m), 3.41 (3H, ap q, *J* = 6.98 Hz), 3.03 (4H, m), 2.61 (6H, m), 2.12 (2H, s), 1.92 (1H, s), 1.82 (1H, s); ^13^C NMR (DMSO‐*d*_*6*_, 100 MHz): 171.7, 171.5, 158.0 (dd, *J* ^1^_C‐F_ = 248.15; 7.94 Hz), 155.4, 152.5, 146.7, 139.5 (t, *J* ^2^_C‐F_ = 12.28 Hz), 138.0, 135.4, 132.8, 131.6, 131.0, 128.5 (d, *J* ^3^_C‐F_ = 9.18 Hz), 122.0, 118.0, 116.0, 111.0 (d, *J* ^3^_C‐F_ = 9.21 Hz), 108.6, 75.5, 68.7, 67.7, 65.9, 54.9, 53.8, 51.5, 50.3, 49.8, 47.3, 47.2, 31.6, 31.1, 30.4; ESI‐HRMS (*m*/*z*) calculated for [M+H]^+^ ion species C_38_H_43_Cl_2_F_2_N_7_O_6_S 834.7618, found 834.7626; Elemental analysis, calculated: C, 54.68; H, 5.19; N, 11.75; found: C, 54.71; H, 5.17; N, 11.79.

### 4.2 In Vitro CA Inhibition Assay

The CA‐catalyzed CO_2_ hydration activity measurement was performed on an Applied Photophysics stopped‐flow instrument using phenol red, at a concentration of 0.2 mM, with a pH indicator with 20 mM HEPES (pH 7.5) as the buffer, 20 mM Na_2_SO_4_, and following the initial rates of the CA‐catalyzed CO_2_ hydration reaction for a period of 10–100 s and working at a maximum absorbance of 557 nm. The CO_2_ concentrations ranged from 1.7 to 17 mM. Enzyme concentrations varied between 5 and 12 nM [24, 25]. For each inhibitor, six traces of the initial 5%−10% of the reaction have been used to determine the initial velocity. The uncatalyzed reaction rates were determined in the same manner and subtracted from the total observed rates. Stock solutions of inhibitors (0.1 mM) were prepared in distilled H_2_O, and dilutions up to 0.01 nM were prepared. Solutions containing an inhibitor and an enzyme were preincubated for 15 min at room temperature before the assay to allow the formation of the E−I complex. The inhibition constants were obtained by nonlinear least‐squares methods using PRISM 3 and the Cheng–Prusoff equation as reported earlier [26], and represent the mean from at least three different determinations. All CAs were recombinant ones and were obtained in‐house [27, 28].

### 4.3 Antifungal Activity Evaluation Methods

All the fungal strains were stored at –80°C in cryovials, and then the inoculum preparations for broth microdilution antifungal assays were performed following the CLSI guidelines for antifungal susceptibility testing of yeasts, with some minor modifications (Clinical and Laboratory Standards Institute (CLSI) [29]. Briefly, yeasts *M. pachydermatis* DSMZ 6172 (CDC 16334) (MP), *M. furfur* ATCC 14521 (MF), and *M. globosa* ATCC MYA 4612 (MG) were inoculated into 6 mL of modified RPMI 1640 broth (Gibco, Life Technologies Limited, UK) with the addition of ingredients suggested by Rojas et al. and incubated at 37°C for 48 h for MP and MF, while MG was incubated at 35°C for 72 h [30]. The fungal inoculum was prepared by suspending in phosphate buffer (PB), 10 mM pH 7 four to five colonies of about 1 mm diameter. The fungal suspension was then adjusted to an optical density of 0.5 McFarland, to reach a final concentration in the wells of the plates of 5 × 105 CFU/mL. Tested compounds were dissolved in DMSO at 25.6 mg/mL, and then two‐fold dilutions of the tested compounds were performed to reach final concentrations ranging from 256 to 0.00390 µg/mL. KTZ (purchased from Sigma Aldrich, Saint Louis, MO USA) was dissolved in DMSO at a concentration of 25.6 mg/mL and tested at a final range of 32–0.008 µg/mL. Growth and sterility controls were performed. After 48 h of incubation at 37°C for MP and MF and at 35°C for 72 h for MG, a MIC reading was performed. Quality control strains (*C. albicans* ATCC 11006, American Type Culture Collection, Manassas, VA, USA, and *M. pachydermatis* DSM 6172, German Collection of Microorganisms and Cell Cultures GmbH, DSMZ, Braunschweig, DE) were added each day to check the accuracy of the drug dilutions and the reproducibility of the results. For each test, three experiments were performed, with three replicates each. The MIC value of each tested compound against each strain was calculated as the average value of replicates (µg/mL) ± standard deviation (SD).

### 4.4 Cell Viability Test

Compounds' viability was evaluated on Human Keratinocyte cell line (HaCat) cells (BS CL 168), purchased from Biobanking of Veterinary Resources of Istituto Zooprofilattico Sperimentale della Lombardia e dell'Emilia Romagna (Via Bianchi 9, 25124, Brescia, Italy). In a microtiter 96‐well plate, about 5 × 103 cells/mL per well were incubated for 12 h at 37°C in DMEM medium in a humidified atmosphere with 5% CO_2_ in the presence of 0.0625–8 μg/mL of the tested compounds and KTZ. Negative controls were performed both with 1% DMSO and with cells and DMEM only. After incubation, 10 μL of MTT (3‐(4,5‐dimethylthiazol‐2‐yl)‐2,5‐diphenyltetrazolium bromide) (Sigma Aldrich, Saint Louis, MO, USA) was added to each well and incubated at 37°C for 6 h. At the end of the incubation, 100 μL of the solubilization solution (10% SDS in 0.01 M HCl) was added to each well and then incubated for 12 h. The yellow tetrazolium MTT salt is reduced in metabolically active cells to form insoluble purple formazan crystals, which are solubilized by the addition of a detergent. After incubation, plates were read using a spectrophotometer, and the optical density was measured at 540 nm [31].

### 4.5 Sterol Analysis

#### 4.5.1 Compound Dilution

A volume of 1.0 mL of dimethyl sulfoxide (DMSO) was added to **9a–d**, **35a**, **38**, **49b**, **49c**, and the reference drug KTZ, which were serially diluted to 1, 100, and 10 µg/mL in Modified Dixon (DM) media and stored at –20°C. A volume of 1.0 L DM adjusted to pH 6 contained 36 g of malt extract, 20 g of desiccated Ox‐Bile, 6 g of peptone, 10 mL of Tween 40, 2 mL of glycerol, and 2 mL of oleic acid.

#### 4.5.2 Culturing of Wild‐Type *C. albicans*

*C. albicans* was cultured in 10 mL of YPD media in 60 mL pots at 30°C for 2 days. A volume of 1.0 L of YPD for agar plates contains 2% agar, 10 g of yeast extract, 20 g of peptone, and 20 g of glucose.

#### 4.5.3 Drug‐Treated Growth of *C. albicans*

Final concentrations of **9a‐d**, **35a**, **38**, **49b**, **49c,** and the reference drug KTZ were based on the MIC value for KTZ from previous work [21] and the MIC values for *M. globosa* reported in Table 2. The media were spiked with each drug to obtain the final concentrations and inoculated with 100 µL of culture to obtain 10 mL of culture. The cultures were incubated at 30°C with shaking for 2 days.

#### 4.5.4 Cells Pelleting From *C. albicans*

A total of 1.0 mL from each culture was transferred to 1.5 mL eppendorph tubes and centrifuged at 3000 rpm for 3 min. The supernatant was removed, and another 1 mL of culture was added and centrifuged at 3000 rpm. This procedure was repeated so that in total, 3 mL of culture was pelleted. The supernatant was removed, and the pellet was resuspended in deionized H_2_O, centrifuged at 3000 rpm for 3 min. This was repeated once more. The supernatant was removed, and the pellet was stored at −20°C until sterols were extracted.

#### 4.5.5 Sterol Extraction

Each pellet was resuspended in 1.0 mL of MeOH and transferred to glass test tubes. A volume of 3 mL of 60% w/v KOH and MeOH (1/1) was added. A volume of 1.0 mL of 0.5% pyrogallol was added; glass marbles were placed on top of the test tubes and covered with an aluminum foil. The sample was vortexed for 30 s to mix the components, heated in a water bath at 80°C for 2 h, and allowed to cool. Approximately 1.0 mL of *n*‐hexane was added to each tube, which was then vortexed for 30 s, and allowed to settle to form two layers. The top *n*‐hexane layer containing the sterols was removed and transferred to a 2 mL glass vial. This was repeated so that approximately 2 mL of the *n*‐hexane fraction was collected. *n*‐hexane was dried under a flow of nitrogen, leaving a dried residue of sterols, which was derivatized by adding 400 µL of pyridine and 100 µL of BSTFA, and heating at 70°C on a hot block for 1 h.

#### 4.5.6 GC‐MS Sterol Analysis

Trimethylsilyl (TMS)‐derivatized sterols were analyzed using a Thermo Trace 1300 GC system (Agilent Technologies) coupled to a Thermo ISQ LT‐MS unit and identified with reference to retention times and fragmentation spectra for known standards. A DB‐5MS fused silica column (30 m × 0.25 mm × 0.25 µm film thickness) was used for all GC separations (J&W Scientific). The initial oven temperature was held at 70°C for 4 min, followed by ramping (25°C/min) to a final temperature of 280°C; this temperature was held for 25 min. Samples were analyzed in splitless mode (1 L injection volume) using helium carrier gas and electron impact ionization (ion source temperature, 290°C) and scanning from 50 to 700 atomic mass units (amu). GC‐MS data files were analyzed using Agilent software (MSD Enhanced ChemStation) to generate sterol profiles and for the derivation of integrated peak areas.

## Conflicts of Interest

The authors declare no conflicts of interest.

## Supporting information


**Table S1:** Mass spectra of the main peaks (> 1% of the dominant peak) were compared with NIST database spectra to identify each component Compounds in green identified with probability < 20%; Compounds in yellow 2nd library hit identified with probability below 20%.

**Table S2:** The peak area of each identified peak was used to calculate their percentages in relation to the total peak areas of all identified peaks.

## Data Availability

The data that support the findings of this study are available from the corresponding author upon reasonable request.

## References

[1] R. Donato , C. Sacco , G. Pini , and A. R. Bilia , “Antifungal Activity of Different Essential Oils Against *Malassezia* Pathogenic Species,” Journal of Ethnopharmacology 249 (2020): 112376. 31704415 10.1016/j.jep.2019.112376

[2] WHO . WHO releases first‐ever list of health‐threatening fungi, Accessed February 18, 2025, https://www.who.int/news/item/25-10-2022-who-releases-first-ever-list-of-health-threatening-fungi. PMC10043918 36379529

[3] W. Rhimi , B. Theelen , T. Boekhout , D. Otranto , and C. Cafarchia , “Malassezia Spp. Yeasts of Emerging Concern in Fungemia,” Frontiers in Cellular and Infection Microbiology 10 (2020): 370. 32850475 10.3389/fcimb.2020.00370 PMC7399178

[4] R. Bond , E. A. Ferguson , C. F. Curtis , J. M. Craig , and D. H. Lloyd , “Factors Associated With Elevated Cutaneous Malassezia Pachydermatis Populations in Dogs With Pruritic Skin Disease,” Journal of Small Animal Practice 3 (1996): 103. 10.1111/j.1748-5827.1996.tb02353.x 8683952

[5] R. Bond , D. O. Morris , J. Guillot , et al., “Biology, Diagnosis and Treatment of Malassezia Dermatitis in Dogs and Cats: Clinical Consensus Guidelines of the World Association for Veterinary Dermatology,” Veterinary Dermatology 1 (2020): 75. 10.1111/vde.12834 31957203

[6] W. Rhimi , B. Theelen , T. Boekhout , C. I. Aneke , D. Otranto , and C. Cafarchia , “Conventional Therapy and New Antifungal Drugs Against Malassezia Infections,” Medical Mycology 59 (2021): 215–234. 33099634 10.1093/mmy/myaa087

[7] M. Billamboz and S. Jawhara , “Anti‐Malassezia Drug Candidates Based on Virulence Factors of Malassezia‐Associated Diseases,” Microorganisms 11 (2023): 2599. 37894257 10.3390/microorganisms11102599 PMC10609646

[8] R. Arakaki and B. Welles , “Ketoconazole Enantiomer for the Treatment of Diabetes Mellitus,” Expert Opinion on Investigational Drugs 19 (2010): 185–194. 20047506 10.1517/13543780903381411

[9] K. L. Kunze , W. L. Nelson , E. D. Kharasch , K. E. Thummel , and N. Isoherranen , “Stereochemical Aspects of Itraconazole Metabolism In Vitro and In Vivo,” Drug Metabolism and Disposition 34 (2006): 583–590. 16415110 10.1124/dmd.105.008508

[10] G. D. Brown , D. W. Denning , N. A. R. Gow , S. M. Levitz , M. G. Netea , and T. C. White , “Hidden Killers: Human Fungal Infections,” Science Translational Medicine 4 (2012): 165rv13. 10.1126/scitranslmed.3004404 23253612

[11] M. C. Fisher , N. J. Hawkins , D. Sanglard , and S. J. Gurr , “Worldwide Emergence of Resistance to Antifungal Drugs Challenges Human Health and Food Security,” Science 360 (2018): 739–742. 29773744 10.1126/science.aap7999

[12] H. N. Fones , M. C. Fisher , and S. J. Gurr , “Emerging Fungal Threats to Plants and Animals Challenge Agriculture and Ecosystem Resilience,” Microbiology Spectrum 5 (2017): 2. 10.1128/microbiolspec.funk-0027-2016 PMC11687465 28361733

[13] C. T. Supuran and C. Capasso , “A Highlight on the Inhibition of Fungal Carbonic Anhydrases as Drug Targets for the Antifungal Armamentarium,” International Journal of Molecular Sciences 22 (2021): 4324. 33919261 10.3390/ijms22094324 PMC8122340

[14] C. T. Supuran , “Emerging Role of Carbonic Anhydrase Inhibitors,” Clinical Science 135 (2021): 1233–1249. 34013961 10.1042/CS20210040

[15] A. Angeli , A. Velluzzi , S. Selleri , et al., “Seleno Containing Compounds as Potent and Selective Antifungal Agents,” ACS Infectious Diseases 9 (1905): 2022. 10.1021/acsinfecdis.2c00250 PMC9940851 35984421

[16] F. Carta , A. Angeli , S. Selleri , C. T. Supuran , C. S. Cabassi , and C. Spadini , “Carbamoselenoyl Derivatives as Anti‐Infective Agents,” WO, no. A1, 2023073634.

[17] C. Spadini , N. Mezzasalma , A. E. Odigie , et al., “Comparative Efficacy of Selenoureido Carbonic Anhydrase Inhibitors and Azole Antifungal Drugs Against Clinical Isolates of Malassezia Pachydermatis,” Veterinary Dermatology 36 (2025): 302–313. 40091275 10.1111/vde.13336 PMC12058572

[18] K. D'Ambrosio , A. Di Fiore , V. Alterio , et al., “Multiple Binding Modes of Inhibitors to Human Carbonic Anhydrases: An Update on the Design of Isoform‐Specific Modulators of Activity,” Chemical Reviews 125 (2025): 150–222. 39700306 10.1021/acs.chemrev.4c00278

[19] R. G. Khalifah , “The Carbon Dioxide Hydration Activity of Carbonic Anhydrase,” Journal of Biological Chemistry 246 (1971): 2561–2573. 4994926

[20] L. Cai , X. Qin , Z. Xu , et al., “Comparison of Cytotoxicity Evaluation of Anticancer Drugs Between Real‐Time Cell Analysis and CCK‐8 Method,” ACS Omega 4, no. 7 (2019): 12036–12042. 31460316 10.1021/acsomega.9b01142 PMC6682106

[21] C. M. Martel , J. E. Parker , O. Bader , et al., “Identification and Characterization of Four Azole‐Resistant erg3 Mutants of *Candida albicans* ,” Antimicrobial Agents and Chemotherapy 54 (2010): 4527–4533. 20733039 10.1128/AAC.00348-10 PMC2976150

[22] NIST Data. Accessed February 18, 2025, https://www.nist.gov/data.

[23] J. H. Van Tyle , “Ketoconazole; Mechanism of Action, Spectrum of Activity, Pharmacokinetics, Drug Interactions, Adverse Reactions and Therapeutic Use,” Pharmacotherapy 6 (1984): 343. 10.1002/j.1875-9114.1984.tb03398.x 6151171

[24] I. D'Agostino , S. Zara , S. Carradori , et al., “Antimalarial Agents Targeting Plasmodium Falciparum Carbonic Anhydrase: Towards Dual Acting Artesunate Hybrid Compounds,” Chem Med Chem 1 (2023): e202300267. 10.1002/cmdc.202300267 37697903

[25] C. Baroni , M. Bozdag , G. Renzi , et al., “X‐Ray Crystallographic and Kinetic Studies of Biguanide Containing Aryl Sulfonamides as Carbonic Anhydrase Inhibitors,” RSC Medicinal Chemistry 16 (2025): 1633–1640, 10.1039/d4md01018c. 39935522 PMC11809658

[26] C. Yung‐Chi and W. H. Prusoff , “Relationship Between the Inhibition Constant (K1) and the Concentration of Inhibitor Which Causes 50% Inhibition (I50) of an Enzymatic Reaction,” Biochemical Pharmacology 23 (1973): 3099. 10.1016/0006-2952(73)90196-2 4202581

[27] E. Berrino , B. Michelet , K. Vitse , et al., “Superacid‐Synthesized Fluorinated Diamines act as Selective hCA IV Inhibitors,” Journal of Medicinal Chemistry 67 (2024): 19460–19474. 39447020 10.1021/acs.jmedchem.4c01795

[28] J. T. Kilbile , S. B. Sapkal , G. Renzi , et al., “Lasamide Containing Sulfonylpiperazines as Effective Agents for the Management of Glaucoma Associated Symptoms,” Chem Med Chem 19 (2024): e202400601. 39319579 10.1002/cmdc.202400601 PMC11648825

[29] Clinical and Laboratory Standards Institute (CLSI) . Reference Method for Broth Dilution Antifungal Susceptibility Testing of Yeasts. 4th edition M27. Vol. M27. 950 West Valley Road, Suite 2500, Wayne, Pennsylvania 19087, USA; 2017.

[30] F. D. Rojas , M. A. Sosa , M. S. Fernandez , M. E. Cattana , S. B. Cordoba , and G. E. Giusiano , “Antifungal Susceptibility of Malassezia Furfur, Malassezia Sympodialis, and Malassezia Globosa to Azole Drugs and Amphotericin B Evaluated Using a Broth Microdilution Method,” Medical Mycology 52 (2014): 641–646. 24965946 10.1093/mmy/myu010

[31] G. Donofrio , V. Franceschi , A. Capocefalo , S. Cavirani , and I. M. Sheldon , “Bovine Endometrial Stromal Cells Display Osteogenic Properties,” Reproductive Biology and Endocrinology 6 (2008): 65. 19087287 10.1186/1477-7827-6-65 PMC2657796

