# Supplementary material for: Design and Synthesis of Ketoconazole Derivatives as Innovative Anti‐Infective Agents
Source: Arch Pharm (Weinheim). 2025 Jul 29;358(7):e70062. doi: 10.1002/ardp.70062 (PMC12304870; doi:10.1002/ardp.70062)
Supplement: Supplementary file 1 — Table S1: Mass spectra of the main peaks (> 1% of the dominant peak) were compared with NIST database spectra to identify each component Compounds in green identified with probability < 20%; Compounds in yellow 2nd library hit identified with probability below 20%. [file ARDP-358-e70062-s002.doc]

**Supplemental Material: Novel Compounds and Biological Screening Results**

**Title of Manuscript** Design and Synthesis of Ketoconazole derivatives as innovative anti-infective agents

**Authors** Gioele Renzi^1^, Andrea Angeli^1*^, Silvia Selleri^1^, Costanza Spadini^2^, Nicolo’ Mezzasalma^2^, Marcus T. Hull^3^, Steven L. Kelly^3^, Clemente Capasso^4^, Clotilde S. Cabassi^2^, Fabrizio Carta^1*^ and Claudiu T. Supuran^1^

**Affiliations**

^1^ Università degli Studi di Firenze, NEUROFARBA Department, Section of Pharmaceutical and Nutraceutical Sciences, Via Ugo Schiff 6, 50019 Sesto Fiorentino (Florence), Italy

^2^ University of Parma, Department of Veterinary Science, Via del Taglio 10, 43126 Parma, Italy.

^3^ Faculty of Medicine, Health and Life Science, Institute of Life Science, Swansea University, Swansea, United Kingdom.

^4^ Department of Biology, Agriculture and Food Sciences, Institute of Biosciences and Bioresources, 80131 Napoli, Italy.

**Corresponding author – full address**

* (A.A.), mail: andrea.angeli@unifi.it and (F.C.) mail: fabrizio.carta@unifi.it

| **Cmp.** | **InChI** | **Biological Activity** |
| --- | --- | --- |
| **7** | InChI=1S/C34H36Cl2N6O6S/c35-24-2-9-30(31(36)18-24)34(21-40-12-11-38-22-40)47-20-27(48-34)19-46-26-5-3-25(4-6-26)41-13-15-42(16-14-41)33(43)39-32-10-1-23-17-28(49(37,44)45)7-8-29(23)32/h2-9,11-12,17-18,22,27,32H,1,10,13-16,19-21H2,(H,39,43)(H2,37,44,45)/t27-,32+,34-/m0/s1 | *^a^(K*_i_, MgCA, MpaCA, MreCA, hCA I, hCA II, hCA IX, hCA XII): 69497, 513.7, 206.2, 5912, 5847, 4.4, 63.1  ^b^ MIC M. pachydermatis DSMZ 6172: 0.083 ± 0.04  ^b^ MIC M. furfur ATCC 14521: 4.0 ± 0  ^b^ MIC M. globosa ATCC MYA 4612: >256 ± 0 |
| **9a** | InChI=1S/C31H32Cl2N6O6S/c32-22-4-9-28(29(33)16-22)31(20-37-11-10-35-21-37)44-19-26(45-31)18-43-25-7-5-24(6-8-25)38-12-14-39(15-13-38)30(40)36-23-2-1-3-27(17-23)46(34,41)42/h1-11,16-17,21,26H,12-15,18-20H2,(H,36,40)(H2,34,41,42)/t26-,31-/m0/s1 | *^a^* (*K*_i_, MgCA, MpaCA, MreCA, hCA I, hCA II, hCA IX, hCA XII)  69497, 513.7, 206.2, 5912, 5847, 4.4, 63.1  ^b^ MIC M. pachydermatis DSMZ 6172: 3.6 ± 3.25  ^b^ MIC M. furfur ATCC 14521: 6.0 ± 2.31  ^b^ MIC M. globosa ATCC MYA 4612: ≤ 0.5 ± 0.00 |
| **9b** | InChI=1S/C31H32Cl2N6O6S/c32-22-1-10-28(29(33)17-22)31(20-37-12-11-35-21-37)44-19-26(45-31)18-43-25-6-4-24(5-7-25)38-13-15-39(16-14-38)30(40)36-23-2-8-27(9-3-23)46(34,41)42/h1-12,17,21,26H,13-16,18-20H2,(H,36,40)(H2,34,41,42)/t26-,31-/m0/s1 | *^a^* (*K*_i_, MgCA, MpaCA, MreCA, hCA I, hCA II, hCA IX, hCA XII)  5489, 516.7, 613.9, 337.6, 71.8, 32.9, 8.2  ^b^ MIC M. pachydermatis DSMZ 6172: ≤ 0.5 ± 0  ^b^ MIC M. furfur ATCC 14521: 5.0 ± 2.0  ^b^ MIC M. globosa ATCC MYA 4612: 2.0 ± 0 |
| **9c** | InChI=1S/C32H34Cl2N6O6S/c33-24-3-10-29(30(34)17-24)32(21-38-12-11-36-22-38)45-20-27(46-32)19-44-26-6-4-25(5-7-26)39-13-15-40(16-14-39)31(41)37-18-23-1-8-28(9-2-23)47(35,42)43/h1-12,17,22,27H,13-16,18-21H2,(H,37,41)(H2,35,42,43)/t27-,32-/m0/s1 | *^a^* (*K*_i_, MgCA, MpaCA, MreCA, hCA I, hCA II, hCA IX, hCA XII)  5633, 821.2, 683.9, 437.2, 58.9, 13.9, 6.4  ^b^ MIC M. pachydermatis DSMZ 6172: 0.6 ± 0.25  ^b^ MIC M. furfur ATCC 14521: 2.5 ± 1.0  ^b^ MIC M. globosa ATCC MYA 4612: 1.0 ± 0 |
| **9d** | InChI=1S/C33H36Cl2N6O6S/c34-25-3-10-30(31(35)19-25)33(22-39-14-13-37-23-39)46-21-28(47-33)20-45-27-6-4-26(5-7-27)40-15-17-41(18-16-40)32(42)38-12-11-24-1-8-29(9-2-24)48(36,43)44/h1-10,13-14,19,23,28H,11-12,15-18,20-22H2,(H,38,42)(H2,36,43,44)/t28-,33-/m0/s1 | *^a^* (*K*_i_, MgCA, MpaCA, MreCA, hCA I, hCA II, hCA IX, hCA XII)  3333, 291.6, 281.7, 84.0, 563.4, 6.0, 9.5  ^b^ MIC M. pachydermatis DSMZ 6172: 0.6 ± 0.25  ^b^ MIC M. furfur ATCC 14521: 7.0 ± 2.0  ^b^ MIC M. globosa ATCC MYA 4612: 2.0 ± 0 |
| **10a** | InChI=1S/C34H35Cl2N9O6S/c35-24-1-10-31(32(36)17-24)34(22-42-12-11-38-23-42)50-21-29(51-34)20-49-28-6-2-26(3-7-28)43-13-15-44(16-14-43)33(46)39-18-25-19-45(41-40-25)27-4-8-30(9-5-27)52(37,47)48/h1-12,17,19,23,29H,13-16,18,20-22H2,(H,39,46)(H2,37,47,48)/t29-,34-/m0/s1 | *^a^* (*K*_i_, MgCA, MpaCA, MreCA, hCA I, hCA II, hCA IX, hCA XII)  7578, 4202, 892.6, 870.2, 390.0, 38.0, 44.0  ^b^ MIC M. pachydermatis DSMZ 6172: 0.33 ± 0.14  ^b^ MIC M. furfur ATCC 14521: 256 ± 0  ^b^ MIC M. globosa ATCC MYA 4612: 26.7 ± 9.24 |
| **10b** | InChI=1S/C34H35Cl2N9O6S/c35-24-4-9-31(32(36)16-24)34(22-42-11-10-38-23-42)50-21-29(51-34)20-49-28-7-5-26(6-8-28)43-12-14-44(15-13-43)33(46)39-18-25-19-45(41-40-25)27-2-1-3-30(17-27)52(37,47)48/h1-11,16-17,19,23,29H,12-15,18,20-22H2,(H,39,46)(H2,37,47,48)/t29-,34-/m0/s1 | *^a^* (*K*_i_, MgCA, MpaCA, MreCA, hCA I, hCA II, hCA IX, hCA XII)  5045, 702.1, 360.6, 896.5, 533.9, 41.2, 7.4 |
| **24a** | InChI=1S/C32H34Cl2N6O5S2/c33-24-3-10-29(30(34)17-24)32(21-38-12-11-36-22-38)44-20-27(45-32)19-43-26-6-4-25(5-7-26)39-13-15-40(16-14-39)31(46)37-18-23-1-8-28(9-2-23)47(35,41)42/h1-12,17,22,27H,13-16,18-21H2,(H,37,46)(H2,35,41,42)/t27-,32-/m0/s1 | *^a^* (*K*_i_, MgCA, MpaCA, MreCA, hCA I, hCA II, hCA IX, hCA XII)  7000, 4944, 339.2, 862.9, 605.0, 47.7, 72.5  ^b^ MIC M. pachydermatis DSMZ 6172: ≤ 0.5 ± 0  ^b^ MIC M. furfur ATCC 14521: 4.0 ± 0  ^b^ MIC M. globosa ATCC MYA 4612: 3.0 ± 1.15 |
| **24b** | InChI=1S/C33H36Cl2N6O5S2/c34-25-3-10-30(31(35)19-25)33(22-39-14-13-37-23-39)45-21-28(46-33)20-44-27-6-4-26(5-7-27)40-15-17-41(18-16-40)32(47)38-12-11-24-1-8-29(9-2-24)48(36,42)43/h1-10,13-14,19,23,28H,11-12,15-18,20-22H2,(H,38,47)(H2,36,42,43)/t28-,33-/m0/s1 | *^a^* (*K*_i_, MgCA, MpaCA, MreCA, hCA I, hCA II, hCA IX, hCA XII)  6866, 841.7, 462.7, 871.7, 258.0, 48.7, 9.2  ^b^ MIC M. pachydermatis DSMZ 6172: ≤ 0.5 ± 0  ^b^ MIC M. furfur ATCC 14521: 4.0 ± 0  ^b^ MIC M. globosa ATCC MYA 4612: 2.0 ± 0 |
| **24c** | InChI=1S/C31H32Cl2N6O5S2/c32-22-4-9-28(29(33)16-22)31(20-37-11-10-35-21-37)43-19-26(44-31)18-42-25-7-5-24(6-8-25)38-12-14-39(15-13-38)30(45)36-23-2-1-3-27(17-23)46(34,40)41/h1-11,16-17,21,26H,12-15,18-20H2,(H,36,45)(H2,34,40,41)/t26-,31-/m0/s1 | *^a^* (*K*_i_, MgCA, MpaCA, MreCA, hCA I, hCA II, hCA IX, hCA XII)  8405, 604.2, 924.5, 328.2, 392.9, 40.1, 8.0  ^b^ MIC M. pachydermatis DSMZ 6172: ≤ 0.5 ± 0  ^b^ MIC M. furfur ATCC 14521: 10.0 ± 4.0  ^b^ MIC M. globosa ATCC MYA 4612: 1.0 ± 0 |
| **24d** | InChI=1S/C31H32Cl2N6O5S2/c32-22-1-10-28(29(33)17-22)31(20-37-12-11-35-21-37)43-19-26(44-31)18-42-25-6-4-24(5-7-25)38-13-15-39(16-14-38)30(45)36-23-2-8-27(9-3-23)46(34,40)41/h1-12,17,21,26H,13-16,18-20H2,(H,36,45)(H2,34,40,41)/t26-,31-/m0/s1 | *^a^* (*K*_i_, MgCA, MpaCA, MreCA, hCA I, hCA II, hCA IX, hCA XII)  4482, 610.9, 825.2, 861.0, 736.6, 4.4, 8.2  ^b^ MIC M. pachydermatis DSMZ 6172: ≤ 0.5 ± 0  ^b^ MIC M. furfur ATCC 14521: 4.0 ± 0  ^b^ MIC M. globosa ATCC MYA 4612: 2.0 ± 0 |
| **25** | InChI=1S/C39H39Cl2N7O6S2/c40-29-5-14-35(36(41)21-29)39(25-46-16-15-43-26-46)53-24-33(54-39)23-52-32-10-8-31(9-11-32)47-17-19-48(20-18-47)38(55)45-30-6-3-28(4-7-30)37(49)44-22-27-1-12-34(13-2-27)56(42,50)51/h1-16,21,26,33H,17-20,22-25H2,(H,44,49)(H,45,55)(H2,42,50,51)/t33-,39-/m0/s1 | *^a^* (*K*_i_, MgCA, MpaCA, MreCA, hCA I, hCA II, hCA IX, hCA XII)  6058, 930.7, 620.6, 871.5, 882.6, 41.0, 9.0  ^b^ MIC M. pachydermatis DSMZ 6172: 1.0 ± 0  ^b^ MIC M. furfur ATCC 14521: 16.0 ± 0  ^b^ MIC M. globosa ATCC MYA 4612: >256 ± 0 |
| **26** | InChI=1S/C39H41Cl2N7O7S3/c40-29-3-14-36(37(41)23-29)39(26-46-18-17-43-27-46)54-25-33(55-39)24-53-32-8-6-31(7-9-32)47-19-21-48(22-20-47)38(56)45-30-4-12-35(13-5-30)58(51,52)44-16-15-28-1-10-34(11-2-28)57(42,49)50/h1-14,17-18,23,27,33,44H,15-16,19-22,24-26H2,(H,45,56)(H2,42,49,50)/t33-,39-/m0/s1 | *^a^* (*K*_i_, MgCA, MpaCA, MreCA, hCA I, hCA II, hCA IX, hCA XII)  6027, 2390, 338.2, 58.3, 17.3, 151.8, 62.3  ^b^ MIC M. pachydermatis DSMZ 6172: 0.0625 ± 0  ^b^ MIC M. furfur ATCC 14521: 8.0 ± 0  ^b^ MIC M. globosa ATCC MYA 4612: >256 ± 0 |
| **28** | InChI=1S/C31H30Cl2N6O6S/c32-21-1-7-26(27(33)15-21)31(19-37-10-9-35-20-37)43-18-24(45-31)17-42-23-4-2-22(3-5-23)38-11-13-39(14-12-38)30-36-28-16-25(46(34,40)41)6-8-29(28)44-30/h1-10,15-16,20,24H,11-14,17-19H2,(H2,34,40,41)/t24-,31-/m0/s1 | *^a^* (*K*_i_, MgCA, MpaCA, MreCA, hCA I, hCA II, hCA IX, hCA XII)  4570, 380.9, 680.8, 944.0, 8.3, 28.3, 47.2  ^b^ MIC M. pachydermatis DSMZ 6172: 0.0078 ± 0  ^b^ MIC M. furfur ATCC 14521: 0.067± 0  ^b^ MIC M. globosa ATCC MYA 4612: >256 ± 0 |
| **35a** | InChI=1S/C38H38Cl2N6O6S/c39-29-5-14-35(36(40)21-29)38(25-45-16-15-42-26-45)51-24-33(52-38)23-50-32-10-8-31(9-11-32)46-19-17-44(18-20-46)22-27-1-3-28(4-2-27)37(47)43-30-6-12-34(13-7-30)53(41,48)49/h1-16,21,26,33H,17-20,22-25H2,(H,43,47)(H2,41,48,49)/t33-,38-/m0/s1 | *^a^* (*K*_i_, MgCA, MpaCA, MreCA, hCA I, hCA II, hCA IX, hCA XII)  4249, 803.3, 683.6, 352.5, 26.5, 46.8, 8.2  ^b^ MIC M. pachydermatis DSMZ 6172: 0.005 ± 0.002  ^b^ MIC M. furfur ATCC 14521: 2.0 ± 0  ^b^ MIC M. globosa ATCC MYA 4612: 128 ± 0 |
| **35b** | InChI=1S/C39H40Cl2N6O6S/c40-31-7-14-36(37(41)21-31)39(26-46-16-15-43-27-46)52-25-34(53-39)24-51-33-10-8-32(9-11-33)47-19-17-45(18-20-47)23-29-1-5-30(6-2-29)38(48)44-22-28-3-12-35(13-4-28)54(42,49)50/h1-16,21,27,34H,17-20,22-26H2,(H,44,48)(H2,42,49,50)/t34-,39-/m0/s1 | *^a^* (*K*_i_, MgCA, MpaCA, MreCA, hCA I, hCA II, hCA IX, hCA XII)  4604, 695.0, 701.7, 4767, 620.0, 368.6, 64.4  ^b^ MIC M. pachydermatis DSMZ 6172: 0.5 ± 0.43  ^b^ MIC M. furfur ATCC 14521: 4.0 ± 0  ^b^ MIC M. globosa ATCC MYA 4612: >256 ± 0 |
| **35c** | InChI=1S/C40H42Cl2N6O6S/c41-32-7-14-37(38(42)23-32)40(27-47-18-17-44-28-47)53-26-35(54-40)25-52-34-10-8-33(9-11-34)48-21-19-46(20-22-48)24-30-1-5-31(6-2-30)39(49)45-16-15-29-3-12-36(13-4-29)55(43,50)51/h1-14,17-18,23,28,35H,15-16,19-22,24-27H2,(H,45,49)(H2,43,50,51)/t35-,40-/m0/s1 | *^a^* (*K*_i_, MgCA, MpaCA, MreCA, hCA I, hCA II, hCA IX, hCA XII)  4473, 646.6, 318.3, 5515, 55.7, 47.9, 7.5  ^b^ MIC M. pachydermatis DSMZ 6172: 1.0 ± 0  ^b^ MIC M. furfur ATCC 14521: 2.0 ± 0  ^b^ MIC M. globosa ATCC MYA 4612: 128 ± 0 |
| **36a** | InChI=1S/C37H37Cl2N5O8S2/c38-28-3-14-35(36(39)21-28)37(25-43-16-15-41-26-43)50-24-32(51-37)23-49-30-6-4-29(5-7-30)44-19-17-42(18-20-44)22-27-1-10-34(11-2-27)54(47,48)52-31-8-12-33(13-9-31)53(40,45)46/h1-16,21,26,32H,17-20,22-25H2,(H2,40,45,46)/t32-,37-/m0/s1 | *^a^* (*K*_i_, MgCA, MpaCA, MreCA, hCA I, hCA II, hCA IX, hCA XII)  5429, 577.0, 288.2, 2662, 180.0, 311.5, 5.9  ^b^ MIC M. pachydermatis DSMZ 6172: 0.0625 ± 0  ^b^ MIC M. furfur ATCC 14521: 0.25 ± 0  ^b^ MIC M. globosa ATCC MYA 4612: >256 ± 0 |
| **36b** | InChI=1S/C38H40Cl2N6O7S2/c39-30-5-14-36(37(40)21-30)38(26-45-16-15-42-27-45)52-25-33(53-38)24-51-32-8-6-31(7-9-32)46-19-17-44(18-20-46)23-29-3-12-35(13-4-29)55(49,50)43-22-28-1-10-34(11-2-28)54(41,47)48/h1-16,21,27,33,43H,17-20,22-26H2,(H2,41,47,48)/t33-,38-/m0/s1 | *^a^* (*K*_i_, MgCA, MpaCA, MreCA, hCA I, hCA II, hCA IX, hCA XII)  4062, 559.7, 451.1, 3418, 1196, 41.9, 6.2  ^b^ MIC M. pachydermatis DSMZ 6172: 0.0625 ± 0  ^b^ MIC M. furfur ATCC 14521: 2.0 ± 0  ^b^ MIC M. globosa ATCC MYA 4612: 64 ± 0 |
| **36c** | InChI=1S/C39H42Cl2N6O7S2/c40-31-5-14-37(38(41)23-31)39(27-46-18-17-43-28-46)53-26-34(54-39)25-52-33-8-6-32(7-9-33)47-21-19-45(20-22-47)24-30-3-12-36(13-4-30)56(50,51)44-16-15-29-1-10-35(11-2-29)55(42,48)49/h1-14,17-18,23,28,34,44H,15-16,19-22,24-27H2,(H2,42,48,49)/t34-,39-/m0/s1 | *^a^* (*K*_i_, MgCA, MpaCA, MreCA, hCA I, hCA II, hCA IX, hCA XII)  6124, 677.9, 815.8, 2986, 938.8, 43.9, 8.1  ^b^ MIC M. pachydermatis DSMZ 6172: 0.0625 ± 0  ^b^ MIC M. furfur ATCC 14521: 2.0 ± 0  ^b^ MIC M. globosa ATCC MYA 4612: 16 ± 0 |
| **37a** | InChI=1S/C32H34Cl2N6O6S/c33-23-1-10-29(30(34)17-23)32(21-39-12-11-36-22-39)45-20-27(46-32)19-44-26-6-4-25(5-7-26)40-15-13-38(14-16-40)18-31(41)37-24-2-8-28(9-3-24)47(35,42)43/h1-12,17,22,27H,13-16,18-21H2,(H,37,41)(H2,35,42,43)/t27-,32-/m0/s1 | *^a^* (*K*_i_, MgCA, MpaCA, MreCA, hCA I, hCA II, hCA IX, hCA XII)  5344, 5931, 398.2, 497.4, 21.4, 346.3, 61.0  ^b^ MIC M. pachydermatis DSMZ 6172: 0.0625 ± 0  ^b^ MIC M. furfur ATCC 14521: 1.0 ± 0  ^b^ MIC M. globosa ATCC MYA 4612: 4 ± 0 |
| **37b** | InChI=1S/C33H36Cl2N6O6S/c34-24-1-10-30(31(35)19-24)33(22-40-14-12-37-23-40)46-21-28(47-33)20-45-27-6-4-26(5-7-27)41-17-15-39(16-18-41)13-11-32(42)38-25-2-8-29(9-3-25)48(36,43)44/h1-10,12,14,19,23,28H,11,13,15-18,20-22H2,(H,38,42)(H2,36,43,44)/t28-,33-/m0/s1 | *^a^* (*K*_i_, MgCA, MpaCA, MreCA, hCA I, hCA II, hCA IX, hCA XII)  6484, 891.7, 691.9, 9238, 798.6, 49.1, 9.1  ^b^ MIC M. pachydermatis DSMZ 6172: 0.0625 ± 0  ^b^ MIC M. furfur ATCC 14521: 4.0 ± 0  ^b^ MIC M. globosa ATCC MYA 4612: 3.3 ± 1.15 |
| **37c** | InChI=1S/C33H36Cl2N6O6S/c34-24-4-9-30(31(35)18-24)33(22-40-13-11-37-23-40)46-21-28(47-33)20-45-27-7-5-26(6-8-27)41-16-14-39(15-17-41)12-10-32(42)38-25-2-1-3-29(19-25)48(36,43)44/h1-9,11,13,18-19,23,28H,10,12,14-17,20-22H2,(H,38,42)(H2,36,43,44)/t28-,33-/m0/s1 | *^a^* (*K*_i_, MgCA, MpaCA, MreCA, hCA I, hCA II, hCA IX, hCA XII)  7435, 1927, 280.2, 4351, 92.6, 331.4, 9.4  ^b^ MIC M. pachydermatis DSMZ 6172: 0.031 ± 0  ^b^ MIC M. furfur ATCC 14521: 4.0 ± 0  ^b^ MIC M. globosa ATCC MYA 4612: 4.3 ± 3.5 |
| **38** | InChI=1S/C31H33Cl2N5O5S/c32-24-3-10-29(30(33)17-24)31(21-37-12-11-35-22-37)42-20-27(43-31)19-41-26-6-4-25(5-7-26)38-15-13-36(14-16-38)18-23-1-8-28(9-2-23)44(34,39)40/h1-12,17,22,27H,13-16,18-21H2,(H2,34,39,40)/t27-,31-/m0/s1 | *^a^* (*K*_i_, MgCA, MpaCA, MreCA, hCA I, hCA II, hCA IX, hCA XII)  5810, 553.8, 473.1, 382.0, 122.0, 224.8, 9.3  ^b^ MIC M. pachydermatis DSMZ 6172: 0.013 ± 0.004  ^b^ MIC M. furfur ATCC 14521: 0.25 ± 0  ^b^ MIC M. globosa ATCC MYA 4612: 13.3 ± 4.62 |
| **39** | InChI=1S/C32H34Cl2N6O7S/c33-22-1-7-27(28(34)15-22)32(20-39-10-9-36-21-39)46-19-25(47-32)18-45-24-4-2-23(3-5-24)40-13-11-38(12-14-40)17-31(42)37-29-16-26(48(35,43)44)6-8-30(29)41/h1-10,15-16,21,25,41H,11-14,17-20H2,(H,37,42)(H2,35,43,44)/t25-,32-/m0/s1 | *^a^* (*K*_i_, MgCA, MpaCA, MreCA, hCA I, hCA II, hCA IX, hCA XII)  5538, 627.8, 390.5, 817.8, 77.1, 76.0, 6.3  ^b^ MIC M. pachydermatis DSMZ 6172: 0.125 ± 0  ^b^ MIC M. furfur ATCC 14521: 2.0 ± 0  ^b^ MIC M. globosa ATCC MYA 4612: 128 ± 0 |
| **48a** | InChI=1S/C37H43Cl2N7O6S/c38-28-1-10-34(35(39)23-28)37(26-43-14-12-41-27-43)51-25-32(52-37)24-50-31-6-2-29(3-7-31)44-17-15-42(16-18-44)13-11-36(47)46-21-19-45(20-22-46)30-4-8-33(9-5-30)53(40,48)49/h1-10,12,14,23,27,32H,11,13,15-22,24-26H2,(H2,40,48,49)/t32-,37-/m0/s1 | *^a^* (*K*_i_, MgCA, MpaCA, MreCA, hCA I, hCA II, hCA IX, hCA XII)  5562, 679.4, 403.5, 82.1, 49.1, 30.6, 3.7  ^b^ MIC M. pachydermatis DSMZ 6172: 0.25 ± 0  ^b^ MIC M. furfur ATCC 14521: 8.0 ± 0  ^b^ MIC M. globosa ATCC MYA 4612: >256 ± 0 |
| **48b** | InChI=1S/C37H42Cl3N7O6S/c38-27-1-7-32(33(39)21-27)37(25-44-12-10-42-26-44)52-24-30(53-37)23-51-29-4-2-28(3-5-29)45-15-13-43(14-16-45)11-9-36(48)47-19-17-46(18-20-47)35-8-6-31(22-34(35)40)54(41,49)50/h1-8,10,12,21-22,26,30H,9,11,13-20,23-25H2,(H2,41,49,50)/t30-,37-/m0/s1 | *^a^* (*K*_i_, MgCA, MpaCA, MreCA, hCA I, hCA II, hCA IX, hCA XII)  6479, 830.4, 472.4, 769.7, 20.7, 48.4, 31.1  ^b^ MIC M. pachydermatis DSMZ 6172: 0.031 ± 0  ^b^ MIC M. furfur ATCC 14521: 4.0 ± 0  ^b^ MIC M. globosa ATCC MYA 4612: 8 ± 0 |
| **48c** | InChI=1S/C38H42Cl2F3N7O6S/c39-27-1-7-32(34(40)21-27)37(25-47-12-10-45-26-47)55-24-30(56-37)23-54-29-4-2-28(3-5-29)48-15-13-46(14-16-48)11-9-36(51)50-19-17-49(18-20-50)35-8-6-31(57(44,52)53)22-33(35)38(41,42)43/h1-8,10,12,21-22,26,30H,9,11,13-20,23-25H2,(H2,44,52,53)/t30-,37-/m0/s1 | *^a^* (*K*_i_, MgCA, MpaCA, MreCA, hCA I, hCA II, hCA IX, hCA XII)  7208, 721.5, 416.6, 835.8, 167.9, 445.7, 9.4  ^b^ MIC M. pachydermatis DSMZ 6172: 0.0625 ± 0  ^b^ MIC M. furfur ATCC 14521: 4.0 ± 0  ^b^ MIC M. globosa ATCC MYA 4612: 5.3± 2.3 |
| **48d** | InChI=1S/C37H42Cl2FN7O6S/c38-27-1-7-32(33(39)21-27)37(25-44-12-10-42-26-44)52-24-30(53-37)23-51-29-4-2-28(3-5-29)45-15-13-43(14-16-45)11-9-36(48)47-19-17-46(18-20-47)35-8-6-31(22-34(35)40)54(41,49)50/h1-8,10,12,21-22,26,30H,9,11,13-20,23-25H2,(H2,41,49,50)/t30-,37-/m0/s1 | *^a^* (*K*_i_, MgCA, MpaCA, MreCA, hCA I, hCA II, hCA IX, hCA XII)  8036, 630.3, 471.4, 363.9, 44.6, 306.3, 56.1  ^b^ MIC M. pachydermatis DSMZ 6172: 0.0625 ± 0  ^b^ MIC M. furfur ATCC 14521: 8.0 ± 0  ^b^ MIC M. globosa ATCC MYA 4612: 106.7 ± 36.9 |
| **49a** | InChI=1S/C38H45Cl2N7O6S/c39-29-2-11-35(36(40)24-29)38(27-44-17-13-42-28-44)52-26-33(53-38)25-51-32-7-3-30(4-8-32)46-20-18-43(19-21-46)16-12-37(48)47-15-1-14-45(22-23-47)31-5-9-34(10-6-31)54(41,49)50/h2-11,13,17,24,28,33H,1,12,14-16,18-23,25-27H2,(H2,41,49,50)/t33-,38-/m0/s1 | *^a^* (*K*_i_, MgCA, MpaCA, MreCA, hCA I, hCA II, hCA IX, hCA XII)  56446, 459.3, 410.9, 315.1, 288.5, 13.8, 5.5  ^b^ MIC M. pachydermatis DSMZ 6172: 0.0625 ± 0  ^b^ MIC M. furfur ATCC 14521: 10.7 ± 4.62  ^b^ MIC M. globosa ATCC MYA 4612: 13.3 ± 4.62 |
| **49b** | InChI=1S/C38H44Cl3N7O6S/c39-28-2-8-33(34(40)22-28)38(26-45-15-11-43-27-45)53-25-31(54-38)24-52-30-5-3-29(4-6-30)46-18-16-44(17-19-46)14-10-37(49)48-13-1-12-47(20-21-48)36-9-7-32(23-35(36)41)55(42,50)51/h2-9,11,15,22-23,27,31H,1,10,12-14,16-21,24-26H2,(H2,42,50,51)/t31-,38-/m0/s1 | *^a^* (*K*_i_, MgCA, MpaCA, MreCA, hCA I, hCA II, hCA IX, hCA XII)  6855, 733.0, 337.3, 96.1, 195.6, 272.1, 67.9  ^b^ MIC M. pachydermatis DSMZ 6172: 0.031 ± 0  ^b^ MIC M. furfur ATCC 14521: 2.0 ± 0  ^b^ MIC M. globosa ATCC MYA 4612: 4 ± 0 |
| **49c** | InChI=1S/C38H44Cl2FN7O6S/c39-28-2-8-33(34(40)22-28)38(26-45-15-11-43-27-45)53-25-31(54-38)24-52-30-5-3-29(4-6-30)46-18-16-44(17-19-46)14-10-37(49)48-13-1-12-47(20-21-48)36-9-7-32(23-35(36)41)55(42,50)51/h2-9,11,15,22-23,27,31H,1,10,12-14,16-21,24-26H2,(H2,42,50,51)/t31-,38-/m0/s1 | *^a^* (*K*_i_, MgCA, MpaCA, MreCA, hCA I, hCA II, hCA IX, hCA XII)  8184, 877.6, 470.2, 5.3, 7.2, 284.6, 7.9  ^b^ MIC M. pachydermatis DSMZ 6172: 0.031 ± 0  ^b^ MIC M. furfur ATCC 14521: 1.0 ± 0  ^b^ MIC M. globosa ATCC MYA 4612: >256 ± 0 |
| **49d** | InChI=1S/C38H43Cl2F2N7O6S/c39-27-2-7-32(33(40)20-27)38(25-46-13-9-44-26-46)54-24-30(55-38)23-53-29-5-3-28(4-6-29)47-16-14-45(15-17-47)12-8-36(50)48-10-1-11-49(19-18-48)37-34(41)21-31(22-35(37)42)56(43,51)52/h2-7,9,13,20-22,26,30H,1,8,10-12,14-19,23-25H2,(H2,43,51,52)/t30-,38-/m0/s1 | *^a^* (*K*_i_, MgCA, MpaCA, MreCA, hCA I, hCA II, hCA IX, hCA XII)  4391, 2583, 767.9, 777.0, 247.4, 2167, 55.9  ^b^ MIC M. pachydermatis DSMZ 6172: 0.05 ± 0.02  ^b^ MIC M. furfur ATCC 14521: 4.0 ± 0  ^b^ MIC M. globosa ATCC MYA 4612: >256 ± 0 |
| **AAZ** | InChI=1S/C4H5N3O4S2/c1-2(8)11-3-6-7-4(12-3)13(5,9)10/h1H3,(H2,5,9,10) | *^a^* (*K*_i_, MgCA, MpaCA, MreCA, hCA I, hCA II, hCA IX, hCA XII)  40000, 620.0, 100.0, 250.0, 12.1, 25.8, 5.7 |
|  |  |  |

^a^ ***In vitro* Carbonic Anhydrase inhibition assay**

The CA-catalyzed CO_2_ hydration activity measurement was performed on an Applied Photophysics stopped-flow instrument using phenol red, at a concentration of 0.2 mM, as a pH indicator with 20 mM HEPES (pH 7.5) as the buffer, 20 mM Na_2_SO_4_, and following the initial rates of the CA-catalyzed CO_2_ hydration reaction for a period of 10–100 s and working at the maximum absorbance of 557 nm. The CO_2_ concentrations ranged from 1.7 to 17 mM. Enzyme concentrations varied between 5 and 12 nM. ^1, 2^ For each inhibitor, six traces of the initial 5−10% of the reaction have been used to determine the initial velocity. The uncatalyzed reaction rates were determined in the same manner and subtracted from the total observed rates. Stock solutions of inhibitors (0.1 mM) were prepared in distilled H_2_O, and dilutions up to 0.01 nM were prepared. Solutions containing inhibitor and enzyme were preincubated for 15 min at room temperature before assay to allow the formation of the E−I complex. The inhibition constants were obtained by nonlinear least-squares methods using PRISM 3 and the Cheng–Prusoff equation as reported earlier ^3^ and represent the mean from at least three different determinations. All CAs were recombinant ones and were obtained in-house. ^4,5^

**^b^ Antifungal activity evaluation methods**

All the fungal strains were stored at -80 °C into cryovials, then the inoculum preparations for broth microdilution antifungal assays were performed following the CLSI guidelines for antifungal susceptibility testing of yeasts, with some minor modifications (Clinical and Laboratory Standards Institute (CLSI). ^6^ Briefly, the yeasts *M. pachydermatis* DSMZ 6172 (CDC 16334) (MP), *M. furfur* ATCC 14521 (MF) and *M. globosa* ATCC MYA 4612 (MG) were inoculated into 6 mL of modified RPMI 1640 broth (Gibco®, Life Technologies Limited, UK) with the addition of ingredients suggested by Rojas et al. and incubated at 37 °C for 48 hours for MP and MF, while MG was incubated at 35 °C for 72 hours. ^7^ The fungal inoculum was prepared by suspending in phosphate buffer (PB) 10 mM pH 7 four-five colonies of about 1 mm diameter. The fungal suspension was then adjusted to an optical density of 0.5 McFarland, to reach a final concentration into the wells of the plates of 5x10^5^ CFU/mL. Tested compounds were dissolved in DMSO at 25.6 mg/mL, then two-fold dilutions of the tested compounds were performed to reach final concentrations from 256 µg/mL to 0.00390 µg/mL. Ketoconazole (**KTZ**), (purchased from Sigma Aldrich®, Saint Louis, MO USA) was dissolved in DMSO at a concentration of 25.6 mg/mL and tested at a final range of 32-0.008 µg/ml. Growth and sterility controls were performed. After 48 hours of incubation at 37°C for MP and MF and 35°C for 72 hours for MG, MIC reading was performed. Quality control strains (*C. albicans* ATCC 11006, American Type Culture Collection, Manassas, VA, USA and *M. pachydermatis* DSM 6172, German Collection of Microorganisms and Cell Cultures GmbH, DSMZ, Braunschweig, DE) were included on each day to check the accuracy of the drug dilutions and the reproducibility of the results. For each test, three experiments were performed, with three replicates each. The MIC value of each tested compound against each strain was calculated as average value of replicates (µg/mL) ± standard deviation (SD).

**References**

1) D'Agostino, I.; Zara, S.; Carradori, S.; De Luca, V.; Capasso, C.; Kocken, C. H. M.; Zeeman, A.-M.; Angeli, A.; Carta, F.; Supuran, C. T. Antimalarial agents targeting Plasmodium Falciparum carbonic anhydrase: towards dual acting artesunate hybrid compounds. *Chem. Med. Chem*. **2023**, *1*, e202300267.

2) Baroni, C.; Bozdag, M.; Renzi, G.; De Luca, V.; Capasso, C.; Bazzicalupi, C.; Selleri, S.; Ferraroni, M.; Carta, F.; Supuran, C. T. X-Ray crystallographic and kinetic studies of biguanide containing aryl sulfonamides as Carbonic anhydrase inhibitors. *RSC Med. Chem*. **2025**. doi: 10.1039/d4md01018c.

3) Yung-Chi, C.; Prusoff, W. H. Relationship between the inhibition constant (K1) and the concentration of inhibitor which causes 50% inhibition (I50) of an enzymatic reaction. *Biochem. Pharmacol*. **1973**, *23*, 3099.

4) Berrino, E.; Michelet, B.; Vitse, K.; Nocentini, A.; Bartolucci, G.; Martin-Mingot, A.; Gratteri, P.; Carta, F.; Supuran, C. T.; Thibaudeau, S. Superacid-Synthesized Fluorinated Diamines Act as Selective hCA IV Inhibitors. *J. Med. Chem*. **2024**, *21*, 19460.

5) Kilbile, J. T., Sapkal, S. B., Renzi, G., D'Agostino, I., Boudjelal, M., Tamboli, Y.; Cutarella, L.; Mori, M.; Sgambellone, S.; Villano, S.; Marri, S.; Lucarini, L.; Carradori, S.; Carta, F.; Supuran, C. T. Lasamide Containing Sulfonylpiperazines as Effective Agents for the Management of Glaucoma Associated Symptoms. Chem. Med. Chem. 2024, 24, e202400601.

6) Clinical and Laboratory Standards Institute (CLSI). Reference method for broth dilution antifungal susceptibility testing of yeasts. 4th edition M27. Vol. M27. 950 West Valley Road, Suite 2500, Wayne, Pennsylvania 19087, USA; 2017.

7) Rojas, F. D.; De Los A. Sosa, M.; Fernández, M. S.; Cattana, M. E.; Córdoba, S. B.; Giusiano, G. E. Antifungal susceptibility of Malassezia furfur, Malassezia sympodialis, and Malassezia globosa to azole drugs and amphotericin B evaluated using a broth microdilution method. *Med. Mycol*. **2014**, *6*, 641.
