# Supplementary material for: Design and Synthesis of Ketoconazole Derivatives as Innovative Anti‐Infective Agents
Source: Arch Pharm (Weinheim). 2025 Jul 29;358(7):e70062. doi: 10.1002/ardp.70062 (PMC12304870; doi:10.1002/ardp.70062)
Supplement: Supplementary file 2 — Table S2: The peak area of each identified peak was used to calculate their percentages in relation to the total peak areas of all identified peaks. [file ARDP-358-e70062-s001.docx]

Supplementary Material for

**Design and Synthesis of Ketoconazole derivatives as innovative anti-infective agents**

Gioele Renzi^1^, Andrea Angeli^1*^, Silvia Selleri^1^, Costanza Spadini^2^, Nicolo’ Mezzasalma^2^, Marcus T. Hull^3^, Steven L. Kelly^3^, Clemente Capasso^4^, Clotilde S. Cabassi^2^, Fabrizio Carta^1*^ and Claudiu T. Supuran

^1^ Università degli Studi di Firenze, NEUROFARBA Department, Section of Pharmaceutical and Nutraceutical Sciences, Via Ugo Schiff 6, 50019 Sesto Fiorentino (Florence), Italy.

^2^ Department of Veterinary Science, University of Parma, via del Taglio 10, 43126 Parma, Italy.

^3^ Faculty of Medicine, Health and Life Science, Institute of Life Science, Swansea University, Swansea, United Kingdom.

^4^ Department of Biology, Agriculture and Food Sciences, Institute of Biosciences and Bioresources, 80131 Napoli, Italy.

*Corresponding authors: (A.A), mail: andrea.angeli@unifi.it and (F.C.) mail: fab-rizio.carta@unifi.it

Index

^1^H, ^13^C, Spectra of final compounds S2-S34

^1^H, ^13^C, Spectra of intermediate compounds S35-S55

Synthetic procedures and characterization of intermediates S56-S77

Mass spectra of the main peaks (**Table S1**) S78

Peak areas and percentage peak areas (**Table S2**) S79

References S81

**Copies of NMR Spectra of final compounds**

^1^H NMR spectrum of compound **7** (400 MHz, DMSO-*d_6_*)

^13^C NMR spectrum of compound **7** (100 MHz, DMSO-*d_6_*)

^1^H NMR spectrum of compound **9a** (400 MHz, DMSO-*d_6_*)


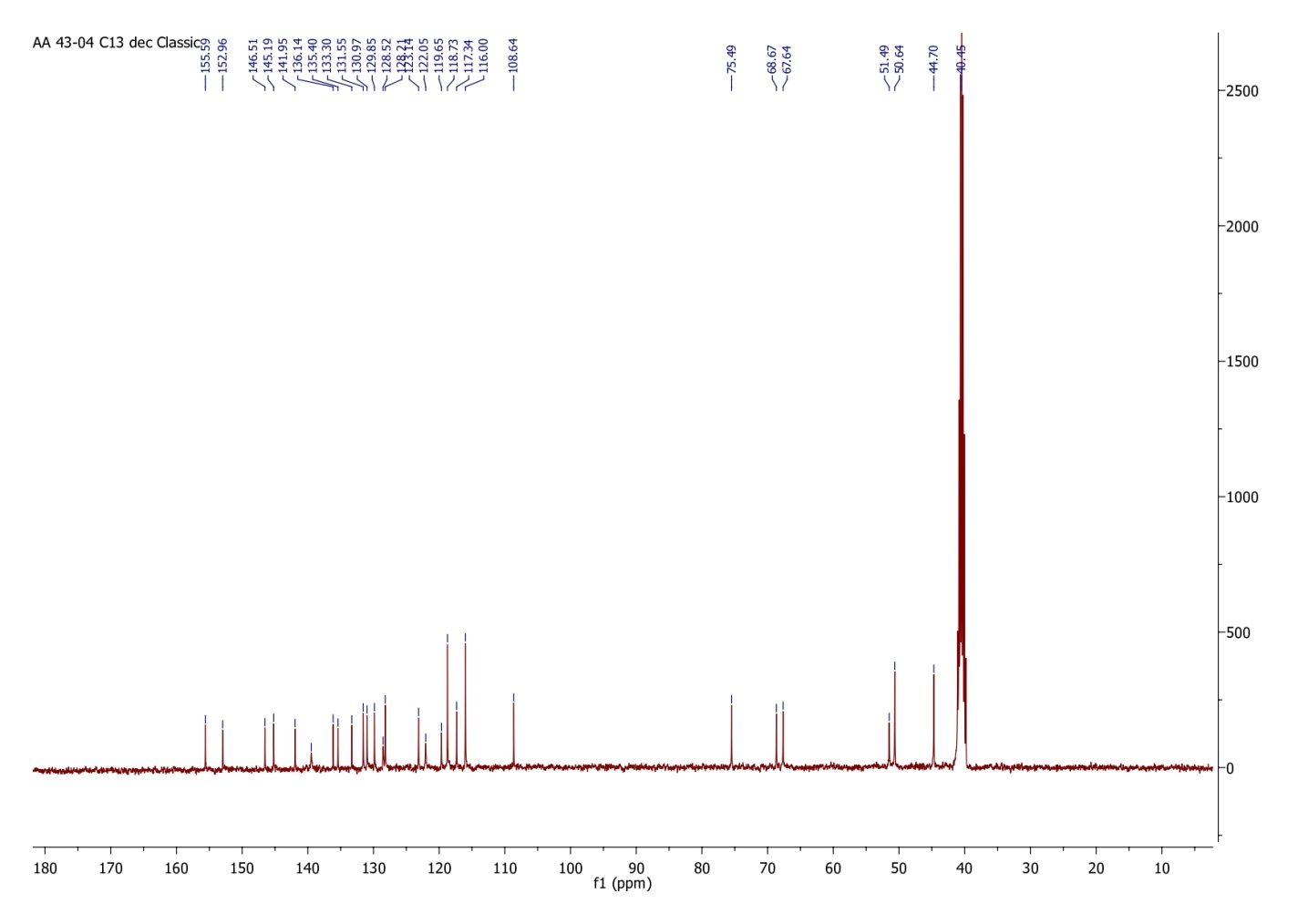


^13^C NMR spectrum of compound **9a** (100 MHz, DMSO-*d_6_*)

^1^H NMR spectrum of compound **9b** (400 MHz, DMSO-*d_6_*)


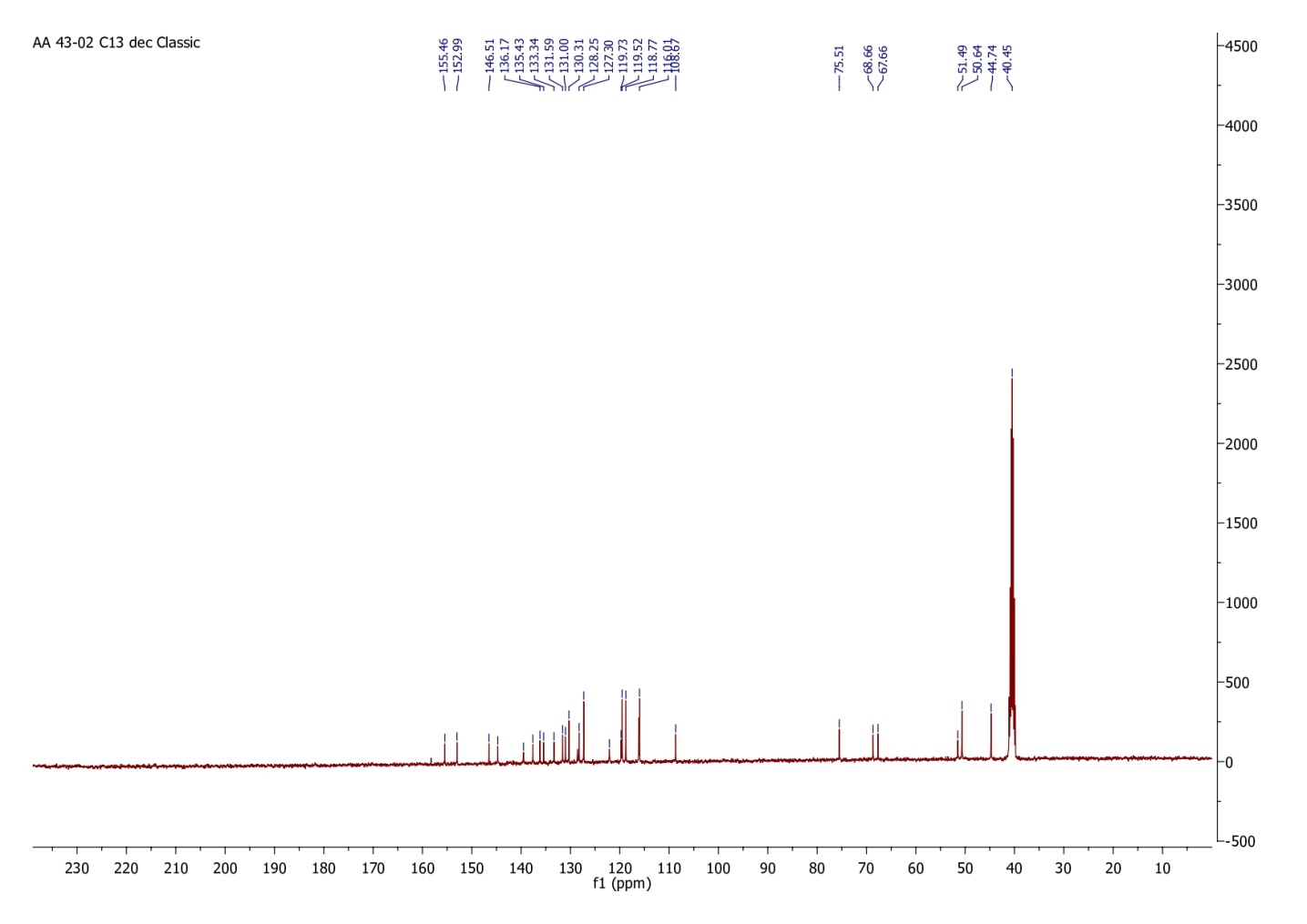


^13^C NMR spectrum of compound **9b** (100 MHz, DMSO-*d_6_*)

^1^H NMR spectrum of compound **9c** (400 MHz, DMSO-*d_6_*)


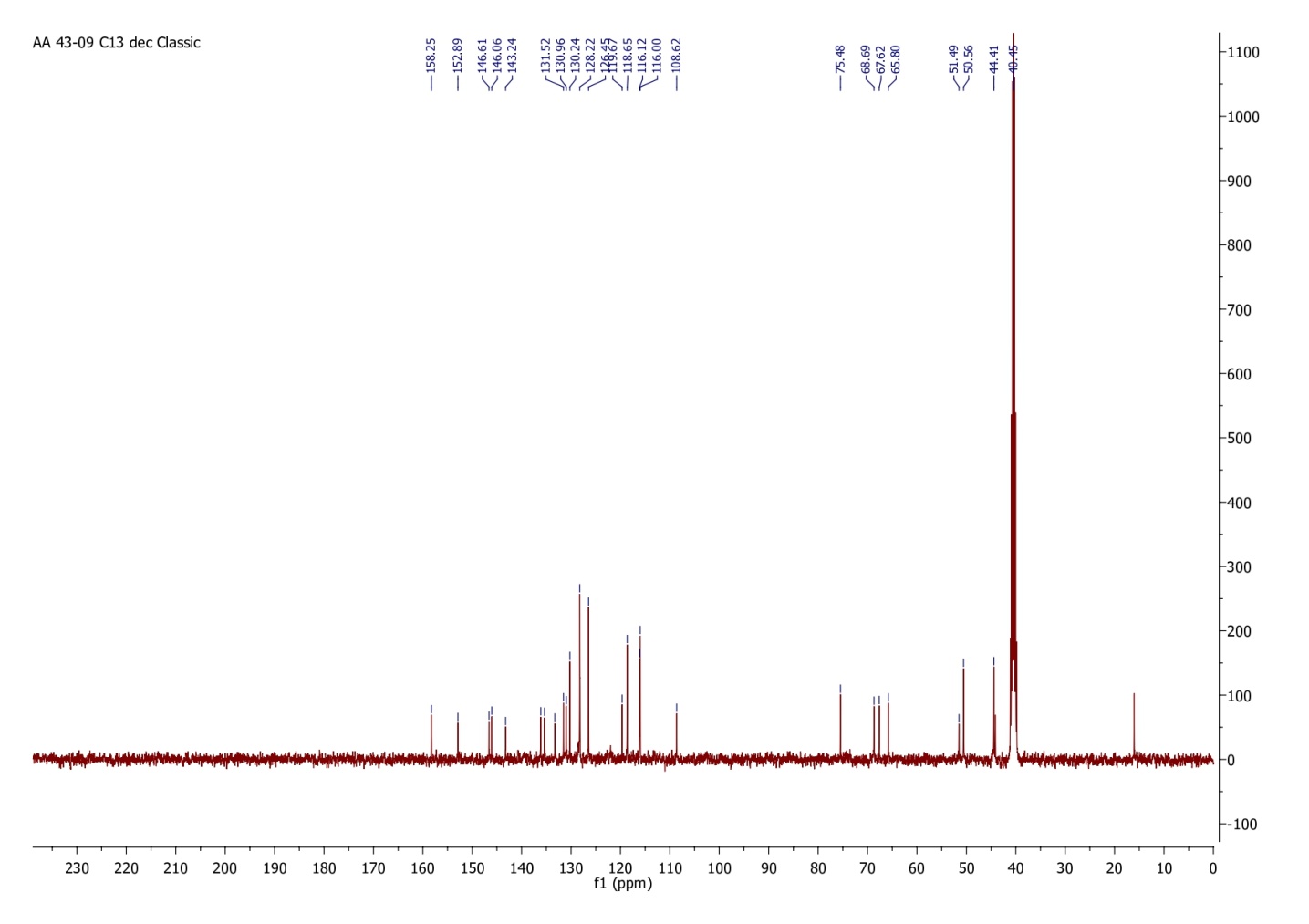


^13^C NMR spectrum of compound **9c** (100 MHz, DMSO-*d_6_*)

^1^H NMR spectrum of compound **9d** (400 MHz, DMSO-*d_6_*)


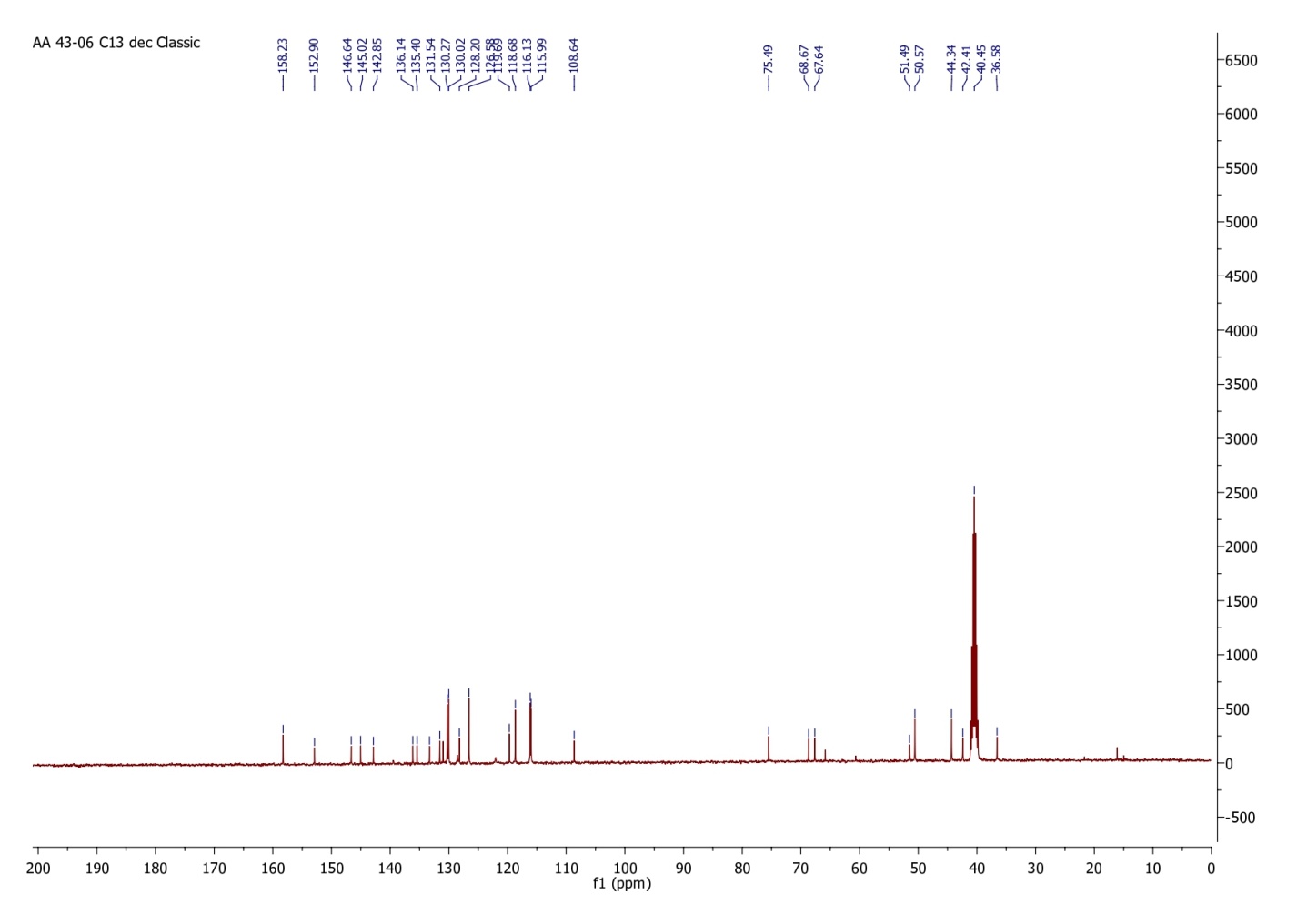


^13^C NMR spectrum of compound **9d** (100 MHz, DMSO-*d_6_*)

^1^H NMR spectrum of compound **10a** (400 MHz, DMSO-*d_6_*)

^13^C NMR spectrum of compound **10a** (100 MHz, DMSO-*d_6_*)

^1^H NMR spectrum of compound **10b** (400 MHz, DMSO-*d_6_*)

^13^C NMR spectrum of compound **10b** (100 MHz, DMSO-*d_6_*)

^1^H NMR spectrum of compound **24a** (400 MHz, DMSO-*d_6_*)


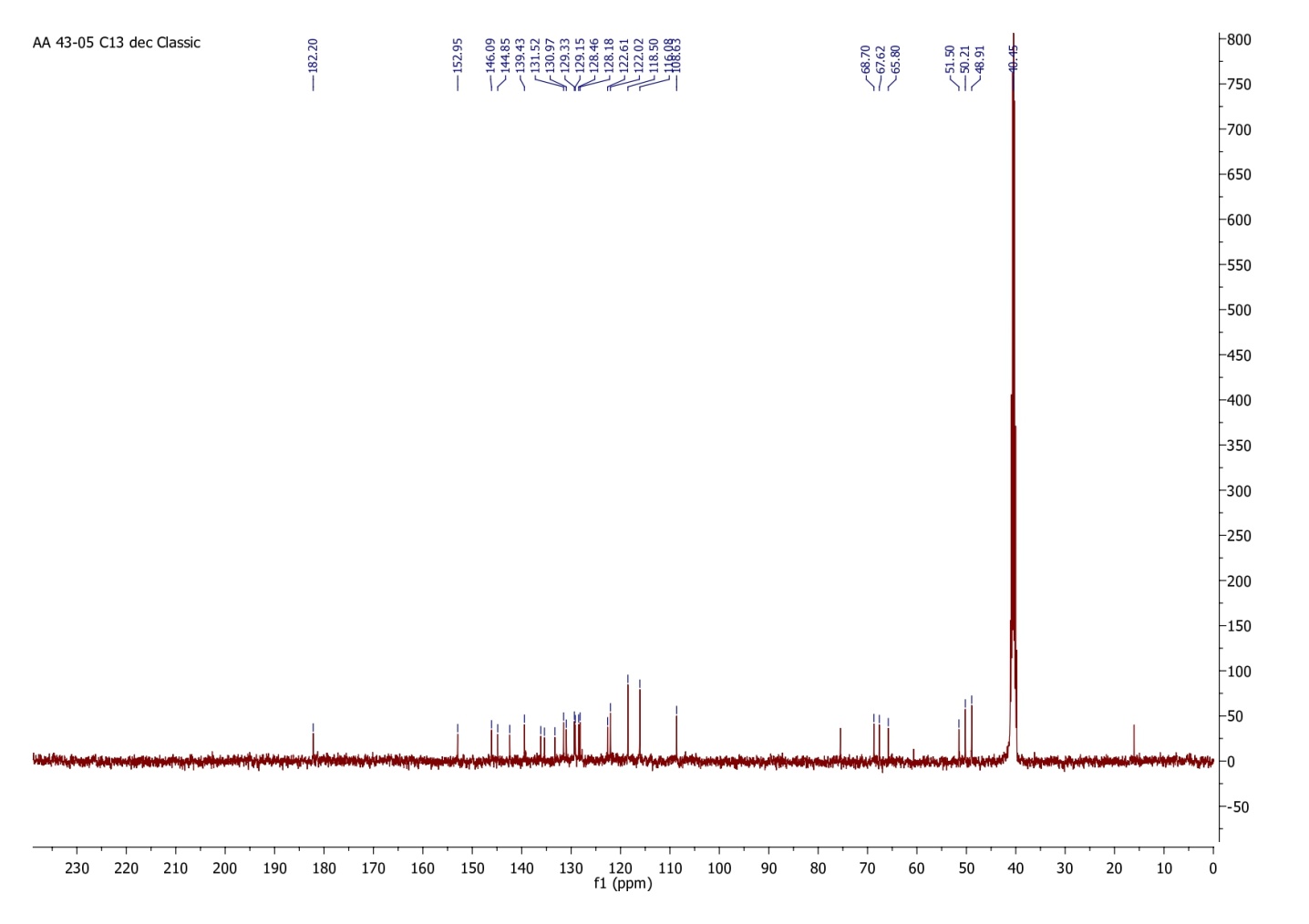


^13^C NMR spectrum of compound **24a** (100 MHz, DMSO-*d_6_*)

^1^H NMR spectrum of compound **24b** (400 MHz, DMSO-*d_6_*)


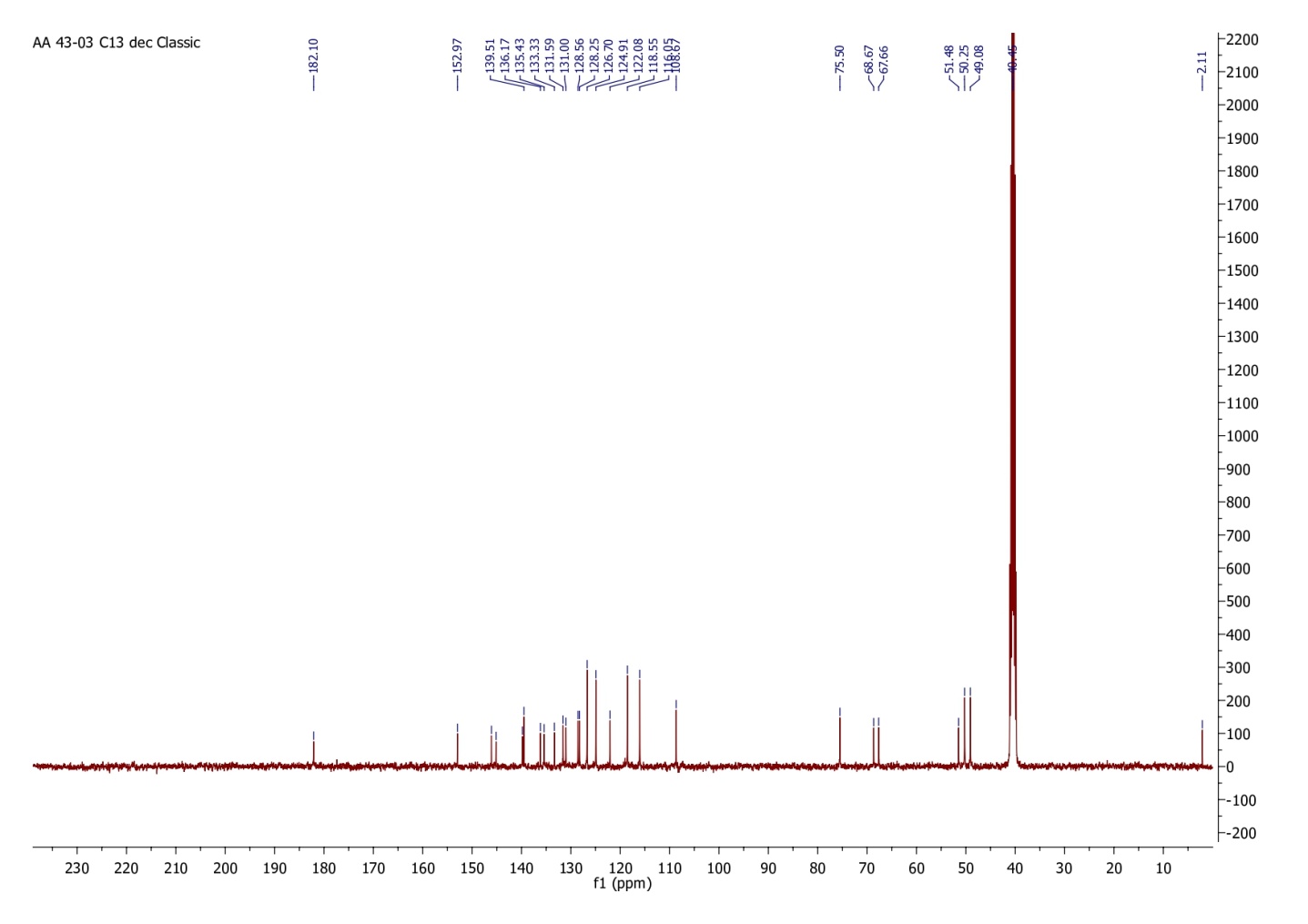


^13^C NMR spectrum of compound **24b** (100 MHz, DMSO-*d_6_*)

^1^H NMR spectrum of compound **24c** (400 MHz, DMSO-*d_6_*)


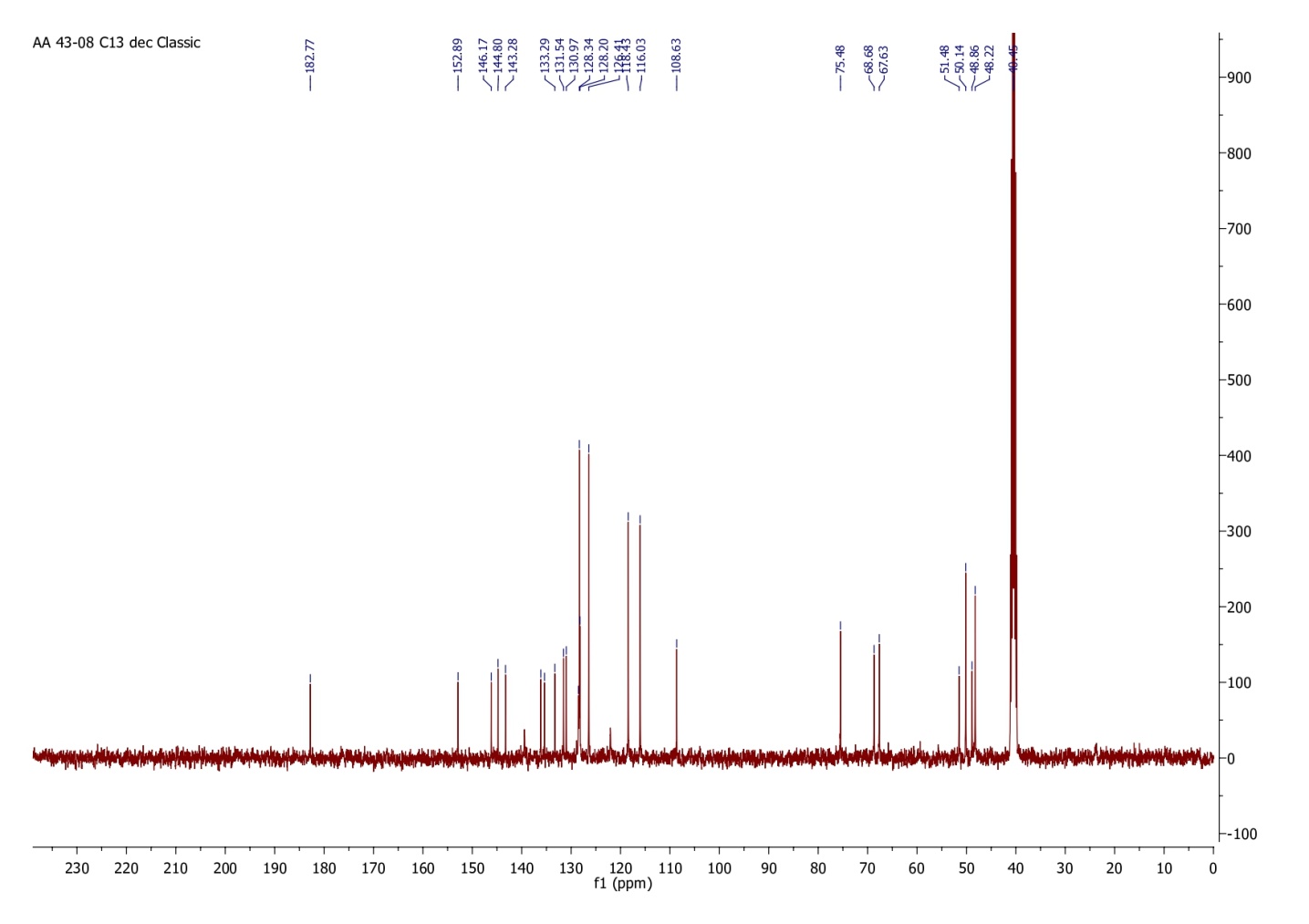


^13^C NMR spectrum of compound **24c** (100 MHz, DMSO-*d_6_*)

^1^H NMR spectrum of compound **24d** (400 MHz, DMSO-*d_6_*)


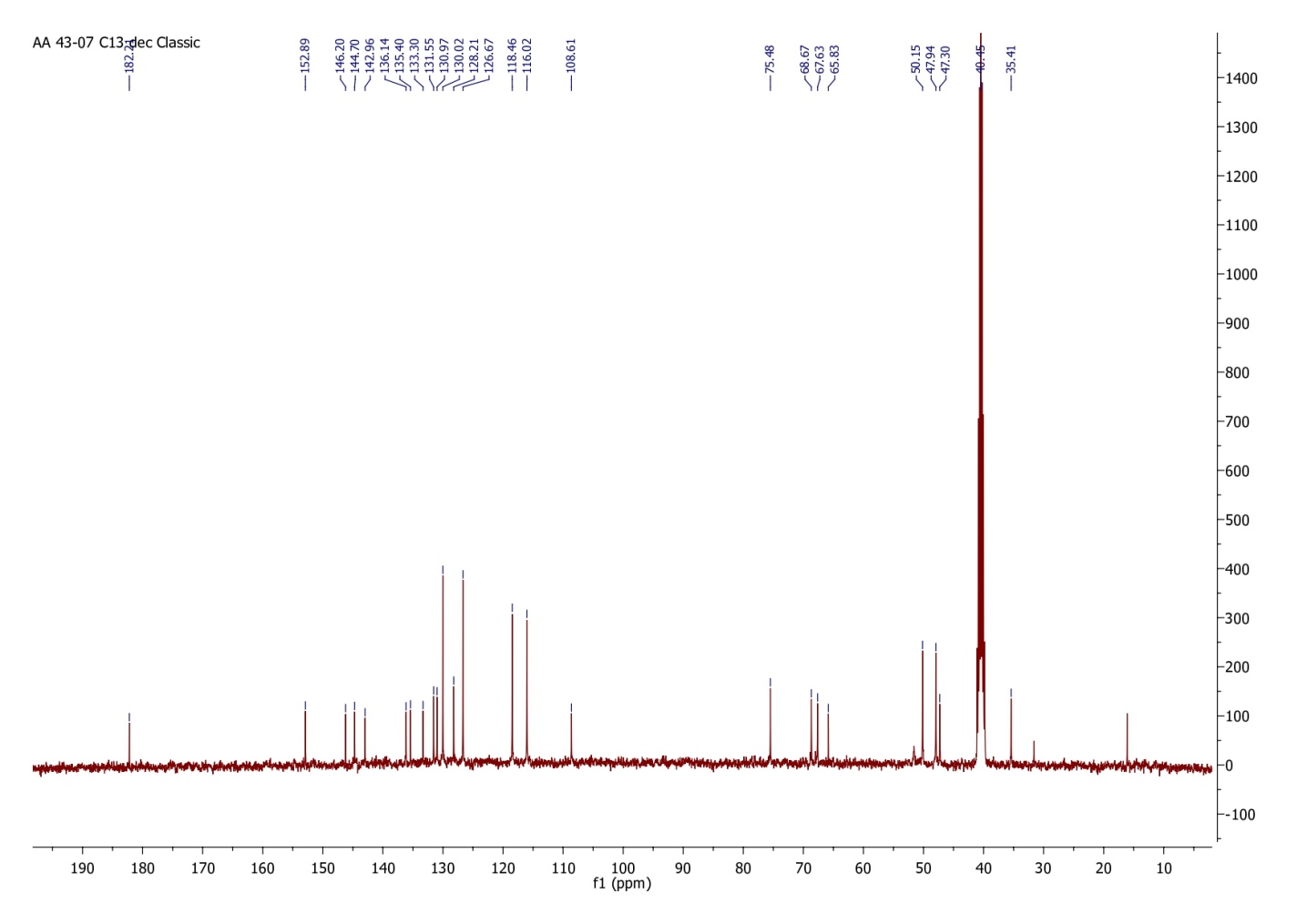


^13^C NMR spectrum of compound **24d** (100 MHz, DMSO-*d_6_*)

^1^H NMR spectrum of compound **25** (400 MHz, DMSO-*d_6_*)

^13^C NMR spectrum of compound **25** (100 MHz, DMSO-*d_6_*)

^1^H NMR spectrum of compound **26** (400 MHz, DMSO-*d_6_*)

^13^C NMR spectrum of compound **26** (100 MHz, DMSO-*d_6_*)

^1^H NMR spectrum of compound **28** (400 MHz, DMSO-*d_6_*)

^13^C NMR spectrum of compound **28** (100 MHz, DMSO-*d_6_*)

^1^H NMR spectrum of compound **35a** (400 MHz, DMSO-*d_6_*)

^13^C NMR spectrum of compound **35a** (100 MHz, DMSO-*d_6_*)

^1^H NMR spectrum of compound **35b** (400 MHz, DMSO-*d_6_*)

^13^C NMR spectrum of compound **35b** (100 MHz, DMSO-*d_6_*)

^1^H NMR spectrum of compound **35c** (400 MHz, DMSO-*d_6_*)

^13^C NMR spectrum of compound **35c** (100 MHz, DMSO-*d_6_*)

^1^H NMR spectrum of compound **36a** (400 MHz, DMSO-*d_6_*)

^13^C NMR spectrum of compound **36a** (100 MHz, DMSO-*d_6_*)

^1^H NMR spectrum of compound **36b** (400 MHz, DMSO-*d_6_*)

^13^C NMR spectrum of compound **36b** (100 MHz, DMSO-*d_6_*)

^1^H NMR spectrum of compound **36c** (400 MHz, DMSO-*d_6_*)

^13^C NMR spectrum of compound **36c** (100 MHz, DMSO-*d_6_*)

^1^H NMR spectrum of compound **37a** (400 MHz, DMSO-*d_6_*)

^13^C NMR spectrum of compound **37a** (100 MHz, DMSO-*d_6_*)

^1^H NMR spectrum of compound **37b** (400 MHz, DMSO-*d_6_*)

^13^C NMR spectrum of compound **37b** (100 MHz, DMSO-*d_6_*)

^1^H NMR spectrum of compound **37c** (400 MHz, DMSO-*d_6_*)

^13^C NMR spectrum of compound **37c** (100 MHz, DMSO-*d_6_*)

^1^H NMR spectrum of compound **38** (400 MHz, DMSO-*d_6_*)

^13^C NMR spectrum of compound **38** (100 MHz, DMSO-*d_6_*)

^1^H NMR spectrum of compound **39** (400 MHz, DMSO-*d_6_*)

^13^C NMR spectrum of compound **39** (100 MHz, DMSO-*d_6_*)

^1^H NMR spectrum of compound **48a** (400 MHz, DMSO-*d_6_*)

^13^C NMR spectrum of compound **48a** (100 MHz, DMSO-*d_6_*)

^1^H NMR spectrum of compound **48b** (400 MHz, DMSO-*d_6_*)

^13^C NMR spectrum of compound **48b** (100 MHz, DMSO-*d_6_*)

^
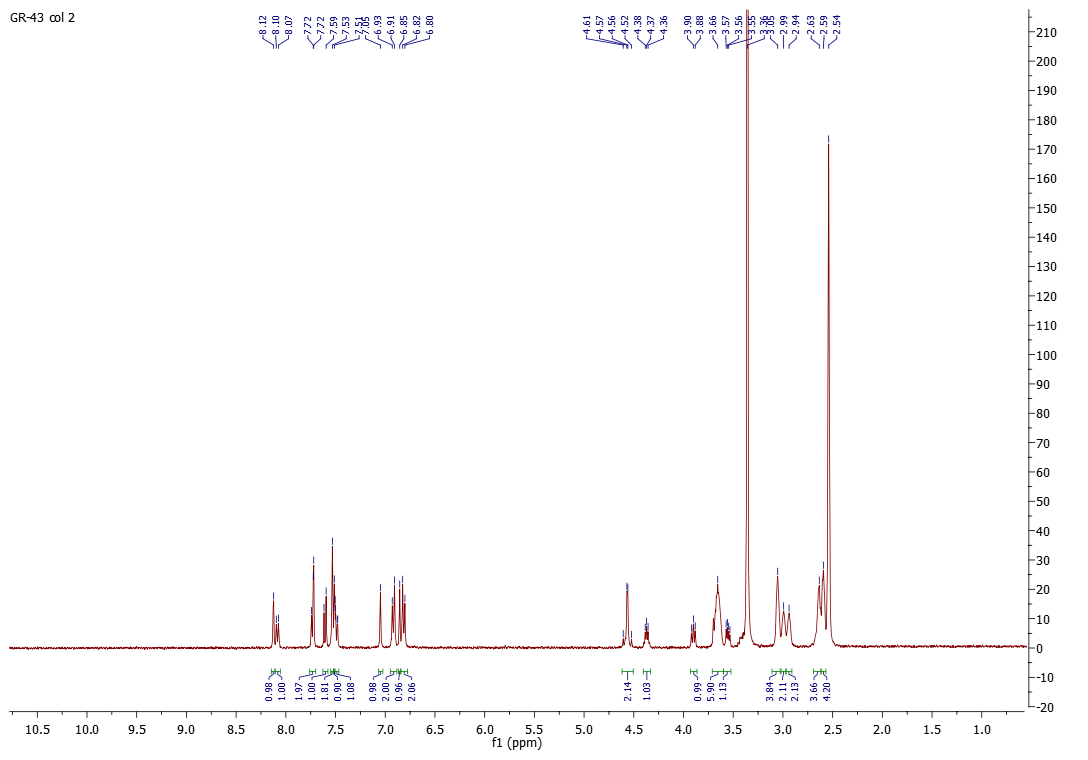
^

^1^H NMR spectrum of compound **48c** (400 MHz, DMSO-*d6*)


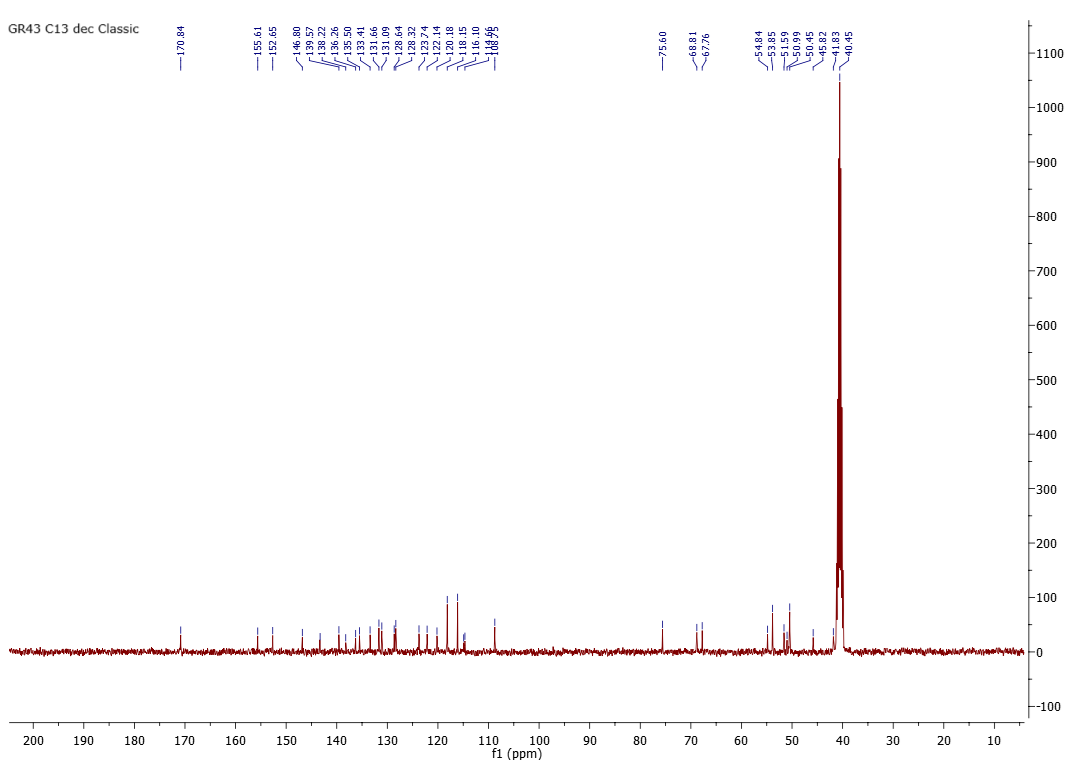


^13^C NMR spectrum of compound **48c** (100 MHz, DMSO-*d6*)

^1^H NMR spectrum of compound **48d** (400 MHz, DMSO-*d_6_*)

^13^C NMR spectrum of compound **48d** (100 MHz, DMSO-*d_6_*)

^1^H NMR spectrum of compound **49a** (400 MHz, DMSO-*d_6_*)

^13^C NMR spectrum of compound **49a** (100 MHz, DMSO-*d_6_*)

^1^H NMR spectrum of compound **49b** (400 MHz, DMSO-*d_6_*)

^13^C NMR spectrum of compound **49b** (100 MHz, DMSO-*d_6_*)

^1^H NMR spectrum of compound **49c** (400 MHz, DMSO-*d_6_*)

^13^C NMR spectrum of compound **49c** (100 MHz, DMSO-*d_6_*)

^1^H NMR spectrum of compound **49d** (400 MHz, DMSO-*d_6_*)

^13^C NMR spectrum of compound **49d** (100 MHz, DMSO-*d_6_*)

**Intermediates**

^1^H NMR spectrum of compound **45a** (400 MHz, DMSO-*d_6_*)


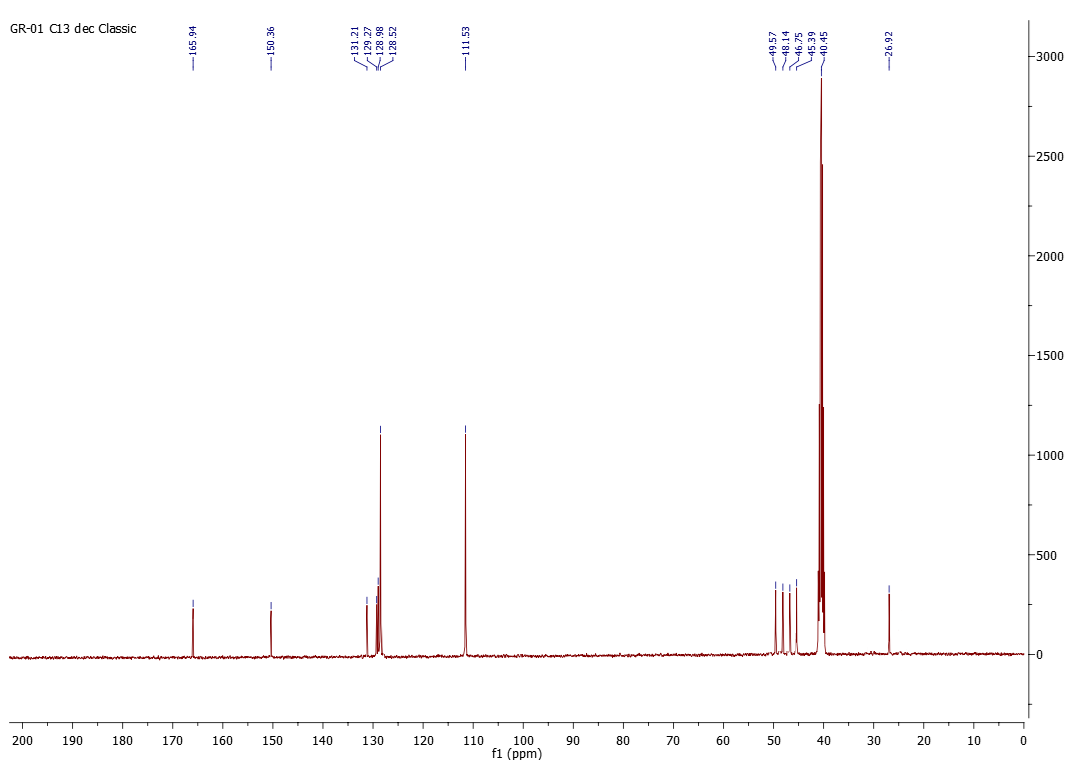


^13^C NMR spectrum of compound **45a** (100 MHz, DMSO-*d_6_*)

^1^H NMR spectrum of compound **3** (400 MHz, DMSO-*d_6_*)

^13^C NMR spectrum of compound **3** (100 MHz, DMSO-*d_6_*)

^1^H NMR spectrum of compound **44a** (400 MHz, DMSO-*d_6_*)

^13^C NMR spectrum of compound **44a** (100 MHz, DMSO-*d_6_*)

^1^H NMR spectrum of compound **45c** (400 MHz, DMSO-*d_6_*)


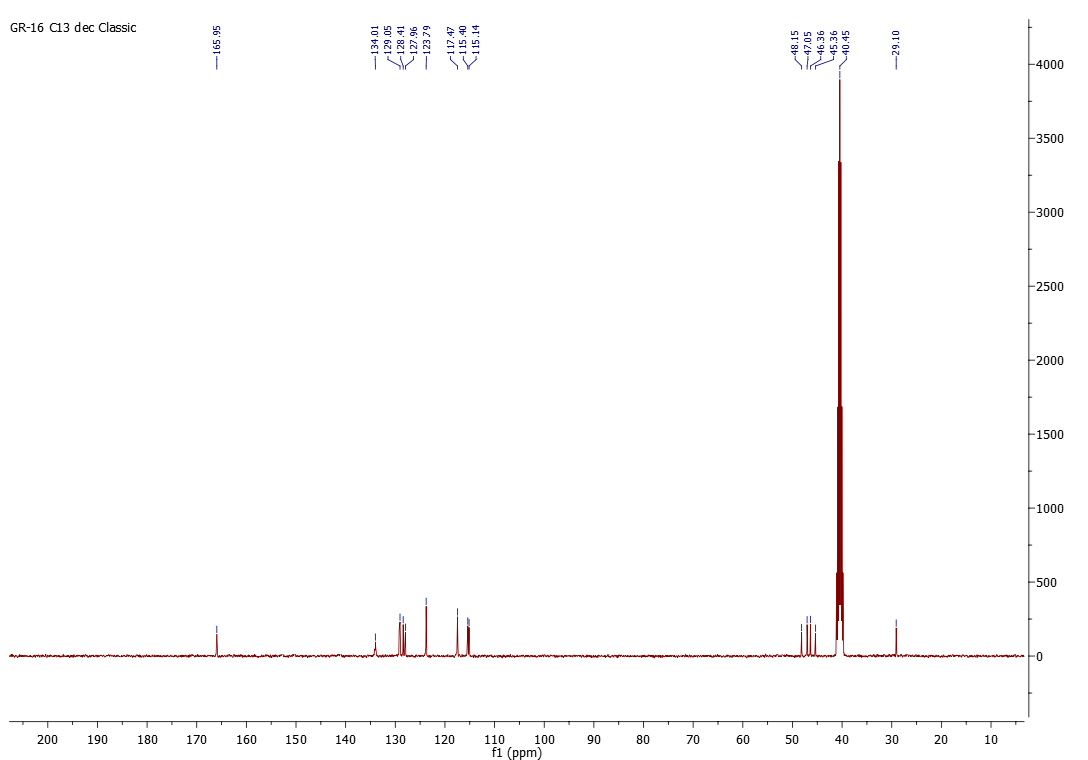


^13^C NMR spectrum of compound **45c** (100 MHz, DMSO-*d_6_*)

^1^H NMR spectrum of compound **45d** (400 MHz, DMSO-*d_6_*)


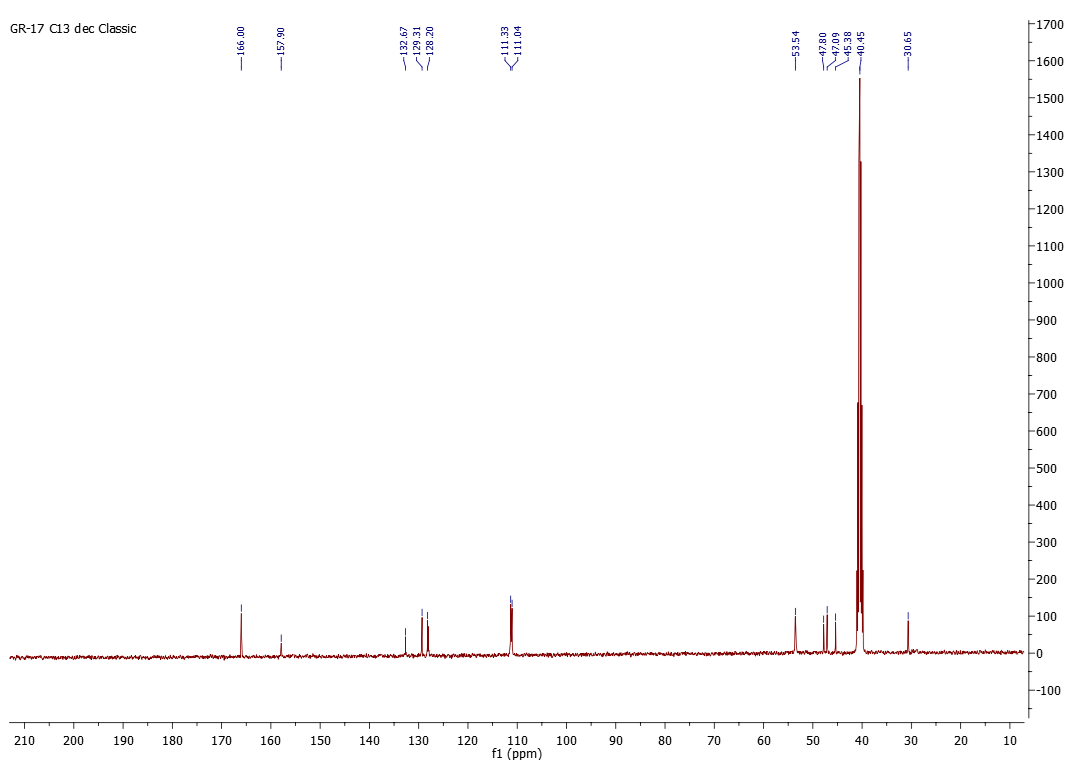


^13^C NMR spectrum of compound **45d** (100 MHz, DMSO-*d_6_*)

^1^H NMR spectrum of compound **46** (400 MHz, DMSO-*d_6_*)

^13^C NMR spectrum of compound **46** (100 MHz, DMSO-*d_6_*)

^1^H NMR spectrum of compound **4a** (400 MHz, DMSO-*d_6_*)

^13^C NMR spectrum of compound **4a** (100 MHz, DMSO-*d_6_*)

^1^H NMR spectrum of compound **43a** (400 MHz, DMSO-*d_6_*)

^13^C NMR spectrum of compound **43a** (100 MHz, DMSO-*d_6_*)

^1^H NMR spectrum of compound **45b** (400 MHz, DMSO-*d_6_*)


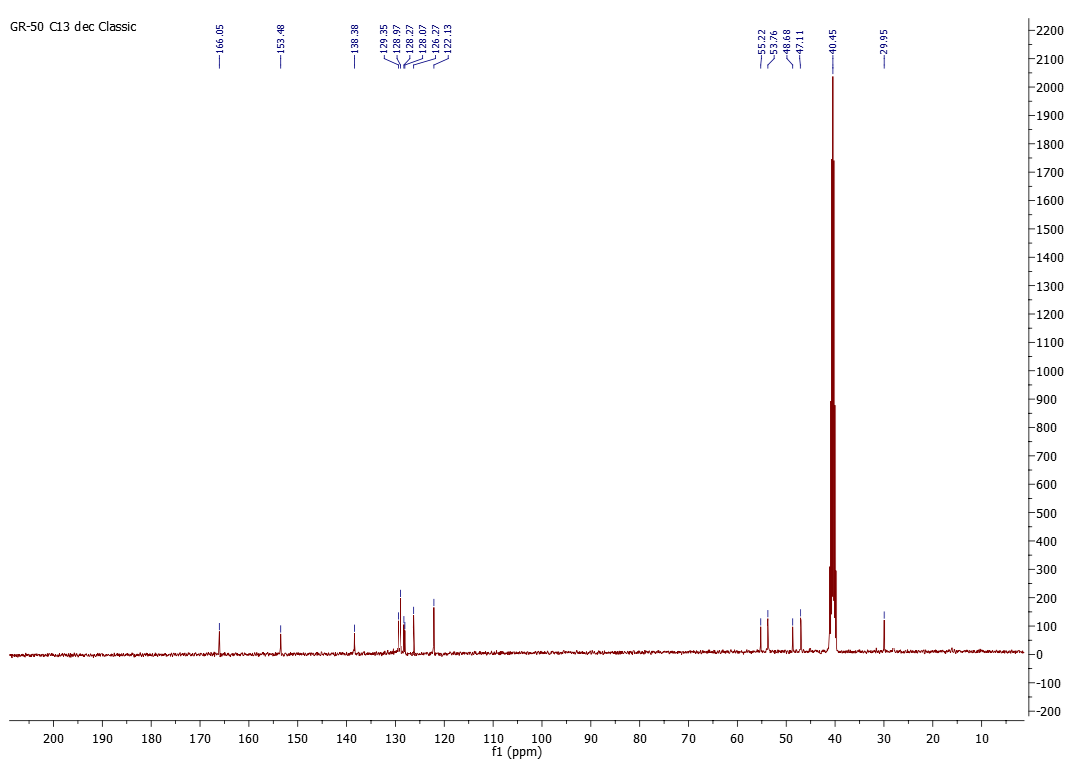


^13^C NMR spectrum of compound **45b** (100 MHz, DMSO-*d_6_*)

^1^H NMR spectrum of compound **44d** (400 MHz, DMSO-*d_6_*)


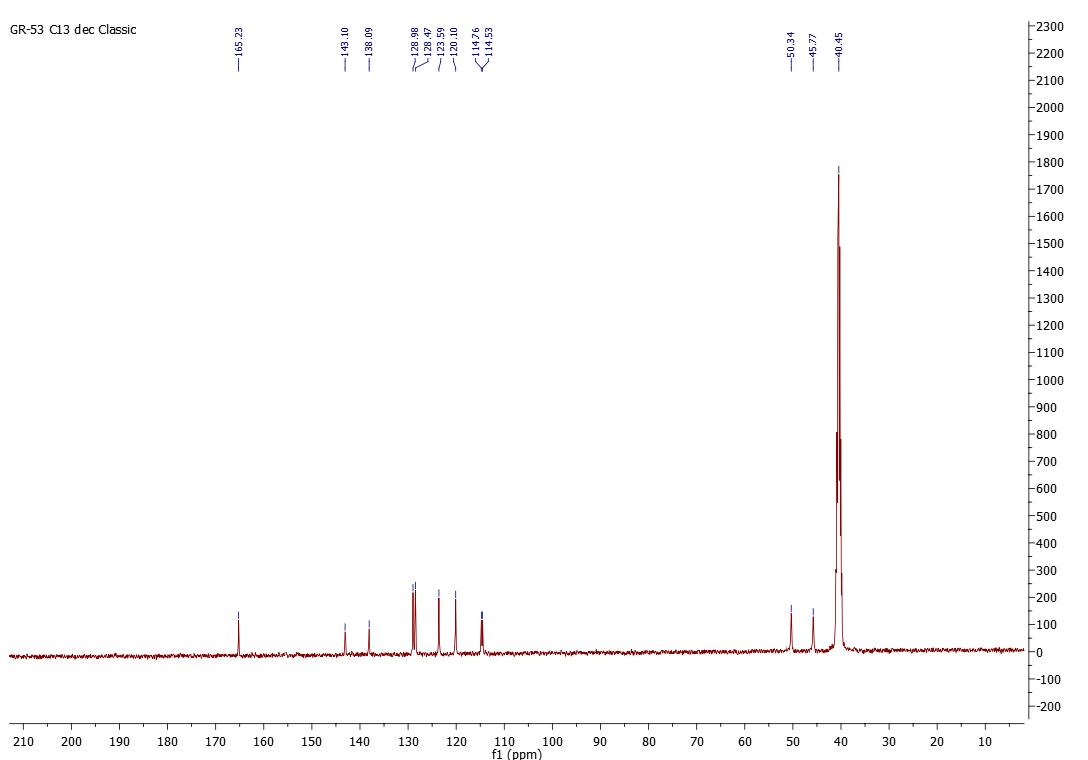


^13^C NMR spectrum of compound **44d** (100 MHz, DMSO-*d_6_*)

^1^H NMR spectrum of compound **44b** (400 MHz, DMSO-*d_6_*)


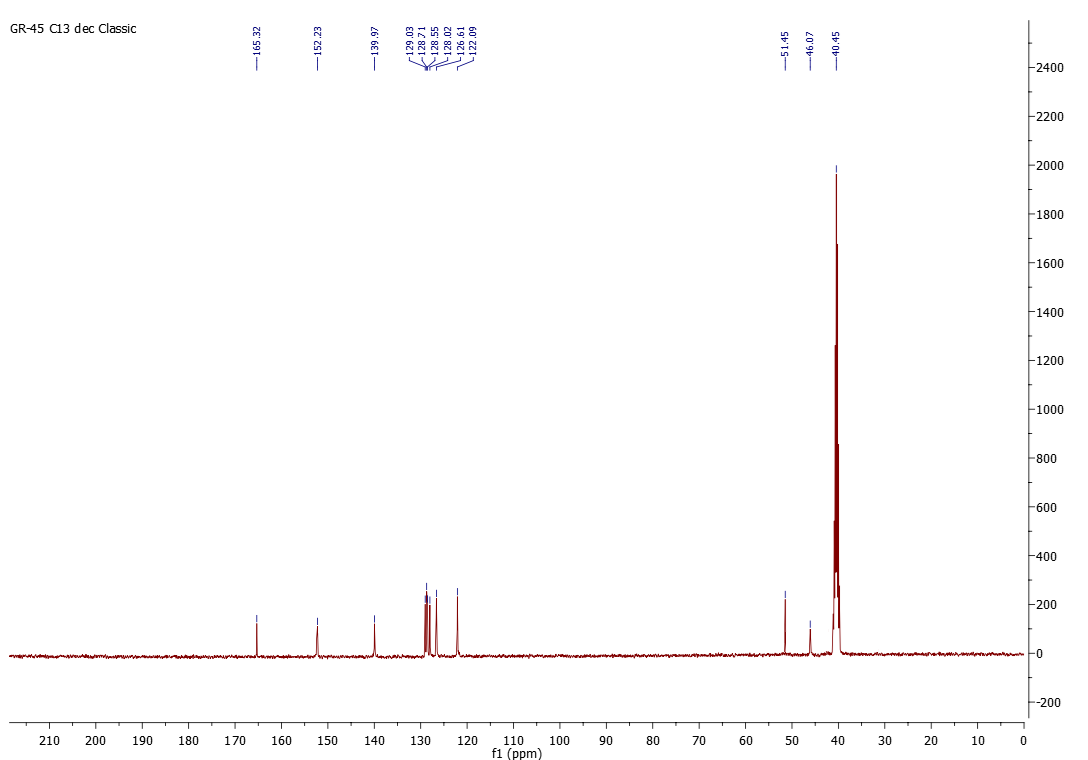


^13^C NMR spectrum of compound **44b** (100 MHz, DMSO-*d_6_*)

^^

^1^H NMR spectrum of compound **44c** (400 MHz, DMSO-*d_6_*)


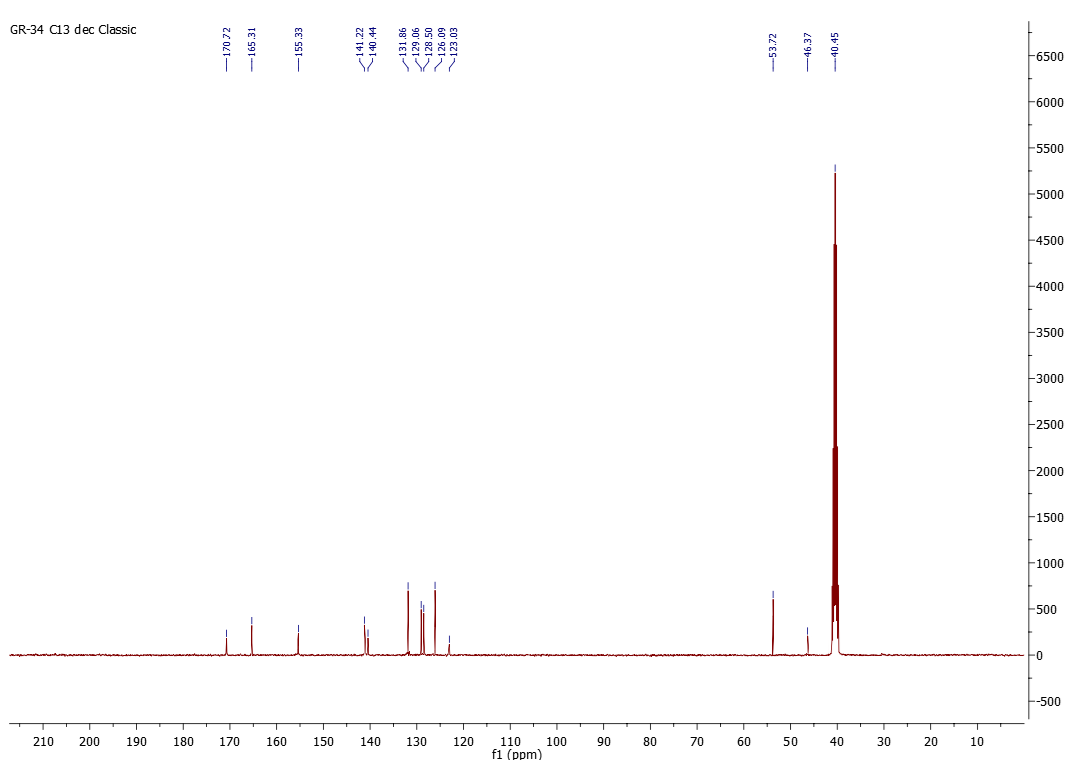


^13^C NMR spectrum of compound **44c** (100 MHz, DMSO-*d_6_*)

^1^H NMR spectrum of compound **30a** (400 MHz, DMSO-*d_6_*)

^1^H NMR spectrum of compound **30b** (400 MHz, DMSO-*d_6_*)

^1^H NMR spectrum of compound **30c** (400 MHz, DMSO-*d_6_*)

^13^C NMR spectrum of compound **30c** (100 MHz, DMSO-*d_6_*)

^1^H NMR spectrum of compound **31a** (400 MHz, DMSO-*d_6_*)

^13^C NMR spectrum of compound **31a** (100 MHz, DMSO-*d_6_*)

^1^H NMR spectrum of compound **31b** (400 MHz, DMSO-*d_6_*)


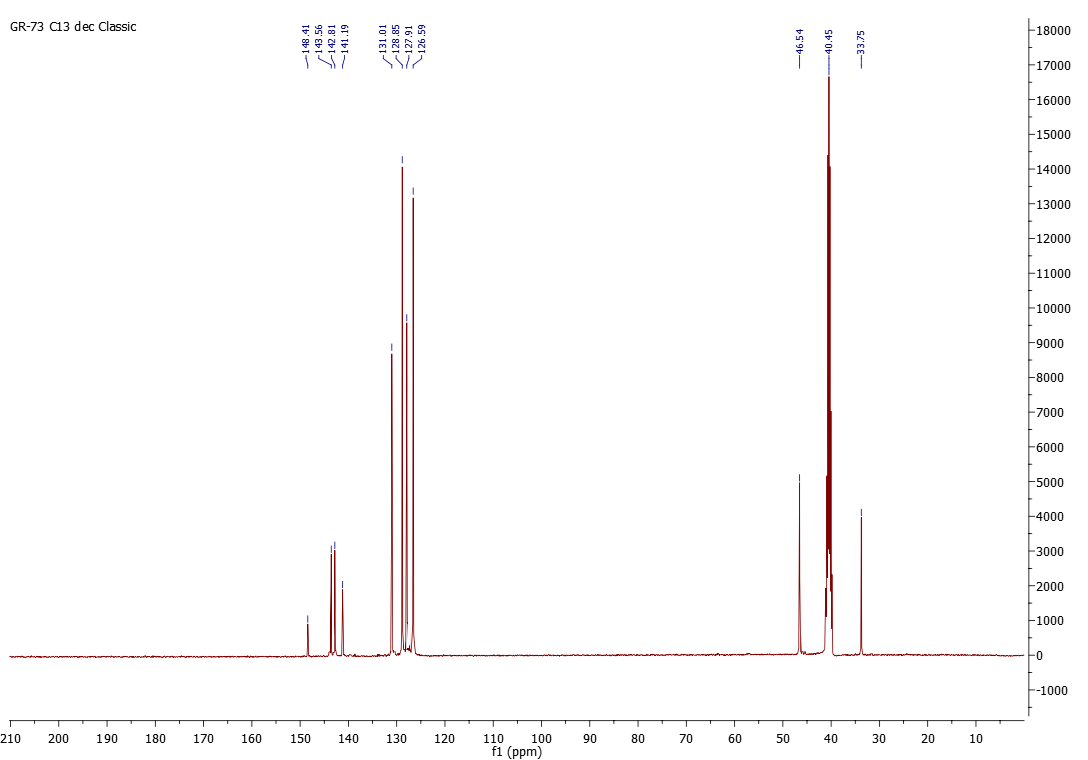


^13^C NMR spectrum of compound **31b** (100 MHz, DMSO-*d_6_*)

^1^H NMR spectrum of compound **31c** (400 MHz, DMSO-*d_6_*)

^13^C NMR spectrum of compound **31c** (100 MHz, DMSO-*d_6_*)

^1^H NMR spectrum of compound **21** (400 MHz, DMSO-*d_6_*)

^13^C NMR spectrum of compound **21** (100 MHz, DMSO-*d_6_*)


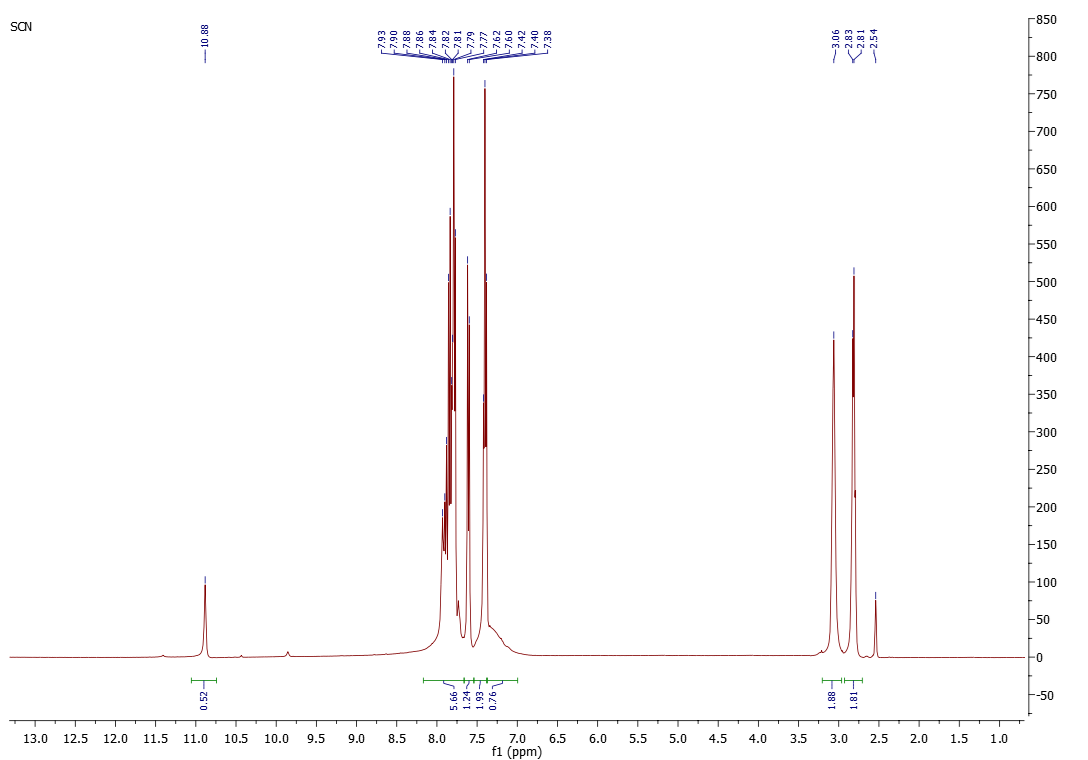


^1^H NMR spectrum of compound **22** (400 MHz, DMSO-*d_6_*)


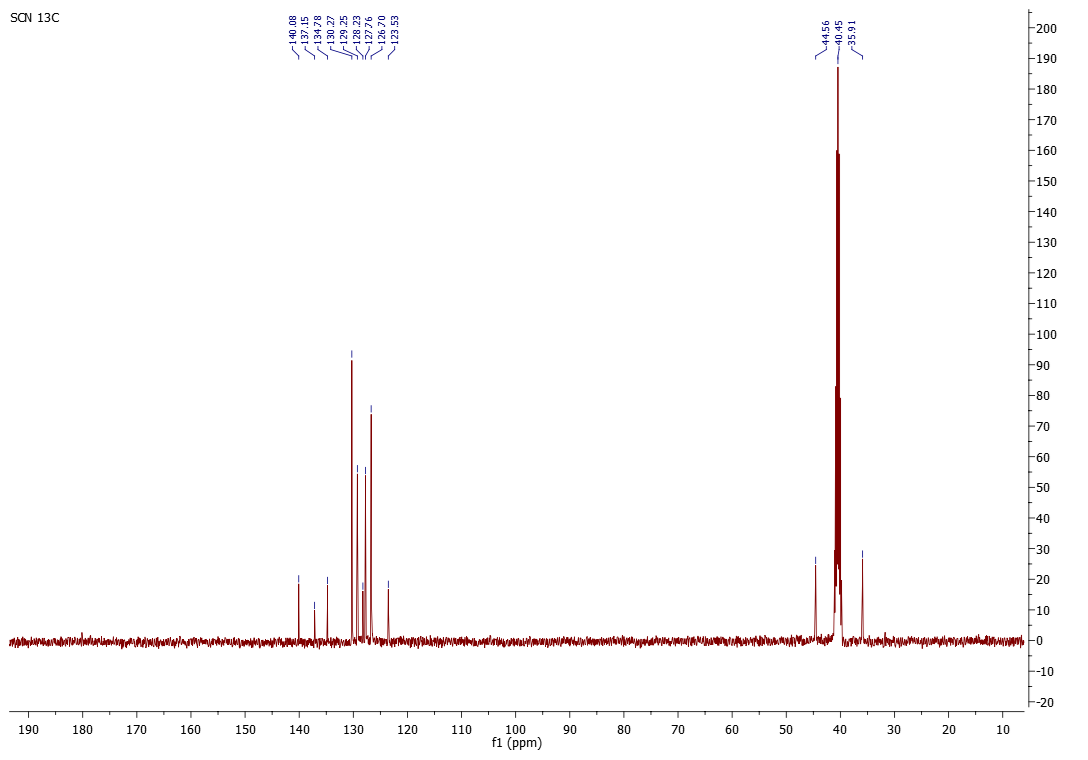


^13^C NMR spectrum of compound **22** (100 MHz, DMSO-*d_6_*)


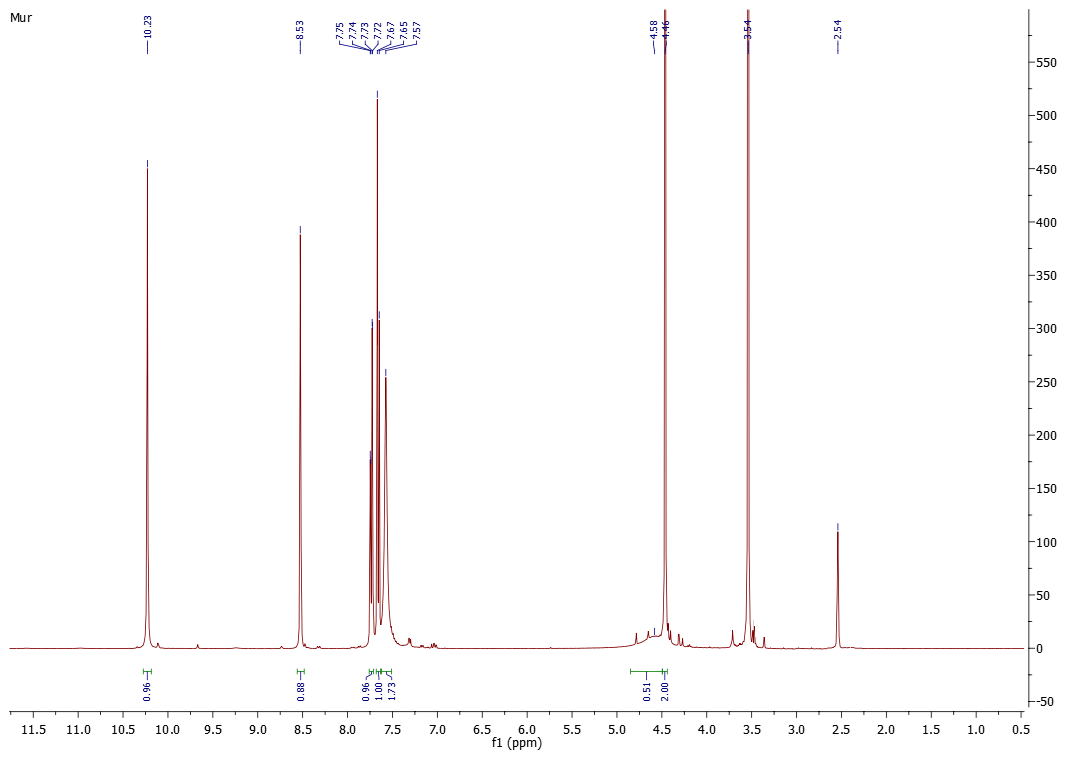


^1^H NMR spectrum of compound **34** (400 MHz, DMSO-*d_6_*)


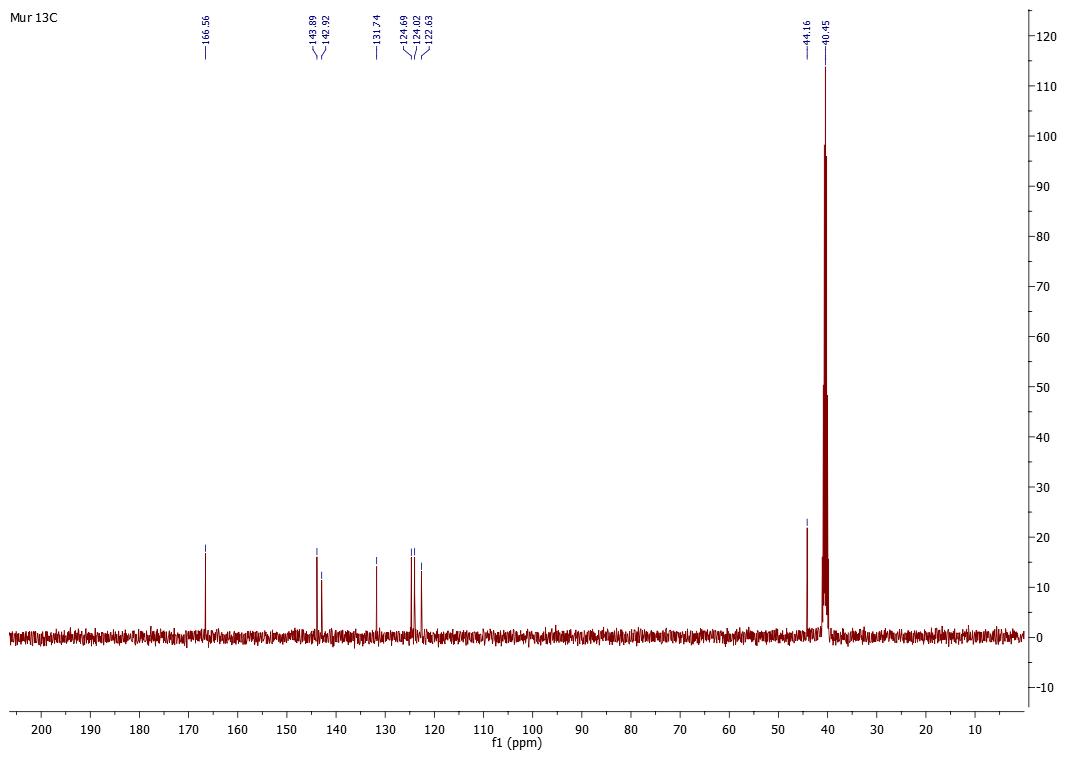


^13^C NMR spectrum of compound **34** (100 MHz, DMSO-*d_6_*)

^1^H NMR spectrum of compound **6a** (400 MHz, DMSO-*d_6_*)

^13^C NMR spectrum of compound **6a** (100 MHz, DMSO-*d_6_*)

**General** **procedure** **for** **the** **synthesis** **of** **intermediates** **3-6. [1]**

In a round bottom flask, the appropriate sulfonamide (**15,** **17-18)** (1.0 equiv) was dissolved in acetone (4 mL) at 0 °C followed by the addition of K_2_CO_3_ (1.2 equiv). Phenyl chloroformate **14** (1.05 equiv) was then added dropwise and the reaction mixture was stirred for 3 hours at 0 °C. A control via TLC was performed to assure the complete consumption of the starting materials. The reaction mixture was evaporated under reduced pressure to remove the solvent and H_2_O was added to quench the reaction to afford a solid, which was filtered off under vacuum, washed with Et_2_O and H_2_O, then dried on air. The compounds were pure enough to be used as they are.

**Phenyl** **(*R*)-(5-sulfamoyl-2,3-dihydro-1*H*-inden-1-yl)carbamate** **(3)**

White solid 45% yield; ^1^H NMR (DMSO-*d_6_*, 400 MHz): 8.37 (1H, d, *J* = 8.48 Hz), 7.78 (1H, s), 7.73 (1H, d, *J* = 7.85 Hz), 7.45 (3H, m), 7.40 (2H, bs, SO_2_N*H*_2_, exchange with D_2_O), 7.27 (1H, t, *J* = 7.36 Hz), 7.21 (2H, d, *J* = 7.89 Hz), 5.18 (1H, q, *J* = 8.16 Hz), 3.04 (1H, bs, N*H*, exchange with D_2_O), 2.90 (1H, m), 2.50 (1H, s), 2.00 (1H, m); ^13^C NMR (DMSO-*d_6_*, 100 MHz): 155.4, 152.0, 148.0, 145.7, 143.8, 130.3, 126.2, 126.0, 123.0, 122.8, 122.2, 56.5, 33.7, 30.6; ESI-HRMS (*m*/*z*) calculated for [M+H]^+^ ion species C_16_H_16_N_2_O_4_S 333.3740, found 333.3746.

**Phenyl** **(3-sulfamoylphenyl)carbamate** **(5a)**

White solid 55% yield; ^1^H NMR (DMSO-*d_6_*, 400 MHz): 10.59 (1H, bs, N*H*, exchange with D_2_O), 8.12 (1H, s), 7.69 (1H, s), 7.55 (2H, bs, SO_2_N*H*_2_, exchange with D_2_O), 7.48 (2H, m), 7.42 (2H, m), 7.29 (3H, m); ^13^C NMR (DMSO-*d_6_*, 100 MHz): 153.4, 151.7, 141.5, 136.1, 131.5, 129.9, 126.6, 125.8, 123.3, 120.9, 118.7; ESI-HRMS (*m*/*z*) calculated for [M+H]^+^ ion species C_13_H_12_N_2_O_4_S 293.3040, found 293.3046.

**Phenyl (4-sulfamoylphenyl)carbamate (5b)**

White solid 95% yield; ^1^H NMR (DMSO-*d_6_*, 400 MHz): 10.66 (1H, s, N*H*, exchange with D_2_O), 7.80 (2H, d, *J* = 8.3 Hz), 7.68 (2H, d, *J* = 8.3 Hz), 7.47 (2H, t, *J* = 8 Hz), 7.29 (5H, m, SO_2_N*H*_2_, exchange with D_2_O+ Ar-*H*); ^13^C NMR (DMSO-*d_6_*, 100 MHz): 151.3, 150.0, 141.2, 136.5, 129.4, 129.1, 125.5, 121.6, 118.0; ESI-HRMS (*m*/*z*) calculated for [M+H]^+^ ion species C_13_H_12_N_2_O_4_S 293.3090, found 293.3084.

**Phenyl (4-sulfamoylbenzyl)carbamate (5c)**

White solid 87% yield; ^1^H NMR (DMSO-*d_6_*, 400 MHz); 8.42 (1H, t, *J* = 5.5 Hz, N*H*, exchange with D_2_O), 7.83 (2H, d, *J* = 7.1 Hz), 7.52 (2H, d, *J* = 7.1 Hz), 7.40 (4H, m), 7.36 (2H, d, exchange with D_2_O, SO_2_N*H*_2_), 7.23 (1H, t, *J* = 7.4 Hz), 7.15 (2H, d, *J* = 7.4 Hz), 4.37 (2H, d, *J* = 5.5 Hz); ^13^C NMR (DMSO-*d_6_*, 100 MHz): 155.0, 151.3, 141.7, 141.1, 129.1, 128.2, 127.2, 125.5, 121.6, 45.1; ESI-HRMS (*m*/*z*) calculated for [M+H]^+^ ion species C_16_H_15_N_5_O_4_S 307.3360, found 307.3364.

**Phenyl (4-sulfamoylphenethyl)carbamate (5d)**

White solid 94% yield; ^1^H NMR (DMSO-*d_6_*, 400 MHz): 7.88 (1H, t, *J* = 5.6 Hz, N*H*, exchange with D_2_O), 7.79 (2H, d, *J* = 7.7 Hz), 7.46 (2H, d, *J* = 7.7 Hz), 7.38 (2H, d, *J* = 7.7 Hz), 7.33 (2H, s, SO_2_N*H*_2_, exchange with D_2_O), 7.21 (1H, t, *J* = 7.7 Hz), 7.08 (2H, d, *J* = 7.7 Hz); ^13^C NMR (DMSO-*d_6_*, 100 MHz): 153.4, 151.3, 142.6, 140.9, 129.1, 128.3, 128.0, 125.5, 121.6, 39.9, 35.0; ESI-HRMS (*m*/*z*) calculated for [M+H]^+^ ion species C_15_H_16_N_2_O_4_S 321.3630, found 321.3626.

**Phenyl *N*-[(1-(4-sulfamoylphenyl)-1*H*-1,2,3-triazol-4-yl)methyl]carbamate (6a)**

White solid 54% yield; ^1^H NMR (DMSO-*d_6_*, 400 MHz): 8.88 (1H, s), 8.46 (2H, s), 8.19 (1H, d, *J* = 6.76 Hz), 7.96 (1H, d, *J* = 6.79 Hz), 7.84 (1H, t, *J* = 6.88 Hz, N*H*, exchange with D_2_O), 7.40 (2H, m), 7.23 (1H, m), 7.16 (2H, d, *J* = 7.11 Hz), 4.46(2H, m); ^13^C NMR (DMSO-*d_6_*, 100 MHz): 155.5, 152.0, 147.3, 146.7, 137.7, 131.9, 130.3, 126.4, 126.1, 124.0, 122.8, 122.4, 118.1, 37.1; ESI-HRMS (*m*/*z*) calculated for [M+H]^+^ ion species C_16_H_15_N_5_O_4_S 373.3870, found 373.3876.

**Phenyl *N*-[(1-(3-sulfamoylphenyl)-1*H*-1,2,3-triazol-4-yl)methyl]carbamate**

**(6b) [2]**

White solid 67% yield; ^1^H NMR (DMSO-*d_6_*, 400 MHz): 8.87 (1H, bs, N*H*, exchange with D_2_O), 8.45−8.43 (2H, m), 8.19 (1H, d, *J* = 7.5 Hz), 7.96 (1H, d, *J* = 7.5 Hz), 7.84 (1H, t, *J* = 7.9 Hz), 7.65 (2H, bs, SO_2_N*H*_2_, exchange with D_2_O), 7.41 (2H, t, *J* = 7.6 Hz), 7.24 (1H, t, *J* = 7.2 Hz), 7.17 (2H, d, *J* = 7.7 Hz), 4.47 (2H, d, *J* = 5.5 Hz); ^13^C NMR (DMSO-*d_6_*, 100 MHz): 55.6, 152.0, 147.4, 146.7, 137.7, 132.0, 130.3,126.5, 126.1, 124.0, 122.8, 122.5, 118.2, 37.1; ESI-HRMS (*m*/*z*) calculated for [M+H]^+^ ion species C_16_H_15_N_5_O_4_S 373.3870, found 373.3866.

**General** **procedure** **for** **the** **synthesis** **of** **isothiocyanate** **derivatives** **20a-d,** **21 and 22**

In a dry flask and under inert atmosphere (nitrogen flow), the appropriate amine **(17c-d,** **29,** **11)** (1.0 equiv) was dissolved in dry DMF (4 mL) at 0 °C followed by the addition of CS_2_ (4 mL), pyridine (3.0 equiv) and EDC HCl (1.2 equiv). The cooling bath was removed and the reaction mixture was stirred at room temperature overnight. A control via TLC was performed to assure the complete consumption of the starting materials. HCl 1M was added to quench the reaction affording a solid, which was filtered off under vacuum, washed with Et_2_O and H_2_O, then dried on air. The compounds were pure enough to be used as they are.

**4-(Isothiocyanatomethyl)benzenesulfonamide** **(20a)**

White solid 34% yield; ^1^H NMR (DMSO-*d_6_*, 400 MHz): 7.90 (2H, d, *J* = 6.73 Hz), 7.60 (2H, d, *J* = 6.23 Hz), 7.45 (2H, bs, SO_2_N*H*_2_, exchange with D_2_O), 5.09 (2H, s); ESI-HRMS (*m*/*z*) calculated for [M+H]^+^ ion species C_8_H_8_N_2_O_2_S_2_ 228.28400, found 228.2846.

**4-(2-Isothiocyanatoethyl)benzenesulfonamide** **(20b)**

Light yellow solid 64% yield; ^1^H NMR (DMSO-*d_6_*, 400 MHz): 7.82 (d, 2H, *J* = 7.89 Hz); 7.52 (d, 2H, *J* = 7.84 Hz); 7.38 (2H, bs, SO_2_N*H*_2_, exchange with D_2_O), 4.00 (2H, t, *J* = 5.94 Hz), 3.08 (2H, t, *J* = 5.95 Hz); ESI-HRMS (*m*/*z*) calculated for [M+H]^+^ ion species C_9_H_10_N_2_O_2_S_2_ 242.3110, found 242.3118.

**3-Isothiocyanatobenzenesulfonamide (20c) [3]**

Light yellow solid 81% yield; ^1^H NMR (DMSO-*d_6_*, 400 MHz): 7.92 (1H, s), 7.89 (1H, d, *J* = 8.4 Hz), 7.68 (1H, dd, *J* = 8.4 Hz), 7.63 (1H, d, *J* = 8.4 Hz), 7.48 (2H, s, exchange with D_2_O, SO_2_N*H*_2_); ESI-HRMS (*m*/*z*) calculated for [M+H]^+^ ion species C_7_H_6_N_2_O_2_S_2_ 214.2570, found 214.2582.

**4-Isothiocyanatobenzenesulfonamide (20d) [4]**

Light yellow solid 79% yield; ^1^H NMR (DMSO-*d_6_*, 400 MHz): 7.87 (2H, d, *J* = 7.5 Hz), 7.58 (2H, d, *J* = 7.5 Hz), 7.49 (2H, s, exchange with D_2_O, SO_2_N*H*_2_); ESI-HRMS (*m*/*z*) calculated for [M+H]^+^ ion species C_7_H_6_N_2_O_2_S_2_ 214.2570, found 214.2566.

**4-Isothiocyanato-*N*-(4-sulfamoylbenzyl)benzamide** **(21)**

Light brown solid 38% yield; ^1^H NMR (DMSO-*d_6_*, 400 MHz): 9.28 (1H, t, N*H*, exchange with D_2_O, *J* = 5.09 Hz), 8.00 (2H, d, *J* = 8.14 Hz), 7.82 (2H, d, *J* = 7.86 Hz), 7.58 (2H, d, *J* = 8.17 Hz), 7.52 (2H, d, *J* = 7.87 Hz), 7.35 (2H, bs, SO_2_N*H*_2_, exchange with D_2_O), 4.57 (2H, d, *J* = 5.33 Hz); ^13^C NMR (DMSO-*d_6_*, 100 MHz): 166.0, 144.5, 143.6, 135.9, 134.0, 133.6, 129.9, 128.5, 127.0, 126.7, 43.4; ESI-HRMS (*m*/*z*) calculated for [M+H]^+^ ion species C_15_H_13_N_3_O_3_S_2_ 347.4070, found 347.4078.

**4-Isothiocyanato-*N*-(4-sulfamoylphenethyl)benzenesulfonamide (22)**

Light brown solid 69% yield; ^1^H NMR (DMSO-*d_6_*, 400 MHz): 10.88 (1H, s, N*H*, exchange with D_2_O), 7.93-7.77 (6H, m, SO_2_N*H*_2_, exchange with D_2_O+ Ar-*H*), 7.61 (1H, d, *J* = 8.29 Hz), 7.41 (2H, d, *J* = 7.16 Hz), 7.38 (1H, bs), 3.06 (2H, m), 2.82 (2H, d, *J* = 6.18 Hz); ^13^C NMR (DMSO-*d_6_*, 100 MHz): 140.1, 137.1, 134.8, 130.3, 129.2, 128.2, 127.8, 126.7, 123.5, 44.6, 35.9; ESI-HRMS (*m*/*z*) calculated for [M+H]^+^ ion species C_15_H_15_N_3_O_4_S_3_ 397.4820, found 397.4832.

**Synthesis of 2-oxo-2,3-dihydrobenzo[*d*]oxazole-5-sulfonamide** **(12)**

In a dry flask, 3-amino-4-hydroxybenzenesulfonamide **11** (1.5 g; 7.97 mmol; 1.0 equiv) was dissolved in dry THF (16.0 mL) followed by the addition of triethylamine (2.2 mL; 15.94 mmol; 2.0 equiv). A solution of Triphosgene (2.13 g; 7.17 mmol; 0.9 equiv) dissolved in dry THF (10.2 mL) was then added dropwise at 0 °C over 20 min. The cooling bath was removed and the reaction mixture was stirred for 2 hours at room temperature. A control via TLC was performed to assure the complete consumption of the starting materials. The reaction was quenched with NaHCO_3_ saturated solution and the product was extracted with EtOAc. The organic layers were dried over Na_2_SO_4_ and evaporated under reduced pressure. Compound **12** was pure enough to be used as it is.

Grey solid 95% yield; ^1^H NMR (DMSO-*d_6_*, 400 MHz): 14.19 (1H, bs, N*H*, exchange with D_2_O), 7.77 (1H, dd, *J* = 8.56 Hz; *J* = 1.61 Hz), 7.70 (1H, d, *J* = 8.56 Hz), 7.65 (1H, bs); 7.49 (2H, bs, SO_2_N*H*_2_, exchange with D_2_O); ^13^C NMR (DMSO-*d_6_*, 100 MHz): 153.4, 141.5, 136.1, 129.9, 126.6, 125.8, 120.9; ESI-HRMS (*m*/*z*) calculated for [M+H]^+^ ion species C_7_H_6_N_2_O_4_S 214.1950, found 214.1956.

**Synthesis of 2-thioxo-2,3-dihydrobenzo[*d*]oxazole-5-sulfonamide** **(27)**

3-Amino-4-hydroxybenzenesulfonamide **11** (1.0 equiv) was dissolved in dry DMF (4 mL) at 0 °C followed by the addition of CS_2_ (4 mL), pyridine (3.0 equiv) and EDC HCl (1.2 equiv). The reaction mixture was stirred at room temperature overnight. HCl 1M was added to quench the reaction affording a solid, which was filtered off under vacuum, washed with Et_2_O and H_2_O, and dried on air to yield compounds **27**. Brown solid 64% yield; ^1^H NMR (DMSO-*d_6_*, 400 MHz): 14.19 (1H, bs, N*H*, exchange with D_2_O), 7.76 (1H, d, *J* = 8.39 Hz), 7.70 (1H, d, *J* = 8.44 Hz), 7.65 (1H, s), 7.49 (2H, bs, SO_2_N*H*_2_, exchange with D_2_O); ^13^C NMR (DMSO-*d_6_*, 100 MHz): 182.0, 150.8, 142.2, 132.6, 122.7, 111.2, 108.9; ESI-HRMS (*m*/*z*) calculated for [M+H]^+^ ion species C_7_H_6_N_2_O_3_S_2_ 230.2560, found 230.2568.

**General** **procedure** **for** **the** **synthesis** **of** **compounds** **30a-c**

In a round bottom flask, the appropriate sulfonamide (**17b-d**) (1.0 equiv) was dissolved in Acetone (4 mL) at 0 °C followed by the addition of K_2_CO_3_ (1.2 equiv) and 4-(chloromethyl)benzoyl chloride (**40**) (1.0 equiv). The reaction mixture was stirred for 1 hour at 0 °C. A control via TLC was performed to assure the complete consumption of the starting materials. The reaction mixture was evaporated under reduced pressure to remove the solvent and H_2_O was then added to quench the reaction to afford a solid, which was filtered-off under *vacuo*, washed with Et_2_O and H_2_O, then dried on air. The compounds were pure enough to be used as they are.

**4-(Chloromethyl)-*N*-(4-sulfamoylphenyl)benzamide** **(30a)**

White solid 88% yield; ^1^H NMR (DMSO-*d_6_*, 400 MHz): 10.63 (1H, bs, N*H*, exchange with D_2_O), 8.00 (4H, ap t, *J* = 9.26 Hz), 7.85 (2H, d, *J* = 8.77 Hz), 7.65 (2H, d, *J* = 8.23 Hz), 7.34 (2H, bs, SO_2_N*H*_2_, exchange with D_2_O), 4.89 (2H, s); ^13^C NMR (DMSO-*d_6_*, 100 MHz): 166.4, 143.0, 142.4, 139.7, 135.3, 129.8, 129.2, 127.5, 120.8, 36.3; ESI-HRMS (*m*/*z*) calculated for [M+H]^+^ ion species C_14_H_13_ClN_2_O_3_S 324.7790, found 324.7784.

**4-(Chloromethyl)-*N*-(4-sulfamoylbenzyl)benzamide** **(30b)**

White solid 64% yield; ^1^H NMR (DMSO-*d_6_*, 400 MHz): 9.22 (1H, bs, N*H*, exchange with D_2_O), 7.94 (2H, d, *J* = 7.88 Hz), 7.82 (2H, d, *J* = 8.04 Hz), 7.58 (2H, d, *J* = 7.92 Hz), 7.53 (2H, d, *J* = 7.96 Hz), 7.36 (2H, bs, SO_2_N*H*_2_, exchange with D_2_O); 4.86 (2H, s), 4.58 (2H, m); ^13^C NMR (DMSO-*d_6_*, 100 MHz): 166.8, 144.6, 143.5, 141.8, 134.9, 129.7, 128.6, 128.5, 126.7, 46.4, 43.3; ESI-HRMS (*m*/*z*) calculated for [M+H]^+^ ion species C_15_H_15_ClN_2_O_3_S 338.8060, found 338.8064.

**4-(Chloromethyl)-*N*-(4-sulfamoylphenethyl)benzamide** **(30c)**

White solid 34% yield; ^1^H NMR (DMSO-*d_6_*, 400 MHz): 8.67 (1H, t, N*H*, exchange with D_2_O, *J* = 5.42 Hz), 7.85 (2H, d, *J* = 8.16 Hz), 7.78 (2H, d, *J* = 8.16 Hz), 7.55 (2H, d, *J* = 8.12 Hz), 7.47 (2H, d, *J* = 8.16 Hz), 7.34 (2H, bs, SO_2_N*H*_2_, exchange with D_2_O); 4.84 (2H, s), 3.55 (2H, q, *J* = 6.54 Hz), 2.96 (2H, t, *J* = 7.12 Hz); ^13^C NMR (DMSO-*d_6_*, 100 MHz): 166.7, 144.7, 143.0, 141.5, 135.3, 130.1, 129.6, 128.4, 126.6, 46.4, 41.4, 35.7; ESI-HRMS (*m*/*z*) calculated for [M+H]^+^ ion species C_16_H_17_ClN_2_O_3_S 352.8330, found 352.8338.

**General** **procedure** **for** **the** **synthesis** **of** **compounds** **31a-c**

In a round bottom flask, the appropriate sulfonamide (**42,** **17c-d)** (1.0 equiv) was dissolved in Acetone (4 mL) at 0 °C followed by the addition of K_2_CO_3_ (1.2 equiv) and 4-(bromomethyl)benzenesulfonyl chloride (**41**) (1.0 equiv). The reaction mixture was stirred for 1 hour at 0 °C. A control via TLC was performed to assure the complete consumption of the starting materials. The reaction mixture was evaporated under reduced pressure to remove the solvent and H_2_O was then added to quench the reaction to afford a solid, which was filtered off under vacuum, washed with Et_2_O and H_2_O, then dried on air. The compounds were pure enough to be used as they are.

**4-Sulfamoylphenyl** **4-(bromomethyl)benzenesulfonate** **(31a)**

Light yellow solid 89% yield; ^1^H NMR (DMSO-*d_6_*, 400 MHz): 7.96 (2H, d, *J* = 8.48 Hz), 7.88 (2H, d, *J* = 8.80 Hz), 7.79 (2H, d, *J* = 8.40 Hz), 7.50 (2H, bs, SO_2_N*H*_2_, exchange with D_2_O), 7.32 (2H, d, *J* = 8.80 Hz), 4.85 (2H, s); ^13^C NMR (DMSO-*d_6_*, 100 MHz): 148.4, 144.2, 143.2, 139.4, 130.5, 130.2, 127.5, 126.8, 63.3; ESI-HRMS (*m*/*z*) calculated for [M+H]^+^ ion species C_13_H_12_BrNO_5_S_2_ 406.2650, found 406.2658.

**4-(Bromomethyl)-*N*-(4-sulfamoylbenzyl)benzenesulfonamide** **(31b)**

White solid 78% yield; ^1^H NMR (DMSO-*d_6_*, 400 MHz): 8.35 (1H, t, N*H*, exchange with D_2_O, *J* = 6.30 Hz), 7.83 (2H, d, *J* = 8.30 Hz), 7.77 (2H, d, *J* = 8.27 Hz), 7.68 (2H, d, *J* = 8.30 Hz), 7.45 (2H, d, *J* = 8.26 Hz), 7.36 (2H, bs, SO_2_N*H*_2_, exchange with D_2_O), 4.80 (2H, s), 4.10 (2H, d, *J* = 6.27 Hz); ^13^C NMR (DMSO-*d_6_*, 100 MHz): 148.4, 144.2, 143.2, 139.4, 130.5, 130.2, 127.5, 126.8, 63.3, 44.7; ESI-HRMS (*m*/*z*) calculated for [M+H]^+^ ion species C_14_H_15_BrN_2_O_4_S_2_ 419.3080, found 419.3096.

**4-(Bromomethyl)-*N*-(4-sulfamoylphenethyl)benzenesulfonamide** **(31c)**

White solid 96% yield; ^1^H NMR (DMSO-*d_6_*, 400 MHz): 7.89 (1H, bs, N*H*, exchange with D_2_O), 7.79 (2H, d, *J* = 8.28 Hz), 7.76 (2H, d, *J* = 8.16 Hz), 7.68 (2H, d, *J* = 8.28 Hz), 7.38 (2H, d, *J* = 8.12 Hz), 7.32 (2H, bs, SO_2_N*H*_2_, exchange with D_2_O), 4.80 (2H, s), 3.06 (2H, q, *J* = 6.65 Hz), 2.80 (2H, t, *J* = 7.10 Hz); ^13^C NMR (DMSO-*d_6_*, 100 MHz): 148.4, 144.2, 143.2, 139.4, 130.5, 130.2, 127.5, 126.8, 63.3, 44.7, 35.9; ESI-HRMS (*m*/*z*) calculated for [M+H]^+^ ion species C_15_H_17_BrN_2_O_4_S_2_ 433.3350, found 433.3362.

**General procedure for the synthesis of compounds 32a-c-34**

To a solution of appropriate aniline (1 equiv) and K_2_CO_3_ (2 equiv) in acetone (20 mL), 2-chloroacetyl chloride (1.1 equiv) was added drop-wise. The reaction mixture was stirred at 0 °C for 0.5 h then left to warm to room temperature. The progress of the reaction was monitored by TLC till completion. The reaction mixture was filtered and the filtrate was evaporated under reduced pressure to afford the crude product, which was washed with water and crystallized from ethanol to afford analytically pure product of compound

**2-Chloro-*N*-(4-sulfamoylphenyl)acetamide (32a)**

White solid 95% yield; ^1^H NMR (DMSO-*d_6_*, 400 MHz): 10.66 (1H, s, N*H*, exchange with D_2_O) 8.07–8.09 (2H, d, *J* = 8.5 Hz), 8.01–8.03 (2H, d, *J* = 9.0 Hz), 7.31 (2H, s, SO_2_N*H*_2_, exchange with D_2_O), 4.33 (2H, s). ^13^C NMR (DMSO-*d_6_*, 100 MHz): 166.3, 142.4, 140.1, 127.8, 120.1, 44.6,; ESI-HRMS (*m*/*z*) calculated for [M+H]^+^ ion species C_8_H_9_ClN_2_O_3_S 248.6810, found 248.6814.

**3-Chloro-*N*-(4-sulfamoylphenyl)propenamide (32b) [5]**

White solid 70% yield; ^1^H NMR (DMSO-*d_6_*, 400 MHz): 10.3 (1H, bs, N*H*, exchange with D_2_O), 7.8 (4H, m), 7.00 (2H, bs, SO_2_N*H*_2_, exchange with D_2_O), 3.87 (2H, t, *J* = 6.00 Hz), 2.88 (2H, t, *J* = 6.00 Hz); ^13^C NMR (DMSO-*d_6_*, 100 MHz): 172.8, 140.8, 136.9, 125.7, 118.0, 38.7, 37.9; ESI-HRMS (*m*/*z*) calculated for [M+H]^+^ ion species C_9_H_11_ClN_2_O_3_S 262.7080, found 262.7072.

**3-Chloro-*N*-(3-sulfamoylphenyl)propanamide (32c) [6]**

White solid 58% yield; ^1^H NMR (DMSO-*d_6_*, 400 MHz): 10.39 (1H, bs, N*H*, exchange with D_2_O), 8.27 (1H, m), 7.74 (1H, m), 7.58 (2H, d, *J* = 8.28 Hz), 7.38 (2H, bs, SO_2_N*H*_2_, exchange with D_2_O), 3.90 (2H, t, *J* = 6.2 Hz), 2.86 (2H, t, *J* = 6.2 Hz); ESI-HRMS (*m*/*z*) calculated for [M+H]^+^ ion species C_9_H_11_ClN_2_O_3_S 262.7080, found 262.7094.

**Synthesis of 2-chloro-*N*-(2-hydroxy-5-sulfamoylphenyl)acetamide (34)**

Yellow solid 81% yield; ^1^H NMR (DMSO-*d_6_*, 400 MHz): 10.23 (1H, s, N*H*, exchange with D_2_O), 8.53 (1H, s), 7.73 (1H, dd, *J* = 8.60Hz; *J* = 2.16 Hz), 7.66 (1H, d, *J* = 8.60 Hz), 7.57 (2H, bs, SO_2_N*H*_2_, exchange with D_2_O), 4.58 (1H, bs, O*H*, exchange with D_2_O), 4.46 (2H, s); ^13^C NMR (DMSO-*d_6_*, 100 MHz): 166.6, 143.9, 142.9, 131.7, 124.7, 124.0, 122.6, 44.2; ESI-HRMS (*m*/*z*) calculated for [M+H]^+^ ion species C_8_H_9_ClN_2_O_4_S 264.6800, found 264.6894.

**General** **procedure** **for** **the** **synthesis** **of** **compounds** **43a-b, 44a-d, 45a-d and 46**

In a round bottom flask, the appropriate sulfonamide (**17a-b,** **42,** **53-54**) (1.0 equiv) was dissolved in Acetone (4 mL) at 0 °C followed by the addition of K_2_CO_3_ (1.2 equiv). Acryloyl chloride (**51**) (1.05 equiv) was then added dropwise and the reaction mixture was stirred for 1 hour at 0 °C. A control via TLC was performed to assure the complete consumption of the starting materials. The reaction mixture was evaporated under reduced pressure to remove the solvent and the excess of Acryloyl chloride. H_2_O was then added to quench the reaction to afford a solid, which was filtered off under vacuum, washed with Et_2_O and H_2_O, then dried on air. The compounds were pure enough to be used as they are.

***N*-(3-Sulfamoylphenyl)acrylamide** **(43a)**

White solid 43% yield; ^1^H NMR (DMSO-*d_6_*, 400 MHz): 10.50 (1H, s), 8.27 (1H, s), 7.87 (1H, s), 7.56 (2H, d, *J* = 4.68 Hz), 7.42 (2H, bs, SO_2_N*H*_2_, exchange with D_2_O), 6.48 (1H, dd, *J* = 16.98 Hz; *J* = 10.04 Hz), 6.34 (1H, d, *J* = 16.85 Hz), 5.84 (1H, d, *J* = 10.04 Hz); ^13^C NMR (DMSO-*d_6_*, 100 MHz): 164.4, 145.6, 140.3, 132.5, 130.5, 128.5, 123.1, 121.5, 117.3; ESI-HRMS (*m*/*z*) calculated for [M+H]^+^ ion species C_9_H_10_N_2_O_3_S 226.2500, found 226.2514.

***N*-(4-Sulfamoylphenyl)acrylamide** **(43b)**

White solid 44% yield; ^1^H NMR (DMSO-*d_6_*, 400 MHz): 10.51 (1H, s), 7.86 (2H, d, *J* = 8.24 Hz), 7.81 (2H, d, *J* = 8.28 Hz), 7.29 (2H, bs, SO_2_N*H*_2_, exchange with D_2_O), 6.49 (1H, dd, *J* = 16.95 Hz; *J* = 10.31 Hz), 6.34 (1H, d, *J* = 16.77 Hz), 5.85 (1H, d, *J* = 9.92 Hz); ^13^C NMR (DMSO-*d_6_*, 100 MHz): 164.4, 145.6, 140.3, 132.5, 130.5, 128.5, 117.3; ESI-HRMS (*m*/*z*) calculated for [M+H]^+^ ion species C_9_H_10_N_2_O_3_S 226.2500, found 226.2518.

**4-(4-Acryloylpiperazin-1-yl)benzenesulfonamide** **(44a)**

White solid 72% yield; ^1^H NMR (DMSO-*d_6_*, 400 MHz): 7.67 (2H, d, *J* = 8.84 Hz), 7.11 (2H, bs, SO_2_N*H*_2_, exchange with D_2_O), 7.08 (2H, d, *J* = 8.96 Hz), 6.89 (1H, dd, *J* = 16.74 Hz; *J* = 10.42 Hz), 6.18 (1H, dd, *J* = 16.62 Hz; *J* = 2.18 Hz), 5.75 (1H, dd, *J* = 10.46 Hz; *J* = 2.24 Hz), 3.73 (4H, m), 3.34 (4H, m); ^13^C NMR (DMSO-*d_6_*, 100 MHz): 165.2, 153.4, 134.1, 129.0, 128.5, 128.0, 115.0, 48.4, 45.4; ESI-HRMS (*m*/*z*) calculated for [M+H]^+^ ion species C_13_H_17_N_3_O_3_S 295.3570, found 295.3562.

**4-(4-Acryloylpiperazin-1-yl)-3-chlorobenzenesulfonamide** **(44b)**

White solid 47% yield; ^1^H NMR (DMSO-*d_6_*, 400 MHz): 7.86 (1H, s), 7.76 (1H, d, *J* = 8.12), 7.41 (2H, bs, SO_2_N*H*_2_, exchange with D_2_O); 7.33 (1H, d, *J* = 7.42 Hz), 6.89 (1H, dd, *J* = 16.42 Hz; *J* = 11.26 Hz), 6.19 (1H, d, *J* = 16.19 Hz), 5.76 (1H, d, *J* = 9.68 Hz), 3.65 (4H, m), 3.16 (4H, m); ^13^C NMR (DMSO-*d_6_*, 100 MHz): 165.3, 152.3, 140.0, 129.0, 128.7, 128.6, 128.0, 126.7, 122.1, 52.0, 51.5; ESI-HRMS (*m*/*z*) calculated for [M+H]^+^ ion species C_13_H_16_ClN_3_O_3_S 329.7990, found 329.7884.

**4-(4-Acryloylpiperazin-1-yl)-3-(trifluoromethyl)benzenesulfonamide** **(44c)**

Yellow solid 80% yield; ^1^H NMR (DMSO-*d_6_*, 400 MHz): 8.12 (1H, s), 7.82 (1H, d, *J* = 8.44 Hz), 7.53 (2H, bs, SO_2_N*H*_2_, exchange with D_2_O), 7.51 (1H, d, *J* = 10.15 Hz), 6.87 (1H, dd, *J* = 16.63 Hz; *J* = 10.42 Hz), 6.18 (1H, d, *J* = 6.34 Hz), 5.75 (1H, d, *J* = 4.24 Hz), 3.65 (4H, m), 2.98 (4H, m); ^13^C NMR (DMSO-*d_6_*, 100 MHz): 165.3, 155.6 (q, *J*^1^_C-F_ = 273.93 Hz), 143.2, 133.8, 128.6, 123.7, 120.2, 114.8, 51.0, 45.8; ESI-HRMS (*m*/*z*) calculated for [M+H]^+^ ion species C_14_H_16_F_3_N_3_O_3_S 363.3552, found 363.3560.

**4-(4-Acryloylpiperazin-1-yl)-3-fluorobenzenesulfonamide** **(44d)**

Light yellow solid 56% yield; ^1^H NMR (DMSO-*d_6_*, 400 MHz): 7.58 (2H, t, *J* = 8.18 Hz), 7.36 (2H, bs, SO_2_N*H*_2_, exchange with D_2_O), 7.22 (1H, t, *J* = 8.52 Hz), 6.88 (1H, dd, *J* = 16.32 Hz; *J* = 10.42 Hz), 6.19 (1H, d, *J* = 16.64 Hz), 5.76 (1H, d, *J* = 10.32 Hz), 3.75 (4H, m), 3.16 (4H, m); ^13^C NMR (DMSO-*d_6_*, 100 MHz): 165.3, 155.6 (d, *J*^1^_C-F_ = 248.2 Hz), 143.2 (q, *J*^3^_C-F_ = 7.32 Hz), 138.2 (q, *J*^3^_C-F_ = 5.97 Hz), 129.0 (t, *J*^2^_C-F_ = 35.45 Hz), 128.6, 123.7, 120.2, 114.8, 51.0 (t, *J*^2^_C-F_ = 38.3 Hz), 45.8 (t, *J* = 18.8 Hz); ESI-HRMS (*m*/*z*) calculated for [M+H]^+^ ion species C_13_H_16_FN_3_O_3_S 313.3474, found 313.3486.

**4-(4-Acryloyl-1,4-diazepan-1-yl)benzenesulfonamide** **(45a)**

Light yellow solid 27% yield; ^1^H NMR (DMSO-*d_6_*, 400 MHz): 7.61 (2H, m), 7.00 (2H, bs, SO_2_N*H*_2_, exchange with D_2_O), 6.88 (2H, t, *J* = 8.90 Hz), 6.74 (1H, m), 6.08 (1H, m), 5.66 (1H, m), 3.73 (4H, m), 3.61 (2H, m), 3.45 (2H, m), 1.87 (2H, m); ^13^C NMR (DMSO-*d_6_*, 100 MHz): 165.9, 150.3, 129.3, 129.0, 128.5, 127.9, 111.5, 50.7, 49.6, 48.1, 46.7, 45.4; ESI-HRMS (*m*/*z*) calculated for [M+H]^+^ ion species C_14_H_19_N_3_O_3_S, 309.3840, found 309.3856.

**4-(4-Acryloyl-1,4-diazepan-1-yl)-3-chlorobenzenesulfonamide** **(45b)**

White solid 86% yield; ^1^H NMR (DMSO-*d_6_*, 400 MHz): 7.86 (1H, s), 7.76 (1H, d, *J* = 8.12 Hz), 7.41 (2H, bs, SO_2_N*H*_2_, exchange with D_2_O), 7.33 (1H, d, *J* = 7.42 Hz), 6.89 (1H, dd, *J* = 9.10 Hz; *J* = 2.28 Hz), 6.19 (1H, d, *J* = 16.28 Hz), 5.76 (1H, d, *J* = 9.68 Hz), 3.35 (4H, m), 2.94 (2H, m), 2.87 (2H, t, *J* = 5.94 Hz), 1.87 (2H, m); ^13^C NMR (DMSO-*d_6_*, 100 MHz): 153.5, 146.7, 139.5, 136.1, 135.4, 133.3, 131.5, 126.3, 116.0, 54.8, 51.5, 48.8, 47.1, 28.2; ESI-HRMS (*m*/*z*) calculated for [M+H]^+^ ion species C_14_H_18_ClN_3_O_3_S 343.8260, found 343.8258.

**4-(4-Acryloyl-1,4-diazepan-1-yl)-3-fluorobenzenesulfonamide** **(45c)**

Light orange oil 47% yield; ^1^H NMR (DMSO-*d_6_*, 400 MHz): 7.48 (1H, s), 7.45 (1H, s), 7.24 (2H, s), 7.08 (1H, m), 6.77 (1H, m), 6.08 (1H, dd, *J* = 48.3 Hz; *J* = 16.32 Hz), 5.66 (1H, dd, *J* = 31.02 Hz; *J* = 10.34 Hz), 3.74 (2H, m), 3.62 (2H, m), 3.54 (4H, m), 1.88 (2H, t, *J* = 5.34 Hz); ^13^C NMR (DMSO-*d_6_*, 100 MHz): 162.7, 153.6 (d, *J*^1^_C-F_ = 241.0 Hz), 139.4 (d, *J*^3^_C-F_ = 6.90 Hz), 133.2 (d, *J*^3^_C-F_ = 6.48 Hz), 131.5, 128.8, 127.7, 117.5 (d, *J*^3^_C-F_ = 4.66 Hz), 113.8 (d, *J*^2^_C-F_ = 25.44 Hz), 54.8 (d, *J*^3^_C-F_ = 5.01 Hz), 51.5 (d, *J*^3^_C-F_ = 5.34 Hz), 50.3, 48.2, 21.6; ESI-HRMS (*m*/*z*) calculated for [M+H]^+^ ion species C_14_H_18_FN_3_O_3_S 327.3744, found 327.3752.

**4-(4-Acryloyl-1,4-diazepan-1-yl)-3,5-difluorobenzenesulfonamide** **(45d)**

Light orange oil 52% yield; ^1^H NMR (DMSO-*d_6_*, 400 MHz): 7.49 (2H, bs, SO_2_N*H*_2_, exchange with D_2_O), 7.46 (1H, s), 7.43 (1H, s), 6.83 (1H, m), 6.17 (1H, m), 5.71 (1H, m), 3.72 (4H, m), 3.49 (2H, m), 3.34 (2H, m), 1.87 (2H, m); ^13^C NMR (DMSO-*d_6_*, 100 MHz): 168.5, 158.2, 156.7 (dd, *J*^1^_C-F_ = 248.15; 7.94 Hz), 139.5 (dt, *J*^2^_C-F_ = 25.08; 10.35 Hz), 132.8, 128.5 (d, *J*^3^_C-F_ = 9.18 Hz), 111.4 (d, *J*^3^_C-F_ = 9.21 Hz), 54.9, 51.5, 50.3, 49.8, 30.4; ESI-HRMS (*m*/*z*) calculated for [M+H]^+^ ion species C_14_H_17_F_2_N_3_O_3_S 345.3648, found 345.3660.

**4-Sulfamoylphenyl** **acrylate** **(46)**

Light yellow solid 69% yield; ^1^H NMR (DMSO-*d_6_*, 400 MHz): 7.92 (2H, d, *J* = 8.32 Hz), 7.46 (2H, d, *J* = 6.64 Hz), 7.44 (2H, bs, SO_2_N*H*_2_, exchange with D_2_O), 6.61 (1H, d, *J* = 17.21 Hz), 6.48 (1H, dd, *J* = 17.17 Hz; *J* = 10.26 Hz), 6.24 (1H, d, *J* = 10.24 Hz); ^13^C NMR (DMSO-*d_6_*, 100 MHz): 164.8, 153.4, 142.7, 135.2, 128.3, 128.3, 132.4; ESI-HRMS (*m*/*z*) calculated for [M+H]^+^ ion species C_9_H_9_NO_4_S 227.2340, found 227.2348.

**General** **procedure** **for** **the** **synthesis** **of** **compounds** **53a-d and 54a-d**

In a flask the appropriate sulfonamide (**51a-e**) (1.0 equiv) and piperazine (**52a**) or homopiperazine (**52b**) (3.0 equiv) were suspended in H_2_O (50 mL). The reaction mixture was stirred at reflux temperature overnight. A control via TLC was performed to assure the complete consumption of the starting materials. The reaction was cooled to room temperature and a precipitate was formed, filtered off under vacuum, washed with Et_2_O and H_2_O, dried on air and used as it is.

**4-(Piperazin-1-yl)benzenesulfonamide** **(53a)**

White solid 42% yield; ^1^H NMR (DMSO-*d_6_*, 400 MHz): 7.65 (2H, d, *J* = 8.65 Hz), 7.06 (2H, bs, SO_2_N*H*_2_, exchange with D_2_O), 7.03 (2H, d, *J* = 8.81 Hz), 3.21 (4H, m), 2.85 (4H, m); ESI-HRMS (*m*/*z*) calculated for [M+H]^+^ ion species C_10_H_15_N_3_O_2_S 241.3090, found 241.3082.

**3-Chloro-4-(piperazin-1-yl)benzenesulfonamide** **(53b)**

White solid 88% yield; ^1^H NMR (DMSO-*d_6_*, 400 MHz): 7.85 (1H, m, N*H*, exchange with D_2_O), 7.74 (1H, d, *J* = 8.45 Hz), 7.43 (2H, m), 7.29 (2H, bs, SO_2_N*H*_2_, exchange with D_2_O), 2.99 (4H, m), 2.88 (4H, m); ^13^C NMR (DMSO-*d_6_*, 100 MHz): 155.8, 131.4, 129.6, 128.3, 124.7, 117.0, 52.3, 47.0; ESI-HRMS (*m*/*z*) calculated for [M+H]^+^ ion species C_10_H_14_ClN_3_O_2_S 275.7510, found 275.7518.

**4-(Piperazin-1-yl)-3-(trifluoromethyl)benzenesulfonamide** **(53c)**

White solid 52% yield; ^1^H NMR (DMSO-*d_6_*, 400 MHz): 8.10 (1H, s), 8.07 (1H, d, *J* = 8.55 Hz), 7.64 (1H, d, *J* = 8.47 Hz), 7.44 (2H, bs, SO_2_N*H*_2_, exchange with D_2_O), 2.89 (4H, m), 2.86 (4H, m); ^13^C NMR (DMSO-*d_6_*, 100 MHz): 156.3, 140.1, 131.8, 128.6, 126.2 (q, *J* = 5.48 Hz), 125.4 (q, *J* = 273.93 Hz), 125.2, 55.0, 46.6; ESI-HRMS (*m*/*z*) calculated for [M+H]^+^ ion species C_11_H_14_F_3_N_3_O_2_S 309.3072, found 309.3064.

**3-Fluoro-4-(piperazin-1-yl)benzenesulfonamide** **(53d)**

White solid 99% yield; ^1^H NMR (DMSO-*d_6_*, 400 MHz): 7.53 (1H, dd, *J* = 8.60 Hz; *J* = 2.0 Hz), 7.48 (1H, dd, *J* = 15.0 Hz; *J* = 2.0 Hz), 7.3 (2H, bs, SO_2_N*H*_2_, exchange with D_2_O), 7.13 (1H, dd, *J* = 8.6 Hz; *J* = 8.6 Hz), 3.35 (1H, s), 3.03 (4H, m), 2.83 (4H, m); ESI-HRMS (*m*/*z*) calculated for [M+H]^+^ ion species C_10_H_14_FN_3_O_2_S 259.2994, found 260.08.

**4-(1,4-Diazepan-1-yl)benzenesulfonamide** **(54a)**

White solid 36% yield; ^1^H NMR (DMSO-*d_6_*, 400 MHz): 7.59 (2H, d, *J* = 8.82 Hz), 6.97 (2H, bs, SO_2_N*H*_2_, exchange with D_2_O), 6.80 (2H, d, *J* = 8.91 Hz), 3.62 (2H, t, *J* = 6.06 Hz), 3.54 (2H, m), 2.87 (2H, m), 2.63 (2H, t, *J* = 5.77 Hz), 1.79 (2H, m); ^13^C NMR (DMSO-*d_6_*, 100 MHz): 151.4, 130.3, 128.4, 111.2, 53.1, 48.6, 48.4, 48.1, 29.3; ESI-HRMS (*m*/*z*) calculated for [M+H]^+^ ion species C_11_H_17_N_3_O_2_S 255.3360, found 255.3368.

**3-Chloro-4-(1,4-diazepan-1-yl)benzenesulfonamide** **(54b)**

White solid 56% yield; ^1^H NMR (DMSO-*d_6_*, 400 MHz): 7.76 (1H, d, *J* = 2.16 Hz), 7.64 (1H, dd, *J* = 8.60 Hz; *J* = 2.15 Hz), 7.38 (1H, d, *J* = 8.72 Hz), 7.29 (2H, bs, SO_2_N*H*_2_, exchange with D_2_O); 3.53 (1H, m), 3.35 (4H, m), 2.94 (2H, m), 2.87 (2H, t, *J* = 5.94 Hz), 1.87 (2H, m); ^13^C NMR (DMSO-*d_6_*, 100 MHz): 153.7, 137.2, 129.2, 126.2, 125.3, 121.3, 57.4, 53.3, 50.1, 48.5, 31.4; ESI-HRMS (*m*/*z*) calculated for [M+H]^+^ ion species C_11_H_16_ClN_3_O_2_S 289.7780, found 289.7792.

**4-(1,4-Diazepan-1-yl)-3-fluorobenzenesulfonamide** **(54c)**

White solid 81% yield; ^1^H NMR (DMSO-*d_6_*, 400 MHz): 7.47 (1H, s), 7.44 (2H, d, *J* = 6.70 Hz),7.20 (2H, bs, SO_2_N*H*_2_, exchange with D_2_O), 7.02 (1H, t, *J* = 9.09 Hz), 3.57 (2H, t, *J* = 5.53 Hz), 3.50 (2H, m), 2.91 (2H, m), 2.73 (2H, m), 1.81 (2H, m); ^13^C NMR (DMSO-*d_6_*, 100 MHz): 151.2 (d, *J*^1^_C-F_ = 243.16 Hz), 141.9 (d, *J*^3^_C-F_ = 6.90 Hz), 132.8 (d, *J*^3^_C-F_ = 6.48 Hz), 123.9, 117.0 (d, *J*^3^_C-F_ = 4.66 Hz), 115.3 (d, *J*^2^_C-F_ = 25.44 Hz), 55.5 (d, *J*^3^_C-F_ = 5.01 Hz), 51.3 (d, *J*^3^_C-F_ = 5.34 Hz), 49.8, 48.4, 31.1; ESI-HRMS (*m*/*z*) calculated for [M+H]^+^ ion species C_11_H_16_FN_3_O_2_S 273.3264, found 273.3192.

**4-(1,4-Diazepan-1-yl)-3,5-difluorobenzenesulfonamide** **(54d)**

Light orange solid 95% yield; ^1^H NMR (DMSO-*d_6_*, 400 MHz): 7.42 (2H, d, *J* = 9.47 Hz), 5.59 (2H, bs, SO_2_N*H*_2_, exchange with D_2_O); 3.43 (2H, t, *J* = 5.63 Hz), 3.37 (2H, m), 2.87 (4H, m), 1.80 (2H, m); ^13^C NMR (DMSO-*d_6_*, 100 MHz): 156.3 (dd, *J*^1^_C-F_ = 248.15; 7.94 Hz), 135.0 (dt, *J*^2^_C-F_ = 25.08; 10.35 Hz), 111.4 (d, *J*^3^_C-F_ = 9.18 Hz), 111.2 (d, *J*^3^_C-F_ = 9.21 Hz), 57.5, 53.4, 50.9, 58.7, 32.4; ESI-HRMS (*m*/*z*) calculated for [M+H]^+^ ion species C_11_H_15_F_2_N_3_O_2_S 291.3168, found 291.3194.

**Table S1.** Mass spectra of the main peaks (>1% of the dominant peak) were compared with NIST database spectra to identify each component Compounds in green identified with probability <20%; Compounds in yellow 2^nd^ library hit identified with probability below 20%

| **SAMPLE** | **Rt (Min)** | **BASE ION** | **MASS ION** | **POSSIBLE COMPOUND** |
| --- | --- | --- | --- | --- |
| 1 | 22.11 | 69 | 456 | Cholesta-8,24-dien-3-yl TMS Derivative |
|  | 22.62 | 69 | 468 | Ergosterol TMS Derivative |
|  | 23.40 | 75 | 470 | Ergosta-7,22-dien-3-yl TMS Derivative |
|  | 24.02 | 73 | 470 | 4-Methylcholesta-8,24-dien-3β-yl TMS Derivative |
|  | 24.31 | 75 | 470 | Ergosta-7,22-dien-3-yl TMS Derivative |
|  | 25.18 | 69 | 498 | Lanosterol TMS Derivative |
| 2 | 21.97 | 73 | 481? | 1,1,2,2-Tetrahydro-1,1-dimethoxy Lycopene |
|  | 22.56 | 69 | 468 | Ergosterol TMS Derivative |
|  | 23.19 | 69 | 484 | 4-Methyl-ergosta-7,24(28)-dien-3-yl TMS Derivative |
|  | 23.78 | 69 | 484 | 4,4-Dimethylcholesta-8,24-dien-3-yl TMS Derivative |
|  | 24.29 | 55 | 436 | Ergosta-7,22-dien-3-yl TMS Derivative |
|  | 24.94 | 73 | 558? | 1,1,2,2-Tetrahydro-1,1-dimethoxy Lycopene |
|  | 25.23 | 69 | 498 | Lanosterol TMS Derivative |
|  | 26.22 | 73 | 498? | Gorgost-5-en-3-yl TMS Derivative |
|  | 26.74 | 69 | 512? | 4,14-Dimethyl-9,19-cycloergost-24(28)-en-3-ol acetate |
| 3 | 22.62 | 69 | 468 | Ergosterol TMS Derivative |
|  | 23.77 | 69 | 484 | 4,4-Dimethylcholesta-8,24-dien-3-yl TMS Derivative |
|  | 24.89 | 73 | 467? | 1,1,2,2-Tetrahydro-1,1-dimethoxy Lycopene |
|  | 25.20 | 69 | 498 | Lanosterol TMS Derivative |
|  | 26.70 | 69 | 512? | 4,14-Dimethyl-9,19-cycloergost-24(28)-en-3-ol acetate |
| 4 | 22.60 | 69 | 468 | Ergosterol TMS Derivative |
|  | 23.77 | 69 | 484 | 4,4-Dimethylcholesta-8,24-dien-3-yl TMS Derivative |
|  | 25.20 | 69 | 498 | Lanosterol TMS Derivative |
|  | 26.71 | 73 | 512? | 4,14-Dimethyl-9,19-cycloergost-24(28)-en-3-ol acetate |
| 5 | 22.65 | 69 | 468 | Ergosterol TMS Derivative |
|  | 23.77 | 69 | 484 | 4,4-Dimethylcholesta-8,24-dien-3-yl TMS Derivative |
|  | 25.21 | 69 | 498 | Lanosterol TMS Derivative |
|  | 26.69 | 73 | 512? | 4,14-Dimethyl-9,19-cycloergost-24(28)-en-3-ol acetate |
| 6 | 22.60 | 69 | 468 | Ergosterol TMS Derivative |
|  | 23.75 | 69 | 484 | 4,4-Dimethylcholesta-8,24-dien-3-yl TMS Derivative |
|  | 25.18 | 69 | 498 | Lanosterol TMS Derivative |
| 7 | 22.60 | 69 | 468 | Ergosterol TMS Derivative |
|  | 23.75 | 69 | 484 | 4,4-Dimethylcholesta-8,24-dien-3-yl TMS Derivative |
|  | 25.19 | 69 | 498 | Lanosterol TMS Derivative |
| 8 | 21.97 | 69 | 480 | Ergosta-5,7,22,24(28)-tetraene TMS Derivative |
|  | 22.54 | 69 | 468 | Ergosterol TMS Derivative |
|  | 23.15 | 69 | 484 | Stigmasterol TMS Derivative |
|  | 23.76 | 69 | 484 | 4,4-Dimethylcholesta-8,24-dien-3-yl TMS Derivative |
|  | 24.89 | 73 | 558? | 1,1,2,2-Tetrahydro-1,1-dimethoxy Lycopene |
|  | 25.20 | 69 | 498 | Lanosterol TMS Derivative |
|  | 26.74 | 69 | 512? | 4,14-Dimethyl-9,19-cycloergost-24(28)-en-3-ol acetate |
| 9 | 22.59 | 69 | 468 | Ergosterol TMS Derivative |
|  | 23.74 | 69 | 484 | 4,4-Dimethylcholesta-8,24-dien-3-yl TMS Derivative |
|  | 25.18 | 69 | 498 | Lanosterol TMS Derivative |
|  | 26.66 | 73 | 512? | 4,14-Dimethyl-9,19-cycloergost-24(28)-en-3-ol acetate |
| 10 | 22.59 | 69 | 468 | Ergosterol TMS Derivative |
|  | 23.76 | 69 | 484 | 4,4-Dimethylcholesta-8,24-dien-3-yl TMS Derivative |
|  | 25.19 | 69 | 498 | Lanosterol TMS Derivative |
|  | 26.69 | 69 | 512? | 4,14-Dimethyl-9,19-cycloergost-24(28)-en-3-ol acetate |

**Table S2:** The peak area of each identified peak was used to calculate their percentages in relation to the total peak areas of all identified peaks.

| **SAMPLE** | **Rt (Min)** | **COMPOUND** | **PEAK AREA** | **(%)** |
| --- | --- | --- | --- | --- |
| **1** | 22.11 | Cholesta-8,24-dien-3-yl TMS Derivative | 99223560 | 0.4 |
|  | 22.62 | Ergosterol TMS Derivative | 22341108342 | 90.6 |
|  | 23.40 | Ergosta-7,22-dien-3-yl TMS Derivative | 347980107 | 1.4 |
|  | 24.02 | 4-Methylcholesta-8,24-dien-3β-yl TMS Derivative | 620507076 | 2.5 |
|  | 24.31 | Ergosta-7,22-dien-3-yl TMS Derivative | 327000111 | 1.3 |
|  | 25.18 | Lanosterol TMS Derivative | 934410720 | 3.8 |
| **2** | 21.97 | 1,1,2,2-Tetrahydro-1,1-dimethoxy Lycopene | 294707934 | 2.4 |
|  | 22.56 | Ergosterol TMS Derivative | 1279937824 | 10.3 |
|  | 23.19 | 4-Methyl-ergosta-7,24(28)-dien-3-yl TMS Derivative | 716322037 | 5.8 |
|  | 23.78 | 4,4-Dimethylcholesta-8,24-dien-3-yl TMS Derivative | 509791037 | 4.1 |
|  | 24.29 | Ergosta-7,22-dien-3-yl TMS Derivative | 101163786 | 0.8 |
|  | 24.94 | 1,1,2,2-Tetrahydro-1,1-dimethoxy Lycopene | 2145649411 | 17.3 |
|  | 25.23 | Lanosterol TMS Derivative | 4386983978 | 35.4 |
|  | 26.22 | Gorgost-5-en-3-yl TMS Derivative | 103458742 | 0.8 |
|  | 26.74 | 4,14-Dimethyl-9,19-cycloergost-24(28)-en-3-ol acetate | 2847630595 | 23.0 |
| **3** | 22.62 | Ergosterol TMS Derivative | 20341160768 | 83.3 |
|  | 23.77 | 4,4-Dimethylcholesta-8,24-dien-3-yl TMS Derivative | 747947155 | 3.1 |
|  | 24.89 | 1,1,2,2-Tetrahydro-1,1-dimethoxy Lycopene | 268104404 | 1.1 |
|  | 25.20 | Lanosterol TMS Derivative | 2556447374 | 10.5 |
|  | 26.70 | 4,14-Dimethyl-9,19-cycloergost-24(28)-en-3-ol acetate | 503354300 | 2.1 |
| 4 | 22.60 | Ergosterol TMS Derivative | 15423616115 | 78.8 |
|  | 23.77 | 4,4-Dimethylcholesta-8,24-dien-3-yl TMS Derivative | 693774377 | 3.5 |
|  | 25.20 | Lanosterol TMS Derivative | 3054069854 | 16.6 |
|  | 26.71 | 4,14-Dimethyl-9,19-cycloergost-24(28)-en-3-ol acetate | 399548223 | 2.0 |
| 5 | 22.65 | Ergosterol TMS Derivative | 26030139965 | 84.1 |
|  | 23.77 | 4,4-Dimethylcholesta-8,24-dien-3-yl TMS Derivative | 624586523 | 2.0 |
|  | 25.21 | Lanosterol TMS Derivative | 4016582220 | 13.0 |
|  | 26.69 | 4,14-Dimethyl-9,19-cycloergost-24(28)-en-3-ol acetate | 265817412 | 0.9 |
| 6 | 22.60 | Ergosterol TMS Derivative | 16810751334 | 84.0 |
|  | 23.75 | 4,4-Dimethylcholesta-8,24-dien-3-yl TMS Derivative | 325079232 | 1.6 |
|  | 25.18 | Lanosterol TMS Derivative | 2882300312 | 14.4 |
| 7 | 22.60 | Ergosterol TMS Derivative | 18705528718 | 73.2 |
|  | 23.75 | 4,4-Dimethylcholesta-8,24-dien-3-yl TMS Derivative | 2759174484 | 10.8 |
|  | 25.19 | Lanosterol TMS Derivative | 4073971667 | 16.0 |
| 8 | 21.97 | Ergosta-5,7,22,24(28)-tetraene TMS Derivative | 56296663 | 0.6 |
|  | 22.54 | Ergosterol TMS Derivative | 2025002192 | 21.5 |
|  | 23.15 | Stigmasterol TMS Derivative | 55056364 | 0.6 |
|  | 23.76 | 4,4-Dimethylcholesta-8,24-dien-3-yl TMS Derivative | 551057002 | 5.9 |
|  | 24.89 | 1,1,2,2-Tetrahydro-1,1-dimethoxy Lycopene | 103930064 | 1.1 |
|  | 25.20 | Lanosterol TMS Derivative | 3908516345 | 41.6 |
|  | 26.74 | 4,14-Dimethyl-9,19-cycloergost-24(28)-en-3-ol acetate | 2704352197 | 28.8 |
| 9 | 22.59 | Ergosterol TMS Derivative | 15745119094 | 80.3 |
|  | 23.74 | 4,4-Dimethylcholesta-8,24-dien-3-yl TMS Derivative | 369643075 | 1.9 |
|  | 25.18 | Lanosterol TMS Derivative | 3274224572 | 16.7 |
|  | 26.66 | 4,14-Dimethyl-9,19-cycloergost-24(28)-en-3-ol acetate | 215127063 | 1.1 |
| 10 | 22.59 | Ergosterol TMS Derivative | 15683750516 | 78.3 |
|  | 23.76 | 4,4-Dimethylcholesta-8,24-dien-3-yl TMS Derivative | 544327690 | 2.7 |
|  | 25.19 | Lanosterol TMS Derivative | 3412312937 | 17.0 |
|  | 26.69 | 4,14-Dimethyl-9,19-cycloergost-24(28)-en-3-ol acetate | 386214336 | 1.9 |

**References**

1. Giovannuzzi, S.; D'Ambrosio, M.; Luceri, C.; Osman, S- M.; Pallecchi, M.; Bartolucci, G.; Nocentini, A.; Supuran, C. T. Aromatic Sulfonamides including a Sulfonic Acid Tail: New Membrane Impermeant Carbonic Anhydrase Inhibitors for Targeting Selectively the Cancer-Associated Isoforms. *Int. J. Mol. Sci*. **2021**, *23*, 461.
2. Marinacci, B.; D'Agostino, I.; Angeli, A.; Carradori, S.; Melfi, F.; Grande, R.; Corsiani, M.; Ferraroni, M.; Agamennone, M.; Tondo, A. R.; Zara, S.; Puca, V.; Pellegrini, B.; Vagaggini, C.; Dreassi, E.; Patrauchan, M. A.; Capasso, C.; Nicolotti, O.; Carta, F.; Supuran, C. T. Inhibition of *Pseudomonas aeruginosa* Carbonic Anhydrases, Exploring Ciprofloxacin Functionalization Toward New Antibacterial Agents: An In-Depth Multidisciplinary Study. *J Med Chem*. **2024**, *21*, 19077
3. Bozdag, M.; Alafeefy, A, M.; Altamimi, A. M.; Carta, F.; Supuran, C. T., Vullo, D. Synthesis of new 3-(2-mercapto-4-oxo-4H-quinazolin-3-yl)-benzenesulfonamides with strong inhibition properties against the tumor associated carbonic anhydrases IX and XII. *Bioorg Med Chem*. **2010,** *10*, 2782.
4. Ram, S.; Celik, G.; Khloya, P.; Vullo, D.; Supuran, C. T.; Sharma, P. K. Benzenesulfonamide bearing 1,2,4-triazole scaffolds as potent inhibitors of tumor associated carbonic anhydrase isoforms hCA IX and hCA XII. *Bioorg Med Chem*. **2014**, *6*, 1873.
5. Turkmen, H.; Durgun, M.; Yilmaztekin, S.; Emul, M.; Innocenti, I.; Vullo, D.; Scozzafava, A.; Supuran, C. T. Carbonic anhydrase inhibitors. Novel sulfanilamide/acetazolamide derivatives obtained by the tail approach and their interaction with the cytosolic isozymes I and II, and the tumor-associated isozyme IX. *Bioorg Med Chem Lett*. **2005**, *2*, 367.
6. China Pharmaceutical University. CN105884712, 2016 A
